# Systematic review of the effect of policies to restrict the marketing of foods and non‐alcoholic beverages to which children are exposed

**DOI:** 10.1111/obr.13447

**Published:** 2022-04-05

**Authors:** Emma Boyland, Lauren McGale, Michelle Maden, Juliet Hounsome, Angela Boland, Andrew Jones

**Affiliations:** ^1^ Department of Psychology University of Liverpool Liverpool UK; ^2^ Department of Psychology Edge Hill University Ormskirk UK; ^3^ Liverpool Reviews and Implementation Group University of Liverpool Liverpool UK

**Keywords:** children, exposure, food marketing, policy

## Abstract

This systematic review examined the effectiveness of policies restricting the marketing of foods and/or non‐alcoholic beverages to children to inform updated World Health Organization (WHO) guidelines. Databases were searched to March 2020. Inclusion criteria were primary studies of any design assessing implemented policies to restrict food marketing to children (0–19 years). Critical outcomes were exposure to and power of marketing, dietary intake, choice, preference, and purchasing. Important outcomes were purchase requests, dental caries, body weight, diet‐related noncommunicable diseases, product change, and unintended consequences. Forty‐four observational studies met inclusion criteria; most were moderate quality. Pooling was conducted using vote counting by direction of effect, and GRADE was used to judge evidence certainty. Evidence suggests food marketing policies may result in reduced purchases of unhealthy foods and in unintended consequences favorable for public health. Desirable or potentially desirable (for public health) effects of policies on food marketing exposure and power were also found. Evidence on diet and product change was very limited. The certainty of evidence was very low for four outcomes (exposure, power, dietary intake, and product change) and low for two (purchasing and unintended consequences). Policies can effectively limit food marketing to children; policymakers should prioritize mandatory approaches aligned with WHO recommendations.

## 1 INTRODUCTION

Globally, food and non‐alcoholic beverage (hereafter, food) marketing is pervasive across multiple media and formats and predominantly promotes products high in fat, sugar, and/or salt (HFSS) and their associated brands.^1^, ^2^, ^3^, ^4^ Food marketing influences children's eating and related behaviors such as purchase requests, purchases, and preferences.^5^, ^6^, ^7^, ^8^ Evidence for a relationship between food marketing exposure and obesity meets epidemiological criteria for causality.^9^ It is thought to be the combination of salient food cues^10^ and creative content (e.g., branding, promotional characters, emotional appeals, and animation) in food marketing that produces such compelling commercial messages so as to influence children's behavior and health outcomes.^11^ In other words, the impact of food marketing is a function of both exposure to the marketing message and its persuasive power.^12^

Given this evidence of impact, and with diet‐related noncommunicable disease (NCD) risk and obesity prevention in children being public health priorities in many countries internationally, best‐practice recommendations have been issued by the World Health Organization (WHO) and other authoritative bodies for governments and industry to restrict HFSS food marketing to children. In May 2010, the World Health Assembly unanimously adopted the WHO Set of Recommendations on the Marketing of Foods and Non‐alcoholic Beverages to Children through resolution WHA63.14.^13^ The primary purpose of these recommendations was to guide Member States in the optimal design of new policies, or in strengthening existing policies, to maximize the achievement of public health goals. Also in response to the mandate of that resolution, WHO published a framework for policymakers to support the implementation of recommendations in individual territories,^12^ and WHO have led on the development of region‐specific nutrient‐profiling models to support policymakers in identifying products that should be restricted in marketing to children.^11^

Implementation of the WHO recommendations so far has been limited, with a lack of comprehensive approaches.^14^ Numerous food industry groups have established self‐regulatory programs that refer to encouraging more “responsible advertising” while a small but growing number of countries have enacted mandatory policies.^15^ To date, focused evaluations have suggested that self‐regulation has not meaningfully reduced children's exposure to unhealthy food marketing^16^ or sales of unhealthy foods.^17^ Similarly, the few existing assessments of mandatory policies have reported mixed findings as to whether not the policies resulted in reductions in unhealthy food advertising in affected media^15^ although effects on unhealthy food sales have been reported.^17^ In some studies, decreases in HFSS advertising covered by the policy were accompanied by increases in HFSS advertising *not* covered by the policy such that overall exposure did not substantially change.^15^

There is an urgent need to comprehensively evaluate the effectiveness of existing policies against a range of relevant indicators^12^ (behavior and health but also market responses and consequences for wider society) and specifically to identify which policy design elements are most effective at achieving meaningful improvements.^15^ Therefore, WHO commissioned the current review to inform the development of updated recommendations regarding policies to restrict food marketing to children.

## 2 METHODS

We conducted a systematic review following Cochrane methods^18^ reported as per Preferred Reporting Items for Systematic Reviews and Meta‐Analyses.^19^ The WHO Nutrition Guidance Expert Advisory Group Subgroup (NUGAG) on Policy Actions determined the research question, policy types, and outcomes to be captured by the review and ranked all outcomes for priority (see Supporting Information). Key terms were used as defined by WHO, namely, “marketing” as a commercial communication, “exposure” as the reach or frequency of the marketing message, and “power” as the creative content of marketing.^12^ Policies were defined as either mandatory (legally enforceable measures including statutory approaches, regulations, legislation, or any “order” used by a jurisdiction's legal system) or nonmandatory (including self‐regulatory measures, pledges, or codes). The protocol was pre‐registered with Prospero in May 2019 (CRD42019132506, available from https://www.crd.york.ac.uk/prospero/display_record.php?RecordID=132506).

### 2.1 Search strategy and selection criteria

Primary studies (of any design) were considered for inclusion if they assessed implemented policies that aim to restrict (i.e., to reduce exposure and/or power of) food marketing to children aged 0–19 years compared with no policy (e.g., before the policy was implemented) or a weaker policy (e.g., partially implemented) and reported on one or more outcome of interest. Exclusion criteria were reviews of studies (narrative or systematic) and studies assessing action plans, strategies, programs, initiatives, or potential impact of policies yet to be implemented. Critical outcomes (critical for decision making^20^) comprised exposure to food marketing, power of food marketing, and food intake, choice, preferences, or purchasing (by children or on behalf of children). Important outcomes (important but not critical for decision making^20^) were purchase requests (by children to a caregiver), dental caries/erosion, body weight/body mass index (BMI)/obesity, diet‐related NCDs (including validated surrogate indicators), product change (e.g., portion size and product reformulation), and unintended consequences to wider society (e.g., revenue and jobs).

Searches were conducted in March 2019 and updated in March 2020 by an information specialist (MM). We searched MEDLINE, CINAHL, Web of Science, EMBASE, ERIC, The Cochrane Library (CDSR, CENTRAL), Business Source Complete, EconLit, Emerald, JSTOR, HMIC, Advertising Education Forum, The Campbell Library, Database of Promoting Health Effectiveness Reviews (DoPHER), Healthevidence.org, TRIP, IRIS, Global Index Medicus, KOREAMED, Communication & Mass Media Complete, Academic Search Complete, and Index to Legal Periodicals & Books Full Text (H.W. Wilson). Targeted searches of Google and Google Scholar were also performed. The search strategy is provided in the Supporting Information. Searches were peer reviewed (checked for appropriateness by three researchers and a WHO librarian).

Database searches were supplemented with (i) hand searching reference lists of retrieved systematic reviews and eligible studies, (ii) contact with topic experts, (iii) forward and backward citation searching of included studies, and (iv) a WHO evidence call for data.^21^ No language or date restrictions were applied.

Two reviewers independently screened all studies against inclusion criteria: assessing titles and abstracts to identify potentially relevant studies then assessing those full texts. Titles and abstracts of articles published in languages other than English were screened using Google Translate, then researchers proficient in both languages translated full texts for review. Disagreement was resolved through consensus and, if necessary, by consulting a third reviewer. The search and screening processes were combined for this and a parallel review on the impact of food marketing on children's eating behaviors and health (CRD42019137993).

### 2.2 Quality assessment

There is no established tool for the assessment of quality for observational studies evaluating policy effectiveness, so an adapted version of the Newcastle–Ottawa Scale (NOS) was applied. Modifications were the removal of nonapplicable characteristics (e.g., selection of the non‐exposed cohort) or components of characteristics (e.g., sample size calculations). Point allocations for outcome measures were not altered. Bias assessments were conducted by one reviewer and independently checked by a second. Discrepancies were discussed until a consensus was reached.

### 2.3 Data extraction

Two reviewers (EB, LM) independently extracted data using pre‐piloted forms, and again, discrepancies were discussed until a consensus was reached. The reviewers extracted the following information: study information (e.g., authors, year, study country, funding, and conflicts of interest); study design (e.g., description of study design and media assessed [if relevant]); population (where relevant, e.g., number of participants in intervention and control groups); intervention (e.g., policy type, scope, definitions, and level of implementation); outcome measures (e.g., volume of marketing).

### 2.4 Data synthesis and analysis

It was not possible to conduct formal quantitative analyses for any outcome because of the diverse range of effect measures used and the limited reporting of *p* values or the data required for the computation of effect sizes.^22^ Therefore, vote counting based on direction of effect was adopted.^22^ This necessitated the selection of one effect per outcome per study, and decision rules were used to determine the most appropriate effect (namely, the most comprehensive measure, e.g., overall unhealthy food marketing instead of marketing of individual food groups).

Five categories of effect direction were used:

- *Clear effect favoring the intervention*, where the effect estimate favors the intervention and the 95% confidence interval (CI) excludes the null;
- *Unclear effect potentially favoring the intervention*, where the effect estimate favors intervention but the 95% CI includes the null and is wide;
- *No difference in effect*, where the 95% CI crosses the null but is narrow;
- *Unclear effect potentially favoring the control*, where the effect estimate favors the control but the 95% CI includes the null and is wide; and
- *Clear effect favoring the control*, where the effect estimate favors the control and the 95% CI excludes the null.

Effects (i) and (ii) were considered desirable (clear or potential public health benefit); effects (iv) and (v) were considered undesirable (clear or potential public health harm). Categorization was based upon effect estimates, CIs, and *p* values and guided by author reporting. The binomial probability test was applied to the (1) number of effects clearly favoring the intervention and (2) number of effects potentially favoring the intervention, each compared with the number of effects clearly favoring the control, potentially favoring the control, or showing no effect. Significant *p* values can represent either a significantly *smaller* proportion of desirable effects for public health or a significantly *larger* proportion of desirable effects for public health compared with effects in the other categories. A nonsignificant *p* value is indicative of no significant differences in the proportions. Narrow CIs reflect more precise estimates of the proportion of interventions with desirable effects, due to increased number of studies in the analysis. Further details of this approach are provided in the supplement, and analysis files are available here: https://osf.io/4fk2m/. Where possible, subgroup analyses were used to identify the most effective policy design elements.

We used GRADE^23^ to judge the certainty of evidence as high, moderate, low, or very low (see Supporting Information). Certainty of evidence was assessed by the research team and revised as necessary following discussion with the WHO NUGAG.

## 3 RESULTS

### 3.1 Description of included studies

**FIGURE 1:**
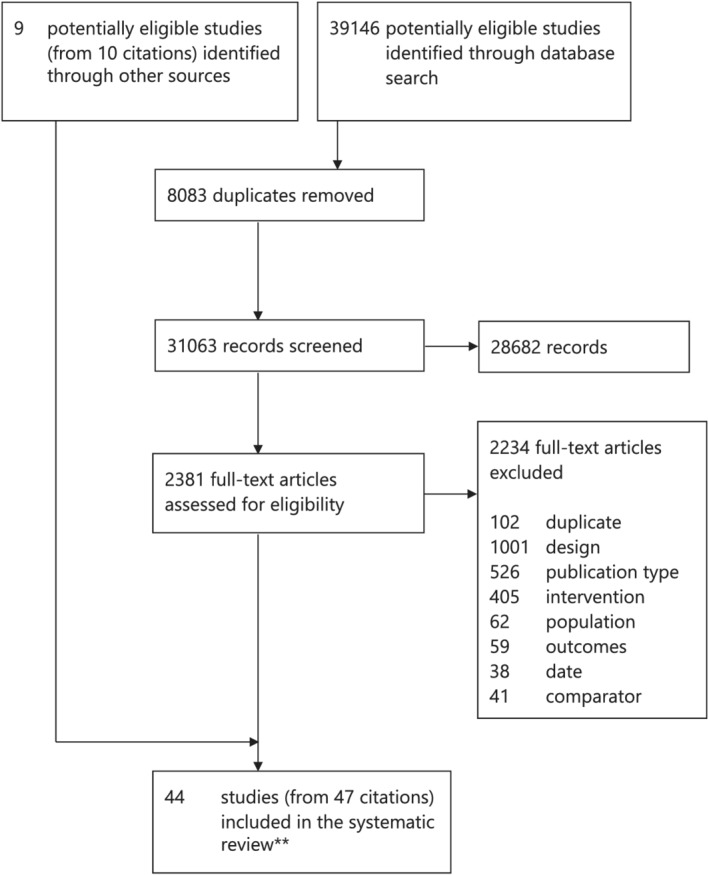
Study selection. *Reasons for exclusion: incorrect intervention, comparator, population, or date, duplicate records. **The search and screening processes were combined for this and a parallel review on the impact of food marketing on children's eating behaviors and health (CRD42019137993)

A total of 31,063 titles were assessed for eligibility, and 28,682 were ineligible (Figure 1). Of 2381 full‐text articles assessed, 44 studies were included in the systematic review.

Table 1 summarizes the main characteristics of the included studies; extracted outcome data are shown in Tables S1–S6). Information about the policies evaluated by included studies is provided in Table S7.

**TABLE 1:** Key characteristics of *n* = 44 included studies

#	Citation details	Policy details				Study details	Outcomes reported						
	Lead author and year of publication	Country or region of policy	Policy evaluated	Year of implementation	Policy type	Study design	Medium^a^	Exp.	Power	Sales	Diet	UC	PC
1	Adams et al.^24^	UK	UK content and scheduling (Ofcom) restrictions	Phased 2007–2009	Mandatory	Repeated CS survey	TV	✓					
2	Berning & McCullough^25^	US	Childrens Food and Beverage Advertising Initiative (CFBAI)	2007	Voluntary	Repeated CS survey	TV	✓					
3	Brindal et al.^51^	Australia	Australian Food and Grocery Councils (AFGC) Responsible Marketing to Children Initiative (RCMI)	2009	Voluntary	Repeated CS survey	TV	✓	✓				
4	Campos et al.^26^	Spain	European and Spanish Public Health laws	2011	Mandatory	Repeated CS content analysis	TV	✓					
5	Clark^47^	Canada	Quebec Consumer Protection Act	1980	Mandatory	CS survey	N/A						✓
6	Dembek et al.^24^	US	Childrens Food and Beverage Advertising Initiative (CFBAI)	2007	Voluntary	Repeated CS survey	TV	✓					
7	Dillman Carpentier et al.^28^	Chile	Chile Food Labelling and Advertising Regulation	2016	Mandatory	Repeated CS content analysis and survey	TV	✓					
8	Dhar & Baylis^48^	Canada	Quebec Consumer Protection Act	1980	Mandatory	Natural experiment	N/A			✓			
9	Effertz & Wilcke^29^	EU	EU Pledge	2007	Voluntary	Repeated CS content analysis	TV	✓	✓				
10	Frazier & Harris^30^	US	Childrens Food and Beverage Advertising Initiative (CFBAI)	2007	Voluntary	Repeated CS survey	TV	✓					
11	Galloway & Calvert^52^	US	Childrens Food and Beverage Advertising Initiative (CFBAI)	2007	Voluntary	Repeated CS content analysis	Packaging	✓	✓				
12	Harris et al.^55^	US	Childrens Food and Beverage Advertising Initiative (CFBAI)	2007	Voluntary	CS survey	TV	✓	✓				
13	Harris et al.^53^	US	Childrens Food and Beverage Advertising Initiative (CFBAI)	2007	Voluntary	CS survey	TV	✓					
14	Harris & Kalnova^54^	US	Childrens Food and Beverage Advertising Initiative (CFBAI)	2007	Voluntary	CS survey	TV	✓					
15	Hebden et al.^63^	Australia	Australian Quick Service Restaurant Industry Initiative for Responsible Advertising and Marketing to Children (QSRI)	2009	Voluntary	Repeated CS content analysis and survey	TV	✓					
16	Huang & Yang^31^	US	Childrens Food and Beverage Advertising Initiative (CFBAI)	2007	Voluntary	Repeated CS survey	TV	✓		✓			
17	Kim et al.^32^	South Korea	Special Act on Safety Management of Childrens Dietary Life	2010	Mandatory	Repeated CS content analysis	TV	✓				✓	
18	King et al.^65^	Australia	Australian Food and Grocery Councils (AFGC) Responsible Marketing to Children Initiative (RCMI)	2009	Voluntary	Repeated CS content analysis	TV	✓	✓				
19	King et al.^64^	Australia	Australian Food and Grocery Councils (AFGC) Responsible Marketing to Children Initiative (RCMI) Australian Quick Service Restaurant Industry Initiative for Responsible Advertising and Marketing to Children (QSRI)	2009	Voluntary	Repeated CS content analysis	TV	✓					
20	Kunkel et al.^66^	US	Childrens Food and Beverage Advertising Initiative (CFBAI)	2006	Voluntary	Repeated CS content analysis	TV	✓	✓				
21	Landwehr & Hartmann^56^	Germany	EU Pledge	2007	Voluntary	CS content analysis	TV	✓					
22	Lwin et al.^33^	Singapore	Singapore Code of Advertising Practice (SCAP)	2015	Voluntary	Repeated CS content analysis and survey	TV	✓		✓	✓		
23	Mediano et al.^39^	Chile	Chile Food Labeling and Advertising Regulation	2016	Mandatory	Repeated CS content analysis	Packaging	✓	✓				
24	Morton et al.^34^	Australia	Australian Broadcasting Authoritys Childrens Television Standards	2009	Mandatory	CS content analysis	TV	✓	✓				
25	Neyens & Smits^57^	EU	EU Pledge	2007	Voluntary	CS content analysis	Websites	✓	✓				
26	Ofcom^35^	UK	UK content and scheduling (Ofcom) restrictions	Phased 2007–2009	Mandatory	Repeated CS survey	TV	✓	✓			✓	
27	Ofcom^36^	UK	UK content and scheduling (Ofcom) restrictions	Phased 2007–2009	Mandatory	Repeated CS survey	TV	✓	✓				
28	Otten et al.^43^	US	San Francisco Healthy Food Incentives Ordinance	2011	Mandatory	Repeated CS survey	Packaging			✓			
29	Potvin Kent et al.^45^	Canada	Quebec Consumer Protection Act	1980	Mandatory	Repeated CS content analysis and survey	TV	✓	✓				
30	Potvin Kent et al.^49^	Canada	Canadian Childrens Food and Beverage Advertising Initiative (CAI)	2008	Voluntary	CS content analysis and survey	TV	✓	✓				
31	Potvin Kent et al.^46^	Canada	Quebec Consumer Protection Act	1980	Mandatory	CS content analysis and survey	TV	✓					
32	Potvin Kent et al.^67^	Canada	Quebec Consumer Protection Act	1980	Mandatory	CS content analysis	Websites		✓				
33	Potvin Kent et al.^42^	Canada	Canadian Childrens Food and Beverage Advertising Initiative (CAI)	2008	Voluntary	Repeated CS survey	TV	✓	✓				
34	Potvin Kent & Pauze^50^	Canada	Canadian Childrens Food and Beverage Advertising Initiative (CAI)	2008	Voluntary	CS content analysis	Websites	✓					
35	Potvin Kent et al.^61^	Canada	Canadian Childrens Food and Beverage Advertising Initiative (CAI)	2008	Voluntary	CS survey	TV	✓					
36	Powell et al.^38^	US	Childrens Food and Beverage Advertising Initiative (CFBAI)	2006	Voluntary	Repeated CS survey	TV	✓					
37	Powell et al.^37^	US	Childrens Food and Beverage Advertising Initiative (CFBAI)	2006	Voluntary	Repeated CS survey	TV	✓					
38	Powell et al.^58^	US	Childrens Food and Beverage Advertising Initiative (CFBAI)	2006	Voluntary	CS survey	TV	✓					
39	Silva et al.^44^	UK	UK content and scheduling (Ofcom) restrictions	Phased 2007–2009	Mandatory	Repeated CS survey	N/A			✓		✓	
40	Théodore et al.^59^	Mexico	Mexican Self‐regulation	2009	Voluntary	CS content analysis	TV	✓					
41	Vaala & Ritter^62^	US	Childrens Food and Beverage Advertising Initiative (CFBAI)	2006	Voluntary	CS survey	Packaging		✓				✓
42	Vergeer et al.^60^	Canada	Canadian Childrens Food and Beverage Advertising Initiative (CAI)	2008	Voluntary	CS content analysis	Websites	✓	✓				
43	Warren et al.^40^	US	Childrens Food and Beverage Advertising Initiative (CFBAI)	2006	Voluntary	Repeated CS content analysis	TV	✓	✓				
44	Whalen et al.^41^	UK	UK content and scheduling (Ofcom) restrictions	Phased 2007–2009	Mandatory	Repeated CS content analysis	TV	✓					

*Note*: Mandatory—government‐enforced policies, Voluntary—voluntary measures.

Abbreviations: CS, cross‐sectional; Exp., Exposure; PC, product change; Sales, purchasing/sales; UC, unintended consequences.

^a^ Medium assessed is only applicable for exposure and power outcomes (it is not applicable for behavioral outcomes, unintended consequences, or product change).

All included studies were observational, and they examined changes (i) before and after implementation of policies (*n* = 21 studies^24^, ^25^, ^26^, ^27^, ^28^, ^29^, ^30^, ^31^, ^32^, ^33^, ^34^, ^35^, ^36^, ^37^, ^38^, ^39^, ^40^, ^41^, ^42^, ^43^, ^44^) or differences in outcomes between (ii) jurisdictions with and without restrictions or with different types of restrictions in place, including those comparing groups more or less likely to be exposed to the effects of the regulation, for example, English‐speaking and French‐speaking households in Quebec (*n* = 4 studies^45^, ^46^, ^47^, ^48^) or (iii) companies who were signatories versus non‐signatories to voluntary measures (*n* = 14 studies^49^, ^50^, ^51^, ^52^, ^53^, ^54^, ^55^, ^56^, ^57^, ^58^, ^59^, ^60^, ^61^, ^62^) or a combination of (i) and (iii) (*n* = 4 studies^63^, ^64^, ^65^, ^66^) or a combination of (ii) and (iii) (*n* = 1 study^67^). No studies were explicitly funded by the food industry.

Most studies (*n* = 37) did not involve human participants. The samples in these studies were broadcast television recordings, commercial datasets of television advertising, website observations, and commercial data related to food brands or products (e.g., price or availability or promotion in retail environments). Seven studies included human participants; the sample size in these studies ranged from 156 individual participants to 6000 households (including children).

Almost all (*n* = 43) studies evaluated food marketing policies in high‐income countries, namely, the United States (*n* = 15 studies^25^, ^27^, ^30^, ^31^, ^37^, ^38^, ^40^, ^43^, ^52^, ^53^, ^54^, ^55^, ^58^, ^62^, ^66^), Canada (*n* = 10 studies^42^, ^45^, ^46^, ^47^, ^48^, ^49^, ^50^, ^60^, ^61^, ^67^), the United Kingdom (*n* = 5 studies^24^, ^35^, ^36^, ^41^, ^44^), Australia (*n* = 5 studies^34^, ^51^, ^63^, ^64^, ^65^), the European Union (*n* = 3 studies^29^, ^56^, ^57^), Chile (*n* = 2 studies^28^, ^39^), Spain (*n* = 1 study^26^), Republic of Korea (*n* = 1 study^32^), and Singapore (*n* = 1 study^33^). One study was conducted in a middle‐income country (Mexico^59^).

Most (*n* = 33) studies evaluated effects of food marketing policies on television food advertising, with a small number of studies (*n* = 4) reporting on digital marketing (all websites) or product packaging (*n* = 4). Three studies did not measure a specific advertising medium (the outcomes reported in these studies were household food expenditure or purchase frequency), but these studies were intended to evaluate the effects of policies that restricted food advertising exclusively on television^44^, ^47^ or through all commercial avenues including television.^48^

With respect to the critical outcomes, those reported were exposure to marketing (37 studies), power of marketing (18 studies), unhealthy food purchasing (5 studies), and dietary intake (1 study). None of the included studies reported on the critical outcomes of food preferences or food choice.

With respect to important outcomes, those reported were unintended consequences (3 studies) and product change (2 studies). None of the included studies reported on the important outcomes of product requests, dental caries/erosion, BMI/obesity, or diet‐related NCDs.

### 3.2 Interventions

All interventions were within a single category: policies to restrict children's exposure to food marketing and its persuasive power. To support the WHO guideline development process, additional comparisons were conducted (Table 2).

**TABLE 2:** Summary of interventions and comparisons (*n* = 44 studies)

Intervention	Comparisons	*n*	Study IDs
Policies to restrict food and non‐alcoholic beverage marketing	1. Any policy vs. no policy (Comparison 2 [*n* = 10] + Comparison 3 [*n* = 29])	39	7 CS survey: Clark,^47^ Harris et al.,^55^ Harris et al.,^53^ Harris and Kalnova,^54^ Potvin Kent et al.,^61^ Powell et al.,^67^ Vaala & Ritter^62^
			13 repeated CS survey: Adams et al.,^24^ Berning & McCullough,^25^ Brindal et al.,^51^ Dembek et al.,^27^ Frazier & Harris,^30^ Huang & Yang,^31^ Ofcom,^35^ Ofcom,^36^ Otten et al.,^43^ Potvin Kent et al.,^42^ Powell et al.,^38^ Powell et al.,^37^ Silva et al.^44^
			5 CS content analysis: Neyens & Smits,^57^ Potvin Kent et al.,^67^ Potvin Kent & Pauze,^50^ Théodore et al.,^59^ Vergeer et al.,^60^
			10 repeated CS content analysis: Campos et al.,^26^ Effertz & Wilcke,^29^ Galloway & Calvert,^52^ Kim et al.,^32^ King et al.,^65^ King et al.,^64^ Kunkel et al.,^66^ Landwehr & Hartmann,^56^ Mediano et al.,^39^ Warren et al.^40^
			1 CS content analysis and survey: Potvin Kent et al.^49^
			3 repeated CS content analysis and survey: Dillman Carpentier et al.,^28^ Hebden et al.,^63^ Lwin et al, 2020.
	2. Mandatory policy vs. no policy	10	1 CS survey: Clark^47^
			5 repeated CS survey: Adams et al.,^24^ Ofcom,^35^ Ofcom,^36^ Otten et al.,^43^ Silva et al.^44^
			3 repeated CS content analysis: Campos et al.^26^ Kim et al.,^32^ Mediano et al.^39^
			1 repeated CS content analysis and survey: Dillman Carpentier et al.^28^
	3. Voluntary measures vs. no voluntary measure	29	6 CS survey: Harris et al.,^55^ Harris et al.,^53^ Harris and Kalnova,^54^ Potvin Kent et al.,^61^ Powell et al.,^58^ Vaala & Ritter^62^
			8 repeated CS survey: Berning & McCullough,^25^ Brindal et al.,^51^ Dembek et al.,^27^ Frazier & Harris,^30^ Huang & Yang,^31^ Potvin Kent et al.,^42^ Powell et al.,^38^ Powell et al.^37^
			5 CS content analysis: Neyens & Smits,^57^ Potvin Kent et al.,^67^ Potvin Kent & Pauze^50^ Théodore et al.,^59^ Vergeer et al.^60^
			7 repeated CS content analysis: Effertz & Wilcke,^29^ Galloway & Calvert,^52^ King et al.,^65^ King et al.,^64^ Kunkel et al.,^66^ Landwehr & Hartmann,^56^ Warren et al.^40^
			1 CS content analysis and survey: Potvin Kent et al.^49^
			2 repeated CS content analysis and survey: Hebden et al.,^63^ Lwin et al.^33^
	4. Mandatory policy vs. voluntary measures	4	1 CS content analysis: Morton et al.^34^
			1 CS content analysis and survey: Potvin Kent et al.^46^
			1 repeated CS content analysis and survey: Potvin Kent et al.^45^
			1 natural experiment: Dhar & Baylis^48^
	5. Mandatory policy (full implementation) vs. mandatory policy (partial implementation)	1	1 repeated CS content analysis: Whalen et al.^41^

Abbreviation: CS, cross‐sectional.

The overall effect of the intervention (i.e., policy to restrict food marketing) on all available outcomes is synthesized in the GRADE Evidence Profile (Table 3) using the approach set out by Murad et al.,^68^ for rating certainty in the absence of a single estimate of effect (see Supporting Information) and reported as per GRADE guidelines.^69^

**TABLE 3:** GRADE evidence profile

Certainty assessment							Impact	Certainty	Importance
No of studies	Study design	Risk of bias	Inconsistency	Indirectness	Imprecision	Other considerations			
Exposure to marketing									
37	Observational studies	Not serious	Very serious^a^	Not serious	Not serious	None	Overall, the evidence is very uncertain about the effect of implementing any food marketing policies on exposure to food marketing.	⨁◯◯◯ Very low	Critical
Power of marketing									
18	Observational studies	Not serious	Very serious^b^	Not serious	Not serious	None	The evidence is very uncertain about the effect of food marketing policies on power of food marketing.	⨁◯◯◯ Very low	Critical
Purchasing/sales									
5	Observational studies	Not serious	Not serious	Not serious	Not serious	None	The evidence suggests that food marketing policies may result in a reduction in food purchasing.	⨁⨁◯◯ Low	Critical
Food choice/intended choice									
0							None of the studies reported this outcome.	‐	Critical
Diet (energy, total food and/or nutrient intake, nutritional quality)									
1	Observational studies	Serious^c^	Not serious	Not serious	Not serious	None	The evidence is very uncertain about the effect of food marketing policies on dietary intake.	⨁◯◯◯ Very low	Critical
Preferences									
0							None of the studies reported this outcome.	‐	Critical
Body weight/BMI									
0							None of the studies reported this outcome.	‐	Important
Product requests/intended requests									
0							None of the studies reported this outcome.	‐	Important
NCD risk									
0							None of the studies reported this outcome.	‐	Important
Dental caries/erosion									
0							None of the studies reported this outcome.	‐	Important
Product change									
2	Observational studies	Not serious	Serious^d^	Serious^e^	Serious^f^	None	The evidence is very uncertain about the effect of food marketing policies on product change.	⨁◯◯◯ Very low	Important
Unintended consequences									
3	Observational studies	Not serious	Not serious	Not serious	Not serious	None	The evidence suggests that food marketing policies may result in unintended consequences that are favorable to public health.	⨁⨁◯◯ Low	Important

*Note*: Comparison 1 is used as the example for reporting subgroup analyses for purposes of brevity given that all studies included in Comparison 1 are also included in either Comparison 2 or Comparison 3, and numbers of studies were too small in Comparisons 4 and 5 for subgroup analyses to be undertaken.

Abbreviations: BMI, body mass index; NCD, noncommunicable disease.

^a^ The direction of effect varied considerably across the included studies: 4 reported a clear effect favoring the intervention, 11 reported an unclear effect potentially favoring the intervention, 7 reported no effect, 11 reported an unclear effect potentially favoring the control, and 4 reported a clear effect favoring the control. We therefore judged the evidence for this outcome to have very serious inconsistency and downgraded the certainty of evidence once for inconsistency.

^b^ The direction of effect varied considerably across the included studies: 3 reported a clear effect favoring the intervention, 2 reported an unclear effect potentially favoring the intervention, 1 reported no effect, 6 reported an unclear effect potentially favoring the control, and 6 reported a clear effect favoring the control. We therefore judged the evidence for this outcome to have very serious inconsistency and downgraded the certainty of evidence once for inconsistency.

^c^ Based on one study of only moderate quality due to methodological limitations (comparability of samples, outcome assessment).

^d^ The effect varied across the two studies: 1 reported a clear effect favoring the control, 1 reported no effect.

^e^ One of two studies used an indirect measure of marketing policy impact (cereal price).

^f^ Based on just two studies, but one study included data on 17 brands in 6 provinces and the other included 66 cereal brands (so substantial number of data points overall), therefore, deemed “serious” rather than “very serious” imprecision.

**FIGURE 2:**
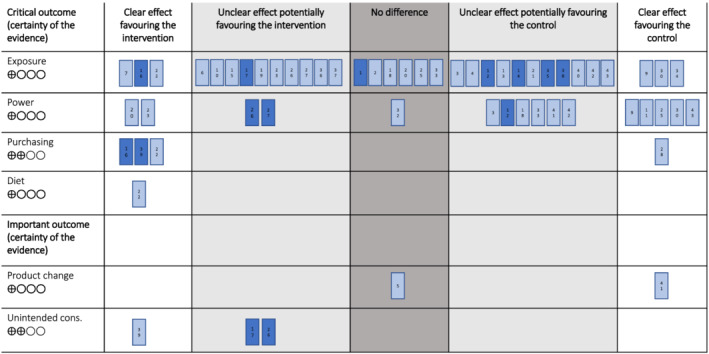
Harvest plot for Comparison 1 notes: • unintended cons. – unintended consequences • each bar represents one study • the number in each bar corresponds to the # number in Table 1 • dark blue shading indicates a high quality study • certainty of the evidence: ⊕◯◯◯ very low, ⊕⊕◯◯ low, ⊕⊕⊕◯ moderate, ⊕⊕⊕⊕ high

In this section, we present the results of the synthesis of the effects of the intervention on all critical and important outcomes for Comparison 1 (any policy vs. no policy). Evidence for Comparisons 2, 3, 4, and 5 is provided in the Supporting Information. In brief, Comparison 2 indicated that mandatory policies (versus no policy) were associated with a greater proportion of desirable effects than undesirable (five outcomes), whereas the opposite was found for voluntary measures (compared with no policy) in Comparison 3. Comparison 4 compared mandatory policies with voluntary measures directly and found that desirable effects were more likely with mandatory policies. Comparison 5 included a single study of a mandatory policy at full versus partial implementation and found a potentially desirable effect of full implementation on exposure to food marketing. The Harvest plots (Figures 2 and S1–S4) provide an overview of the effects for each comparison on all available outcomes. Subgroup analyses were possible for four policy design elements for Comparisons 1–3 (see Table 4 for details), and the results are reported in full in the supplement with a brief synopsis of subgroup results for Comparison 1 provided below.

**TABLE 4:** Summary of narrative subgroup analyses conducted

Outcome	Policy design element	Comparison 1	Comparison 2	Comparison 3
Exposure	Definition of a child in the policy	X		X
	Marketing medium	X	X	X
	Approach to classify foods	X	X	X
Power	Definition of a child in the policy	X		
	Marketing medium	X	X	X
	Approach to classify foods			
	Marketing techniques	X	X	X

### 3.3 Comparison 1: Any food marketing policy (voluntary/mandatory) versus no policy

Thirty‐nine studies reported effects of any food marketing policy (vs. no policy) on relevant outcomes of interest for this review. Studies in this comparison reported the effect of implementation of a mandatory policy compared with pre‐policy (prior to policy implementation; *n* = 9 studies^24^, ^26^, ^28^, ^32^, ^35^, ^36^, ^39^, ^43^, ^44^), the effect of implementation of a voluntary measure compared to before implementation of the measure (*n* = 10 studies^25^, ^27^, ^29^, ^30^, ^31^, ^33^, ^37^, ^38^, ^40^, ^42^), effects between companies who were signatories versus non‐signatories to voluntary measures (*n* = 15 studies^49^, ^50^, ^51^, ^52^, ^53^, ^54^, ^55^, ^56^, ^57^, ^58^, ^59^, ^60^, ^61^, ^62^, ^67^), or differences in outcomes between jurisdictions with and without mandatory restrictions (*n* = 1 study^47^). Four studies reported effect measures comparing both pre‐post voluntary measures and between signatories and non‐signatories.^63^, ^64^, ^65^, ^66^

#### 3.3.1 Critical outcome: Exposure to food marketing

Thirty‐three studies within this comparison arm reported on exposure.

Studies reporting a clear effect favoring the intervention all reported desirable effects on exposure (i.e., reductions) because of the policy. In a repeated cross‐sectional content analysis and survey design, Dillman Carpentier et al.^28^ reported significantly reduced weekly minutes of exposure to advertising for “high in” products (those exceeding thresholds for nutrients of public health concern) on TV channels popular with children in Chile post‐policy compared with pre‐policy. Huang et al.'s repeated cross‐sectional survey study^31^ reported significantly reduced gross rating points (GRPs, with 1 GRP being the equivalent of reaching 1% of the total potential audience with one advertisement) for confectionery (bubble gum) TV advertising post‐implementation of the Children's Food and Beverage Advertising Initiative (CFBAI) compared with pre‐implementation. Lwin et al.^33^ also used a repeated cross‐sectional content analysis and survey design and reported significantly reduced proportions of unique advertisements that were for unhealthy foods (based on predefined product categories) on the TV channels with highest viewership (including one dedicated to child and youth audiences) following implementation of the Singapore food marketing policy compared with pre‐implementation.

Ten studies reported an unclear effect potentially favoring the intervention; they also reported potentially desirable effects of any food marketing policy on exposure (narratively reported, no statistical testing). Dembek et al.^27^ and Frazier and Harris,^30^ both repeated cross‐sectional survey designs, found reductions in the average number of TV food advertisements viewed by children over a specified period (e.g., 1 year) pre‐ and post‐implementation of the CFBAI voluntary measure. Hebden et al.^63^ reported that a repeated cross‐sectional content analysis and survey design found a reduction in the mean frequency of non‐core fast food advertisements per year on the main free to air commercial TV channels pre‐ and post‐implementation of the Australian Quick Service Restaurant Industry Initiative. A repeated cross‐sectional content analysis found that GRPs for energy‐dense nutrient poor food TV advertisements during regulated hours reduced post‐implementation of the policy in the Republic of Korea relative to pre‐implementation.^32^ Other repeated cross‐sectional content analyses reported that the rate of non‐core food advertisements (not including fast food) per hour per TV channel on the main free to air commercial TV channels reduced post‐implementation of the Responsible Marketing to Children Initiative (RCMI) compared with pre‐implementation^64^ and the number and proportion of “high in” cereals in major supermarkets reduced after implementation of the Chilean regulations.^39^ The U.K. Office of Communication (Ofcom)'s repeated cross‐sectional surveys reported reduced impacts (a measure of advertisement viewing) of TV advertisements for HFSS foods in and around programming dedicated to children or “of particular appeal” to children post‐U.K. Government policy compared with pre‐policy.^35^, ^36^ In other studies, the mean number of TV food advertisements viewed by children per day reduced post‐CFBAI implementation^38^ and proportion of TV food advertisements viewed by children that were for unhealthy foods reduced, again post versus pre‐CFBAI.^37^

Six studies reported no effects of any food marketing policy, with effect measures of child person‐minute views (PMVs) of TV HFSS food advertising in a repeated cross‐sectional survey design,^24^ annual national GRPs for carbonated soft drink advertising also in a repeated cross‐sectional survey design,^25^ average number of non‐core food TV advertisements per hour on the main free‐to‐air commercial TV channels in a repeated cross‐sectional content analysis,^65^ unhealthy foods ads as a proportion of all food advertisements around children's TV programs on the most popular channels in repeated cross‐sectional content analysis and repeated cross‐sectional survey designs, respectively,^42^, ^66^ and average nutrition scores of websites of brands commonly marketed to children in a cross‐sectional content analysis.^57^

Eleven studies reported an unclear effect potentially favoring the control, so potentially undesirable effects on exposure. Brindal et al.^51^ reported that a repeated cross‐sectional survey found that RCMI signatory companies were responsible for a greater proportion of non‐core food advertisements as a percentage of all food advertisements on the main free to air TV channels compared with non‐signatories. Others reported a greater frequency of non‐core food advertisements per hour per channel on thematic channels for children following the implementation of public health laws on food marketing using a repeated cross‐sectional content analysis design.^26^ In three studies all using cross‐sectional survey designs, it was reported that CFBAI participating companies were responsible for a greater increase (percentage change over time) in number of confectionery advertisements viewed by children^55^ and greater volumes of food‐related advertisements viewed on children's TV^53^, ^54^ relative to nonparticipating companies. One cross‐sectional content analysis found a greater share of child‐targeted food advertising (on children's networks) by EU Pledge signatory companies compared with non‐signatories.^56^ A second cross‐sectional content analysis found a greater number and proportion of ultra‐processed foods advertised to children on highest rated TV channels by signatory companies of the Mexican self‐regulatory measure compared with non‐signatories.^59^ A third such content analysis found a greater proportion of products exceeding 15% Daily Value for saturated fats, sodium, and/or total sugars being marketed to children on websites from Canadian Children's Food and Beverage Advertising Initiative (CAI) signatory companies compared with non‐signatory companies.^60^ A repeated cross‐sectional content analysis found a greater frequency of TV food advertisements during likely child viewing hours post‐CFBAI implementation compared with pre‐implementation.^40^ One cross‐sectional survey found a greater number and proportion of food advertisements for less healthy items around TV programs with a child audience share ≥35% being from CAI signatory versus non‐signatory companies,^61^ and another cross‐sectional survey found a greater proportion of food ads that are “high in nutrients to limit” during children's programming by CFBAI signatories versus non‐signatories.^58^

Three studies (all assessing the effect of voluntary policy vs. no policy) clearly favored the control, reporting undesirable effects (i.e., increased exposure to food marketing) including a repeated cross‐sectional content analysis that found significantly increased proportions of non‐core food advertisements on TV channels popular with children and adolescents following implementation of the EU Pledge.^29^ Two Canadian studies evaluated the marketing by CAI signatory companies versus non‐signatories. One, using cross‐sectional content analysis and survey, found a significantly greater number and proportion of less healthy food advertisements by signatory companies during children's preferred television,^49^ and a second cross‐sectional content analysis found that the likelihood of food marketing being “less healthy” was significantly greater on the websites of signatory companies compared with non‐signatories.^50^

In summary, for the exposure to food marketing outcome, three studies clearly favored the intervention (13% [95% CI 0.3% to 34.7%], *p* < 0.001), 10 studies potentially favored the intervention (33.3% [95% CI 17.9% to 52.9%], *p* = 0.100), and 13 of 33 studies clearly or potentially favored the intervention (39.4% [95% CI 23.4% to 57.8%], *p* = 0.293). Eleven of 33 studies were judged to be high quality, and, of these, one clearly favored the intervention, and five potentially favored the intervention (see Figure 2). Certainty of evidence was deemed very low (Table 3).

##### Subgroup analyses for exposure (see Supporting Information for details)

For policies where children sought for protection were 12 years or under, seven (of 25) studies clearly or potentially favored the intervention. Where policies also sought to protect children over 12 years, six studies (of eight) clearly or potentially favored the intervention.

For policy effects on exposure to unhealthy food advertising on television, 12 studies (of 29) clearly or potentially favored the intervention; for digital marketing, no study (of three) clearly or potentially favored the intervention; and for product packaging, the single study identified potentially favored the intervention.

For policies using a nutrient profile model to classify restricted foods, two studies (of three) potentially favored the intervention (no studies clearly favored). For policies using company‐specific nutritional criteria, four studies (of 14) clearly or potentially favored the intervention. For policies using uniform category‐specific nutritional criteria, seven studies (of 14) clearly or potentially favored the intervention.

#### 3.3.2 Critical outcome: Power of food marketing

Sixteen studies within this comparison (any policy vs. no policy) reported on power outcomes.

Two studies reported a clear effect favoring the intervention, with desirable effects on power of food marketing (i.e., reductions) as a result of the policy. Both studies used repeated cross‐sectional content analysis designs. Kunkel et al.^66^ reported that a significantly lower proportion of TV advertisements for unhealthy foods (“whoa” foods, as a proportion of all food advertisements) featured a licensed character following implementation of the CFBAI (Table S7) compared with before implementation, and Mediano Stoltze et al.^39^ reported a significant reduction in the proportion of “high in” cereals available in supermarkets featuring at least one child‐directed marketing strategy (including child‐directed characters, gifts, games, toy or school references, child words or cross‐promotions with movies or TV shows) post‐policy in Chile (Table S7) compared with pre‐policy.

Two studies reported an unclear effect potentially favoring the intervention. They also reported potentially desirable effects of policies on exposure (narratively reported, no statistical testing). Both used repeated cross‐sectional survey designs but reported different effect measures. Ofcom^35^ reported a 56% reduction in the number of TV food advertising spots featuring licensed characters post‐policy (mid‐implementation) compared with pre‐policy, and Ofcom^36^ reported an 84% decrease in child impacts for TV food advertisements featuring a licensed character post‐policy (full implementation) compared with pre‐policy.

One study found no effect. Potvin Kent et al.'s^67^ cross‐sectional content analysis found no significant difference in their sample between the number and proportion of food brand websites with spokes characters between CAI signatory and non‐signatory companies.

Six studies (all assessing the impact of a voluntary policy vs. no policy) reported an unclear effect potentially favoring the control, that is, potentially undesirable effects on power as a result of policies, again with variation in study designs and effect measures. Brindal et al.^51^ reported on a repeated cross‐sectional survey that found that signatory companies for the RCMI were responsible for a greater number (and proportion) of non‐core TV food advertising using promotional characters than non‐signatory companies, and King et al.'s repeated cross‐sectional content analysis^65^ also found RCMI signatories to have broadcast a greater number and proportion of TV food advertisements using persuasive techniques compared with non‐signatories. One cross‐sectional survey found that CFBAI participating companies were responsible for a greater number (and proportion) of candy advertisements featuring child‐targeted techniques viewed by children compared with nonparticipating companies,^55^ and another cross‐sectional survey reported that the number of different child‐oriented features on high sugar cereal packaging was greater for CFBAI participating companies compared with nonparticipating companies.^62^ In a repeated cross‐sectional survey study, Potvin Kent et al.^42^ reported a 234% increase in the number of less healthy food TV advertisements following implementation of the CAI compared with pre‐implementation, and Vergeer et al.'s cross‐sectional content analysis^60^ found that 93.3% of CAI signatory companies had child‐directed marketing featuring marketing techniques on their website compared with just 6.7% of non‐signatories.

Five studies (all assessing the impact of a voluntary policy vs. no policy) clearly favored the control, reporting undesirable effects of the policy (i.e., increased power of food marketing). Effertz and Wilcke^29^ reported on a repeated cross‐sectional content analysis that found a significant increase in the propensity for non‐core food TV advertisements to contain a promotional character following implementation of the EU Pledge compared with pre‐implementation, and Neyens and Smits^57^ similarly reported a significantly greater presence of spokes characters on the websites of EU Pledge signatory companies compared with non‐signatories using a cross‐sectional content analysis design. Galloway and Calvert^52^ reported on a repeated cross‐sectional content analysis that found that compared with non‐signatories, CFBAI companies marketed more products with media characters on “Whoa” (nutritionally deficient) than “Go” (highest nutritional content) products and on “Whoa” than “Slow” (nutritionally beneficial but limit consumption) products. Potvin Kent et al.'s cross‐sectional content analysis and survey^49^ found that the number (and proportion) of TV food advertisements featuring media characters that were for less healthy foods was significantly higher for CAI signatory than non‐signatories, and Warren et al.^40^ found that the proportion of TV food advertisements featuring the production technique of animation was 10% higher following CFBAI implementation compared with pre‐implementation using a repeated cross‐sectional content analysis design.

In summary, for the power of marketing outcome, two studies clearly favored the intervention (14.3% [95% CI 2.5% to 43.9%], *p* = 0.016), two studies potentially favored the intervention (14.3% [95% CI 2.5% to 43.9%], *p* = 0.016), and four studies clearly or potentially favored the intervention (25.0% [95% CI 8.3% to 52.6%], *p* = 0.080). Three of 16 studies were judged to be high quality, and of these, none clearly favored the intervention and two potentially favored the intervention (see Figure 2). Certainty of evidence was deemed very low (Table 3).

##### Subgroup analyses for power (see Supporting Information for details)

For policies where children sought for protection were 12 years or under, one study (of 12) clearly or potentially favored the intervention. Where policies also sought to protect children over 12 years, three studies (of four) clearly or potentially favored the intervention.

For policy effects on power of unhealthy food advertising on television, three studies (of 10) clearly or potentially favored the intervention; for digital marketing, no study (of three) clearly or potentially favored the intervention; and for product packaging, one study (of three) clearly favored the intervention.

For studies evaluating policy effects on use of promotional characters in unhealthy food marketing, three studies (of 10) clearly or potentially favored the intervention. For child‐appealing persuasive strategies, one study (of five) clearly or potentially favored the intervention, and for the production technique of animation, the single study identified did not clearly or potentially favor the intervention.

#### 3.3.3 Critical outcomes: Food preferences and choice

None of the included studies reported these outcomes.

#### 3.3.4 Critical outcome: Food purchasing

Four studies within this comparison reported on purchasing outcomes.

A repeated cross‐sectional survey^31^ found that that relative purchase frequency for a confectionery item (bubble gum) decreased significantly in households with children pre‐post implementation of the CFBAI. Another repeated cross‐sectional survey found that in households with children, expenditure reduced by £6.2 per capita per quarter for HFSS foods and by £2.7 for HFSS drinks following implementation of the U.K. regulations, compared with pre‐implementation.^44^ One repeated cross‐sectional content analysis and survey found, via a home food inventory checklist, that post‐implementation (relative to pre‐) of the Singapore Code of Advertising Practice, the overall amount of unhealthy food in household pantries reduced significantly.^33^

One repeated cross‐sectional survey evaluating the San Francisco Healthy Foods Incentives Ordinance reported that the number and proportion of children's meals purchased increased significantly following implementation of the policy compared with pre‐implementation.^43^

In summary, for the food purchasing outcome, three studies clearly favored the intervention^31^, ^33^, ^44^ (75.0% [95% CI 21.9% to 98.7%], *p* = 0.62), no studies potentially favored the intervention. Three studies clearly or potentially favored the intervention (75.0% [95% CI 21.9% to 98.7%], *p* = 0.62). Two of four studies were judged to be high quality, and of these, both clearly favored the intervention.^31^, ^44^ Certainty of evidence was deemed low (Table 3).

#### 3.3.5 Critical outcome: Dietary intake

One study within this comparison reported on dietary intake outcomes.

This repeated cross‐sectional content analysis and survey reported that the self‐reported consumption score of children (9–16 years) for potato chips was significantly lower post‐implementation of the Singapore Code of Advertising Practice compared with pre‐implementation. An effect reported to be driven by the 13–16 year old's in the sample as no significant difference was found for those aged 9–12 years.^33^

In summary, for the dietary intake outcome, one moderate quality study clearly favored the intervention^33^ (100.0% [95% CI 5.5% to 100%], *p* = 1.00). Certainty of evidence was deemed very low (Table 3).

#### 3.3.6 Important outcomes: Product requests, dental caries/erosion, body weight/BMI/obesity, diet‐related NCDs

None of the included studies reported these outcomes.

#### 3.3.7 Important outcome: Product change

Two studies within this comparison reported on product change outcomes.

One cross‐sectional survey^62^ evaluated the sugar content of all ready‐to‐eat breakfast cereals (excluding granolas) available from two U.S. grocery stores between signatory and non‐signatory companies of the CFBAI, finding that mean sugar content per ounce was significantly higher for signatories.

Another cross‐sectional survey reported that there was no significant difference in the average price of children's brand breakfast cereals per 100 g between Canadian provinces with no regulation (analysis conducted pre‐CAI implementation) and Quebec, subject to the Quebec Consumer Protection Act (see Table S7).^47^

In summary, for the product change outcome, no studies clearly favored or potentially favored the intervention. One study was deemed to be high quality; this found no effect.^47^ Certainty of evidence was deemed very low (Table 3).

#### 3.3.8 Important outcome: Unintended consequences

Three studies within this comparison reported on unintended consequences.

One repeated cross‐sectional survey reported that there was a statistically significant reduction of £15.2 million in TV HFSS advertising expenditure following implementation of the U.K. regulations, compared with pre‐implementation.^44^

One repeated cross‐sectional content analysis of the mandatory policy from the Republic of Korea narratively reported that the total amount of money invested in TV advertising for energy‐dense nutrient poor food promotion during regulated hours fell pre‐post policy^32^ and one repeated cross‐sectional survey narratively reported that there was a 26% reduction in net food and drink advertising revenue on children's channels pre‐ to post‐U.K. Government policy implementation.^35^

In summary, for the unintended consequences outcome, one study clearly favored the intervention^44^ (100% [95% CI 5.5% to 100%], *p* = 1.00), and two studies potentially favored the intervention^32^, ^35^ (100% [95% CI 19.8% to 100%], *p* = 0.479). Three studies clearly or potentially favored the intervention (100% [95% CI 31.0% to 100%], *p* = 0.248); all were high quality. Certainty of evidence was deemed low (Table 3).

## 4 DISCUSSION

This review identified and synthesized evidence from 44 observational studies evaluating policies to restrict the food marketing to which children are exposed. Evidence suggests food marketing policies may result in reduced purchases of unhealthy foods and in unintended consequences favorable for public health. Desirable or potentially desirable (for public health) effects of policies on food marketing exposure and power were also found. Evidence on diet and product change was very limited. Overall, the certainty of the evidence was very low for four of the six outcomes (exposure, power, dietary intake, and product change) for which data were available and low for the two remaining outcomes (purchasing and unintended consequences).

The use of GRADE in this context has the potential to underestimate the certainty of evidence. The relatively low ratings reflect not just the inconsistency of effects (study heterogeneity) and, for some outcomes, risk of bias or considerations of indirectness and imprecision but also the nature of GRADE criteria. GRADE prioritizes randomized controlled trial data with clinical outcomes, observational studies have a lower starting position in assessments, and there is a requirement for certainty to be downgraded where there is unexplained heterogeneity even where results are consistent with previous reviews as is the case here. These issues have been described previously.^70^

According to GRADE, the evidence in this review is very uncertain about the effect of food marketing policies on children's exposure to food marketing. Certainty was downgraded due to very serious inconsistency in effects. This finding is consistent with previous reviews,^15^, ^16^, ^71^ all of which have noted similar patterns and attributed this heterogeneity, at least in part, to methodological differences between studies. One such difference is in the study design, for example, whether studies examined changes in marketing before and after implementation of the policies or whether differences were examined between companies who were or were not signatories of a voluntary measure. This is exemplified by the differences in effects reported by Powell et al., across three studies included in this review.^37^, ^38^, ^58^ Powell et al.^38^ and Powell et al.^37^ both used repeated cross‐sectional survey designs (pre‐post implementation) and reported potentially desirable effects of the CFBAI on children's food marketing exposure. Powell et al.^58^ was a cross‐sectional survey comparing marketing activity between signatory and non‐signatory companies of the CFBAI and reported potentially undesirable effects of the policy on exposure. In the case of voluntary measures, there may be other differences between signatory and non‐signatory companies (e.g., size of business and product portfolio) that can influence marketing practices and may therefore confound any observed effects^72^, ^73^ such that studies comparing marketing activity pre‐post policy implementation may be more informative.

The observed heterogeneity also likely stems from the sampling approach used in studies, which can also reflect fundamental disagreements between different actors as to what the aims of food marketing regulation are or should be. For example, Ofcom,^35^ Ofcom,^36^ Adams et al.,^24^ and Whalen et al.^41^ all evaluate the effectiveness of the U.K. Government's television food advertising policy on exposure outcomes but report different effects. The Ofcom evaluations focused on assessing change in advertising *directed to* children (operationalized as that appearing on dedicated children's channels or in and around programming “of particular appeal” to children based on audience share) as this is what the policy's stated aim is to restrict. Conversely, Adams et al.^24^ and Whalen et al.^41^ argue that the goal of regulation should be to reduce children's *overall* exposure to food advertising regardless of who that advertising is directed towards, a view consistent with the WHO Set of Recommendations^13^ and the WHO Framework,^12^ and thus, these studies explored change in marketing on all commercial channels (Adams et al.^24^) or programming *popular with* children whether or not child audience share met a particular threshold (Whalen et al.^41^). While Whalen et al. reported unclear effects potentially favoring the policy intervention similar to the two Ofcom evaluations, Adams et al. reported no effect, noting that while the policy was effective in excluding HFSS food advertising from the specific broadcast slots to which they apply, the policy did not achieve its stated aim to “reduce significantly the exposure of children under 16 to HFSS advertising” because HFSS food advertising migrated to adult airtime where it was watched by children in greater numbers than are engaged with child‐dedicated programming.^35^ These issues are acknowledged by Ofcom where it is noted that there was a growth in HFSS food advertising spots in adult airtime due to restrictions in child airtime, as well as a growth in the number of commercial channels available during the assessed period,^36^ which may explain the effects observed in the academic studies.^24^, ^41^ Therefore, it may be beneficial for future studies evaluating the efficacy of food marketing policies at reducing exposure to unhealthy food marketing to consider study designs and sampling that assess changes in children's exposure to food marketing that falls (i) within and (ii) outside of the scope of the policy, as well as children's overall exposure via the regulated medium and other marketing avenues. Reductions in (i) but not (ii) or (iii) would provide insight for policymakers that although the policy was effective in what it intended to do, it may not be sufficiently comprehensive in scope to meet public health goals, both in terms of the coverage of the regulated medium (e.g., programming coverage or regulated hours may be inappropriate or insufficient) and with consideration of the potential for displacement of marketing from regulated to less regulated media.

Another source of heterogeneity is the variation in effect measures used. This also in part reflects differences in sources of data, specifically whether studies used syndicated data purchased from a media research company or whether data were collected manually by researchers via participant survey or content analysis. Effect measures used to measure food marketing exposure by the studies included in this review include the number of food advertisements (count data), rate of food advertisements (per hour all channels/per hour per channel), the proportion of all advertisements that were for food, the proportion of food advertisements that were for unhealthy food products, the proportion of all advertisements (not just food) that were for unhealthy food products, nutritional quality of foods advertised, child “impacts,” GRPs, PMVs, and percentage change in one or more of these effect measures over time. Each type of effect measure has strengths and weaknesses in relation to others; for example, PMVs take into account that different advertisements have different lengths and are watched by different individuals so may be considered to be a better measure of exposure than count data,^24^ whereas five “impacts” could be five children seeing the same single advert once or one child seeing it five times—each of which may have differential effects on behavioral outcomes. The concept of “unhealthy food” is also defined differently across studies including core/non‐core (e.g., Whalen et al. and Hebden et al.^41^, ^63^), energy‐dense, nutrient poor (e.g., Kim et al.^32^), “high in” (e.g., Dillman Carpentier et al.^28^), HFSS (e.g., Ofcom^35^, ^36^), which is sometimes not the same as the way in which foods are classified as unhealthy in the policy (e.g., Whalen et al.^41^ use the core/non‐core classification in the evaluation of a policy that uses a nutrient profile model to define HFSS foods). More generally, it is important to note that the synthesized effect measures vary in terms of the extent to which they capture differences in exposure by the specific population targeted by the policies, for example, advertising GRPs for “children's advertising” during regulated timeslots (e.g., Kim et al.^32^), syndicated data for certain age groups (e.g., Dembek et al.^27^), or advertising on popular channels or during peak child viewing times, such that child exposure is inferred rather than measured directly (e.g., King et al.^65^).

According to GRADE, the evidence in this review is very uncertain about the effect of food marketing policies on power of food marketing. This heterogeneity also reflects the differences in study design and sampling discussed above for the exposure outcome and the selection of individual effect measures per outcome for synthesis in this review. Similar to the evidence discussed above for exposure, specifically the migration of advertising to adult airtime (which may be considered a “spill over” effect of the policy), Ofcom^35^ and Ofcom^36^ both reported that while the use of promotional characters in TV food advertising reduced post‐policy compared with pre‐policy, the use of celebrities increased in parallel (by more than 100%). Therefore, the specific “power” component(s) selected for inclusion in individual studies and syntheses (such as the current review) could affect whether a policy was analyzed as effective or not. There was also some limited evidence that the level of implementation of policies can affect policy efficacy. Ofcom^35^ was conducted when the U.K. policy was partially implemented, and Ofcom^36^ was conducted upon full implementation of the same policy. Both studies reported unclear effects on exposure and power potentially favoring the intervention, but, as would be expected, the magnitude of the effect was greater at full implementation (52% decrease in exposure effect, 84% decrease in power effect) than at partial implementation (39% decrease in exposure effect, 56% decrease in power effect).

In terms of policy design elements, subgroup analyses indicated that studies were more likely to report effects favoring the intervention when evaluating mandatory policies, policies designed to restrict food marketing to children including those >12 years, policies addressing television advertising, and policies using a nutrient profile model as the basis with which to classify foods to be restricted. Studies were also more likely to report desirable effects of policies on the use of promotional characters than on other marketing techniques. There is some overlap between these policy components—of the eight studies evaluating policies in which the age of a child included those over 12 years old, seven were reporting on mandatory policies,^24^, ^26^, ^28^, ^32^, ^35^, ^36^, ^39^ and three reported on a mandatory policy using a nutrient profile model.^24^, ^35^, ^36^ However, the binomial tests demonstrated that there was a significantly greater proportion of studies evaluating voluntary measures that showed undesirable effects for public health than showed desirable effects, whereas this was not the case for mandatory policies.

According to GRADE, the evidence in this review suggests that food marketing policies may result in a reduction in unhealthy food purchasing. The finding that a majority of studies including this outcome reported desirable effects is consistent with the results of a previous review of Euromonitor data in 2018,^17^ which evaluated the impact of “junk food” broadcast marketing policies on nationwide junk food sales. It was found that countries with government policies, but not self‐regulation, saw a significant decrease in sales per capita. Subgroup analyses were not possible in the current review due to the small number of studies, but Kovic and colleagues^17^ note that certain policy characteristics were associated with a greater decrease in junk food sales per capita, specifically standardized nutrition criteria (versus guidance nutrition criteria or none) and messaging requirements (vs. no requirements) and that these characteristics are typically found within mandatory policies to a greater extent than voluntary measures.

According to GRADE, the evidence in this review is very uncertain about the effect of food marketing policies on dietary intake. Only one study reported on this outcome, specifically a significant reduction in self‐reported potato chip consumption post policy,^33^ so there is a high risk of bias affecting certainty. Dietary intake is commonly reported in studies evaluating impact of food marketing,^5^, ^6^, ^7^, ^72^, ^74^ but studies reporting on the effect of policies on dietary intake outcomes are scarce, probably because in such a study, it would be extremely challenging to disentangle the effects of any policy from concurrent secular trends.^15^, ^75^ Given this dearth of evidence, it is likely necessary to defer to the evidence of policy impact on unhealthy food purchasing as representing changes in food‐based consumer behavior as this the closest outcome to intake (and typically involves much larger sample sizes than would be possible for studies of individual consumption effects). Household food purchase has been shown to yield a reasonably accurate estimate of overall diet quality but not dietary intake of specific nutrients.^76^

According to GRADE, the evidence in this review is very uncertain about the effect of food marketing policies on product change given the inconsistency, indirectness, and imprecision of the data. Only two studies reported on this outcome; one reported a clear effect favoring the control, as mean sugar content was significantly higher in breakfast cereals by signatory versus non‐signatory companies of the CFBAI^62^ and one study reported no effect of the Quebec Consumer Protection Act on the average price of children's breakfast cereals.^47^ There is evidence that other forms of public health policy (e.g., a soft drinks industry levy) drive product reformulation,^77^ and the use of nutrient thresholds within policies is considered a strategy for incentivizing reformulation,^15^, ^35^ but there is a lack of evidence to demonstrate this effect for food marketing policies to date.

According to GRADE, the evidence in this review suggests that food marketing policies may result in unintended consequences that are favorable to public health. All three studies that examined unintended consequences indicated positive changes in food advertising budgets or expenditure as a result of restrictive policies. However, caution is to be used when interpreting changes in advertising expenditure as trends observed can result from reduced advertising costs (e.g., due to increased numbers of commercial television channels and therefore greater availability of advertising spots) rather than a change in the amount of advertising activity taking place.^35^, ^36^

None of the included studies reported on food preferences, food choice, product requests, dental caries/erosion, body weight/BMI/obesity, or diet‐related NCD outcomes. As with dietary intake, it is challenging for studies to disentangle effects of policies on these outcomes from effects of secular trends. Modeling, or data simulation, studies were not eligible for inclusion in the current review, but recent studies using this approach have indicated that food marketing policies may significantly reduce obesity in children (via reduced consumption of unhealthy food products).^78^, ^79^ We could not conduct formal subgroup analyses by socioeconomic status, age, gender, or rural/urban residential status of participants or country income because enough studies did not (i) separate data by these variables in similar ways and (ii) provide/conduct analysis based on these variables.

Although focused comparisons and subgroup analyses (including those reported in the supplement) have allowed us to draw some conclusions about which policy components appear important for policy efficacy, more research will be needed to enable an in‐depth analysis. However, there are several key policy design components that may affect the effectiveness of policies to meet regulatory goals, namely, the age range of children sought for protection and how marketing to this age group is defined, which media are restricted, which forms of marketing are restricted, and how the specific foods and beverages to be restricted are classified. These issues have been discussed extensively in previous reviews of policies and their implementation (e.g., WHO Europe and Taillie et al.^14^, ^15^), but it is important to note that the evidence presented in the current review suggests that if a Government was to fully implement and enforce a comprehensive mandatory policy fully aligned with the spirit of the WHO recommendations, then it is likely that the policy would be effective as assessed against key indicators.^12^

### 4.1 Strengths and limitations

The strengths of this work are that it updates and builds upon the existing literature by providing a systematic global evidence review of the effectiveness of policies to restrict unhealthy food marketing to children, extending on previous more focused reviews by considering both mandatory policies and voluntary measures, a range of behavioral and health outcomes, and applying both quantitative synthesis approaches (including multiple focused comparisons) and evaluations of evidence certainty. The evidence reviewed has several limitations, most notably the lack of (i) studies from low‐ and middle‐income countries, (ii) longer‐term outcomes, (iii) detail on policies (design, implementation, enforcement), (iv) assessment across multiple media or other forms of marketing, (v) statistical analysis or reporting, and the heterogeneity in study design and effect measures. Regarding the latter, adoption of standardized monitoring procedures, including those proposed by WHO Europe^80^ and the International Network for Food and Obesity/noncommunicable disease Research, Monitoring and Action Support (INFORMAS)^81^ would be beneficial in facilitating the acquisition of robust, internationally comparative data. It should be noted with respect to long‐term outcomes such as body weight that policy evaluations tend to be observational and cross‐sectional in nature (as is reflected in this review), and therefore, it is difficult to separate the specific effect of a policy from that of an ongoing secular trend. Some studies (e.g., evaluations of the Quebec Consumer Protection Act) took place years after the implementation of the policy (in 1980) so were cross‐sectional comparisons (i.e., comparing groups more or less likely to be exposed to the effects of the regulation, e.g., English‐speaking and French‐speaking households in Quebec) rather than pre‐post implementation. Study designs may not be ideal in their ability to provide the evidence that policymakers are looking for because researchers are responding to real‐life events, often with limited time to collect pre‐policy data and without the facility to randomize to intervention conditions. The use of vote counting, though necessitated by the data available, is a limitation, as the selection of single effects per outcome per study limits the ability of a synthesis to address the complexity of the evidence. Also, this method allows a conclusion to be drawn as to whether there is any evidence of an effect but provides no information on the magnitude of effects and does not account for differences in the relative sizes of the studies.

## 5 CONCLUSION

Restrictions in food marketing may result in reduced purchases of unhealthy foods by or on behalf of children^31^, ^33^, ^44^, ^48^ and in unintended consequences favorable to public health.^32^, ^35^, ^44^ Several studies in this review also report desirable (or potentially desirable) effects of food marketing policies on child food marketing exposure^27^, ^28^, ^30^, ^31^, ^32^, ^33^, ^35^, ^36^, ^37^, ^38^, ^39^, ^41^, ^46^, ^63^, ^64^ and/or power.^35^, ^36^, ^39^, ^45^, ^66^ The certainty of evidence was very low for four outcomes (exposure, power, dietary intake, and product change) and low for two (purchasing, unintended consequences), largely due to inconsistency of effects (study heterogeneity). Mandatory policies, those seeking to protect children beyond 12 years of age, restricting unhealthy food advertising on television and using a nutrient profile model to classify foods were more likely to be evaluated as effective. Given that the impact of food marketing is a function of both exposure and power,^12^ and there is substantial evidence to demonstrate that food marketing is extensive, powerful, and impactful upon behavior,^8^, ^82^ it is positive for public health that these findings show it is possible for policies to effectively reduce the food marketing to which children are, or are likely to be, exposed and its persuasive power. The challenge for policymakers lies in designing and implementing policies with maximum potential for efficacy.

## CONFLICT OF INTEREST

We declare no competing interests.

## AUTHOR CONTRIBUTIONS

EB was responsible for the systematic review, wrote the manuscript, and was involved in the interpretation of results. AJ was responsible for the statistical analyses and was involved in interpretation of results. LM, MM, JH, and AB were involved with the systematic review and the interpretation of results. EB and LM accessed and verified the data. All authors were involved in devising and agreeing the final protocol for this work, had full access to all the data in the study, had final responsibility for the decision to submit for publication, reviewed and commented on the draft manuscript, and approved the submission of the final manuscript.

## Supporting information


**Table S1:** Exposure data
**Table S2:** Power data
**Table S3:** Purchasing data
**Table S4:** Dietary intake data
**Table S5:** Product change data
**Table S6:** Unintended consequences data
**Table S7.** Key characteristics of policies evaluated by included studies
**Figure S1.** Harvest plot for Comparison 2
**Figure S2.** Harvest plot for Comparison 3
**Figure S3.** Harvest plot for Comparison 4
**Figure S4.** Harvest plot for Comparison 5
