# Supplementary material for: Systematic review of the effect of policies to restrict the marketing of foods and non‐alcoholic beverages to which children are exposed
Source: Obes Rev. 2022 Apr 5;23(8):e13447. doi: 10.1111/obr.13447 (PMC9541016; doi:10.1111/obr.13447)
Supplement: Supplementary file 1 — Table S1: Exposure data Table S2: Power data Table S3: Purchasing data Table S4: Dietary intake data Table S5: Product change data Table S6: Unintended consequences data Table S7. Key characteristics of policies evaluated by included studies Figure S1. Harvest plot for Comparison 2 Figure S2. Harvest plot for Comparison 3 Figure S3. Harvest plot for Comparison 4 Figure S4. Harvest plot for Comparison 5 [file OBR-23-e13447-s001.docx]

**Systematic review of the effect of policies to restrict the marketing of foods and non-alcoholic beverages to which children are exposed**

Emma Boyland, Lauren McGale, Michelle Maden, Juliet Hounsome, Angela Boland, Andrew Jones

**Supplementary material table of contents**

**Appendix A:** Supporting information relating to the methodology 2

**Appendix B:** Selected data extraction by outcome 7

**Appendix C:** Policy information 24

**Appendix D:** Subgroup analyses for comparison 1 29

**Appendix E:** Comparison 2: Mandatory policy vs no policy 33

**Appendix F:** Comparison 3: Voluntary measures v no voluntary measure 39

**Appendix G:** Comparison 4: Mandatory policy vs voluntary measures 47

**Appendix H:** Comparison 5: Mandatory policy (full implementation) vs mandatory policy (partial implementation) 50

**Appendix A: Supporting information relating to the methodology**

***PICO question***

What is the effect in children on the **outcomes of interest** of implementing a policy that aims to restrict marketing of foods and non-alcoholic beverages to children **compared to not implementing the policy**?

***PICO table***

The PICO table was developed by NUGAG in Geneva in 2018 in accordance with the processes set out in the WHO Handbook for Guideline Development^1^.

| **PICO (MARKETING)** | **Considerations for key questions** |
| --- | --- |
| Populations | Children (0-19 years)   - By body weight - By socio-economic status (e.g. income, education, occupation) - By age groups, gender, urban/rural - By region (HIC, LMIC) |
| Intervention/ exposure | **Policies that restrict children’s exposure to and/or power of marketing foods and non-alcoholic beverages**  **Policy design elements:**   - Target population (age/definition of children) - Target marketing approaches - Target food & beverage (and approach to define target foods) - Legal instrument: voluntary and mandatory   **Policy implementation and enforcement:**   - Degree (and quality) |
| Comparison | No (or different) policies to restrict marketing to children |
| Outcomes (critical) | Exposure to marketing  Food choice  Diet-related NCDs (including validated surrogate indicators)  Power of marketing  Food purchasing/ sales  Food preference |
| Outcomes (important) | Body weight/body mass index/obesity  Pester power  Unintended consequences (to wider society e.g., revenue, jobs)  Product change (any change to available products e.g., portion size, food reformulation, portfolio mix)  Risk of diet-related NCDs  Dental caries/ erosion |

***Example search strategy***

Search terms were approved by a WHO librarian prior to finalization. An example of a search strategy using keywords for MEDLINE is provided (adaptations were made as required for other databases):

Database: Ovid MEDLINE(R) ALL <1946 to March 14, 2019>

Search Strategy:

--------------------------------------------------------------------------------

1 exp Food/ or exp Food Industry/ or beverages/ or carbonated beverages/ or

energy drinks/ or "fruit and vegetable juices"/ or exp milk/ or exp milk substitutes/ or

exp Tea/ or exp Teas, Herbal/ (1301190)

2 (food* or diet* or snack* or nutrition* or fast-food* or beverage* or drink*).ti.

(400423)

3 Diet/ (148911)

4 1 or 2 or 3 (1563135)

5 Direct-to-Consumer Advertising/ or Advertising as Topic/ (14390)

6 exp Marketing/ (33768)

7 (advert* or advergame* or sponsor* or promot* or market* or adspend* or

commercial or commercials).ti,ab. (1177279)

8 5 or 6 or 7 (1192692)

9 4 and 8 (103428)

10 exp adolescent/ or exp child/ or exp child, preschool/ or exp infant/ or exp

schools/ (3453577)

11 (child* or adolescen* or infant* or youth* or "young people" or "young person"

or teen* or pupil*).ti,ab. (1755717)

12 10 or 11 (3890679)

13 9 and 12 (13590)

14 exp government regulation/ or exp mandatory programs/ or exp Health Policy/

or exp Taxes/ (133900)

15 (ban or bans or policy or policies or regulat* or legislat* or law or laws or statut*

or self-regulat* or co-regulat* or coregulat* or prohibit* or restrict* or tax* or

mandatory or voluntary or pledge* or charter or charters or legal or commitment* or

standard or standards or code or order or decree).ti,ab. (4353660)

16 14 or 15 (4428834)

17 13 and 16 (4689)

18 ((food* or diet* or snack* or nutrition* or fast-food* or beverage* or drink*) adj3

(advert* or advergame* or sponsor* or promot* or market* or adspend* or

commercial or commercials) adj3 (ban or bans or policy or policies or regulat* or

legislat* or law or laws or statut* or self-regulat* or co-regulat* or coregulat* or

prohibit* or restrict* or tax* or mandatory or voluntary or pledge* or charter or

charters or legal or commitment* or standard or standards or code or order or

decree)).ab. (694)

19 12 and 18 (305)

20 17 or 19 (4738)

***Selecting comparators***

Due to the nature of the available evidence, the comparator was not consistent for all studies. If the study evaluated the effectiveness of a voluntary measure, the comparison was either marketing activity pre-implementation of the measure or post-measure but by non-signatory companies (i.e., in both cases, this reflects the absence of a policy in the comparator arm). If studies evaluated the effectiveness of a current mandatory policy, the comparison was marketing activity under a previous policy (either a voluntary measure, or at the point of partial implementation of the mandatory policy) or that occurring prior to the implementation of the policy (pre-policy i.e., no policy). Where more than one eligible data point was available for pre-policy (comparator) and post-policy (intervention) arms, the comparator data was selected as the data point closest to the introduction of the policy (to maximize the comparability of the two points in terms of contemporary food environments) and the intervention data was selected as the most recent data point (to maximize the likelihood of policy-related impacts having manifested in our outcomes of interest).

***Grading the certainty of evidence***

The GRADE approach (Grading of Recommendations, Assessment, Development and Evaluation) was applied to assess the certainty of the available evidence for each outcome (GRADEpro software [https://gradepro.org/](https://gradepro.org/licences/)).

GRADE focuses on the internal validity of bodies of evidence and is widely used in guideline development. Certainty of evidence can be graded as very low, low, moderate, or high. The evidence from observational studies start as being of low quality, while the evidence from randomised controlled trials starts as high quality. We considered five criteria for lowering the level of confidence: risk of bias, indirectness, imprecision, inconsistency, and likelihood of publication bias. Further, the level of confidence could be raised by three criteria: large effect, dose-response gradient, and where the influence of all plausible confounding would reduce a demonstrated effect or suggest a spurious effect when results show no effect (dose-response and plausible confounding were only considered where the evidence had not been downgraded on any domain for any reason), but this did not occur.

We applied the constructs of GRADE in accordance with recommendations for rating the certainty of evidence in the absence of a single estimate of effect i.e., where data have been summarised narratively. Results are presented in s summary of findings table, and we provide rationales for judgements in footnotes beneath the Evidence profile table.

***Vote counting by direction of effect***

This synthesis method allows a conclusion to be drawn as to whether there is any evidence of an effect but provides no information on the magnitude of effects and does not account for differences in the relative sizes of the studies.

In accordance with Cochrane recommendations, we first determined which studies were similar enough to be grouped within each comparison (based on PICO elements; namely the primary policy approach and implementation level for the intervention) and for subgroups within comparisons (based on key policy characteristics).

We then explored multiplicity within the data (where multiple effects within the same outcome domain were reported e.g., if a study reported data on purchasing of a number of different food groups). As recommended by Cochrane for applying the vote counting based on the direction of effect synthesis method, we selected one effect per study per outcome to include in the synthesis. Due to the lack of effect estimates, decision rules were applied to select the most relevant effect. The effects selected for synthesis were thosejudged to be the most comprehensive measure of the outcome and most relevant for decision making (e.g., overall unhealthy food marketing instead of marketing of individual food groups). In Appendix B Tables S1-S6 we present the results of individual studies; the green shaded rows indicate the effects selected to inform the synthesis (GRADE evidence profile and harvest plots). Other effectsare reported in the text of the review where they add unique value to the policy evaluation (e.g., if there are separate effects to show the effect of a policy on food marketing exposure in children who are and aren’t considered “children” as defined in the policy, or where an additional effect measure indicates a different direction of effect to the synthesized effect measure) but not where they provide similar/equivalent data to that already provided by the measure included in the synthesis (e.g., where the effect measure in the synthesis showed a reduction in the *overall number* of unhealthy food advertisements shown on a television channel, and the additional measure showed a reduction in the *rate* of unhealthy food advertisements per hour on that channel).

We have therefore synthesized the data using vote counting based on effect direction (ED). Based on the guidance in the Cochrane Handbook (chapter 12, in particular 12.2.1.3 on vote counting^2^ and the SWiM project^3^, five categories of ED were used in the review:

1. *Clear effect favoring the intervention*, where the effect estimate favors the intervention and the 95% confidence interval (CI) excludes the null (▲);
2. *Unclear effect potentially favoring the intervention*, where the effect estimate favors intervention but the 95% CI includes the null and is wide △;
3. *No difference in effect*, where the 95% CI crosses the null but is narrow €;
4. *Unclear effect potentially favoring the control*, where the effect estimate favors the control but the 95% CI includes the null and is wide (▽); and
5. *Clear effect favoring the control*, where the effect estimate favors the control and the 95% CI excludes the null (▼).

If a 95% CI was not reported, the p-value was used to determine whether the direction of effect was clear or unclear (or if no difference existed, taken as p>0.05) but not to determine the direction of effect. If no effect estimates or p-value were reported, effects were always classified as ‘unclear’. Author reports of effect direction (e.g., if authors stated that one value was significantly greater than the other) and/or statistical significance (e.g., if no p-value was reported but authors stated that no significant difference had been identified) were used to guide decisions.

We applied the binomial probability test on the (i) number of effects favoring the intervention and (ii) number of effects potentially favoring the intervention each compared to the number of effects clearly favoring the control, potentially favoring the control, or showing no effect. Analyses were conducted using the ‘prop.test’ function in R. The null hypothesis tested is that proportion= .50 (i.e., that there is an equal probability of effects favoring the intervention versus not). Therefore, the two-sided p-value, based on Pearson’s chi-squared statistic, reflects the proportion difference between effects with desirable (clear or potential public health benefit) effects versus those with undesirable (clear or potential public health harm) effects or no effect. Significant p-values can represent either a significantly *smaller* proportion of desirable effects for public health or a significantly *larger* proportion of desirable effects for public health compared with effects in the other categories. A non-significant p-value is indicative of no significant differences in the proportions. Narrow CIs reflect more precise estimates of the proportion of interventions with desirable effects, due to increased number of studies in the analysis.

Appendix A

References

1. World Health Organization. WHO Handbook for Guideline Development (2nd Edition). Accessible from <https://apps.who.int/iris/bitstream/handle/10665/145714/9789241548960_eng.pdf?sequence=1&isAllowed=y>; 2014.

2. McKenzie JE, Brennan SE. Chapter 12: Synthesizing and presenting findings using other methods. In: Higgins JPT, Thomas J, Chandler J, et al., eds. Cochrane Handbook for Systematic Reviews of Interventions version 62 (updated February 2021) Available from wwwtrainingcochraneorg/handbook: Cochrane; 2021.

3. Campbell M, McKenzie JE, Sowdon A, et al. Synthesis without meta-analysis (SWiM) in systematic reviews: reporting guideline *British Medical Journal* 2020; **368**: I6890.

**Appendix B: Selected data extraction by outcome**

**Key:**

NOS – Newcastle-Ottawa Scale score (Quality Appraisal)

NR – not reported

Non-sig – non-significant

The green shaded rows indicate the outcomes selected to inform the synthesis (GRADE evidence profile and harvest plots).

**Table S1: Exposure data**

| **Study name** | **NOS** | **Measure of effect** | **Details of subgroup (if relevant)** | **Name of arm 1** | **Name of arm 2** | **Value arm 1** | **Value arm 2** | **Effect size** | **p value** | **Effect direction (synthesised data only)** |
| --- | --- | --- | --- | --- | --- | --- | --- | --- | --- | --- |
| Adams 2012 | 8 | PMV (% of all advertising) | Viewers 4-15y | Pre-policy | Post-policy | 11,989 (14.2) | 13,429 (12.6) | OR (99% CI) = 0.85 (0.82 - 0.89) | NR |  |
| Adams 2012 | 8 | PMV (% of all food advertising) | Viewers 4-15y | Pre-policy | Post-policy | 5174 (43.2) | 7476 (55.7) | OR (99% CI) = 1.25 (1.15 - 1.37) | NR |  |
| Adams 2012 | 8 | PMV (% of all advertising) | Viewers 4-15y | Pre-policy | Post-policy | 5174 (6.1) | 7476 (7.0) | OR (99% CI) = 1.05 (0.99 - 1.12) | NR | € |
| Berning 2013 | 6 | Annual National Advertising GRPs for children | Coca-Cola | Pre-policy | Post-policy | 3619 | 1199 | -6.552 | <0.01 |  |
| Berning 2013 | 6 | Annual National Advertising GRPs for children | Pepsi | Pre-policy | Post-policy | 2593 | 1430 | -5.682 | NR | € |
| Brindal 2011 | 5 | Non-core food ads as a % of all food ads |  | Non-signatories | Signatories | 23.5 | 78.3 | NR | NR | ▽ |
| Brindal 2011 | 5 | Non-core food ads as a % of all food ads |  | Non-signatories | Signatories | 92.8 | 95.3 | NR | NR |  |
| Brindal 2011 | 5 | Non-core food ads as a % of all food ads |  | Non-signatories | Signatories | 50.8 | 86.7 | NR | NR |  |
| Brindal 2011 | 5 | Non-core food ads as a % of all food ads |  | Non-signatories | Signatories | 9.1 | 26.9 | NR | NR |  |
| Campos 2016 | 6 | Total of frequency and ratio [n (advertisements/hour/channel)] | NA | Pre-policy | Post-policy | 216 (6) | 365 (10) | NR | NR |  |
| Campos 2016 | 6 | Frequency of non-core food advertisements and proportion (% of food ads) | Non-core food advertising | Pre-policy | Post-policy | 647 (66.6) | 302 (52.2) | NR | <0.05 | ▽ |
| Campos 2016 | 6 | Total of frequency and ratio [n (advertisements/hour/channel)] | Non-core food advertising | Pre-policy | Post-policy | 180 (5) | 203 (6) | NR | NR |  |
| Dembek 2012 | 6 | Average food and beverage ads viewed per year | Children (2-11y) | Pre-policy | Post-policy | 5078 | 4610 | 8.9% decrease | NR | △ |
| Dembek 2012 | 6 | Average food and beverage ads viewed per year | Adolescents (12-17y) | Pre-policy | Post-policy | 4790 | 5820 | 21.5% increase | NR |  |
| DillmanCarpentier 2020 | 4 | Weekly minutes of exposure, mean (SD) | Preschoolers | Pre-policy | Post-policy | 2.5 (3.2) | 1.4 (1.6) | F=86.0 unadjusted | <0.001 |  |
| DillmanCarpentier 2020 | 4 | Weekly minutes of exposure, mean (SD) | Adolescents | Pre-policy | Post-policy | 7.3 (6.6) | 3.1 (3.7) | F=246.5 unadjusted | <0.001 |  |
| DillmanCarpentier 2020 | 4 | Weekly minutes of exposure, mean (SD) | Preschoolers | Pre-policy | Post-policy | 2.0 (2.4) | 1.3 (1.4) | F=60.4 unadjusted | <0.001 | ▲ |
| DillmanCarpentier 2020 | 4 | Weekly minutes of exposure, mean (SD) | Adolescents | Pre-policy | Post-policy | 5.0 (4.4) | 2.4 (2.8) | F=184.5 unadjusted | <0.001 |  |
| Effertz 2012 | 5 | % of food advertisements (of all advertisements) | NA | Pre-policy | Post-policy | 14.5 | 18.5 | X2 = 20.624 | <0.001 |  |
| Effertz 2012 | 5 | % of non-core food advertisements (of all food advertisements) | Non-core food advertising | Pre-policy | Post-policy | 88.2 | 98.2 | X2 = 24.634 | <0.001 | ▼ |
| Frazier 2018 | 3 | Average number of food and beverage advertisements viewed | Children (2-11y) | Pre-policy | Post-policy | 5099 | 3665 | 28% decrease | NR | △ |
| Frazier 2018 | 3 | Average number of food and beverage advertisements viewed | Adolescents (12-17y) | Pre-policy | Post-policy | 4829 | 3449 | 29% decrease | NR |  |
| Harris 2015 | 7 | Change in number (% change) of candy advertisements viewed | Children (2-11y) | Non-participating brands | Participating companies, non-approved brands | 35 (41) | 190 (152) | NR | NR | ▽ |
| Harris 2017 | 5 | Totals ads viewed | Preschoolers (2-5y) | Non-signatories | Signatories | 527.6 | 775.4 | NR | NR |  |
| Harris 2017 | 5 | Totals ads viewed | Children (2-6y) | Non-signatories | Signatories | 531 | 957.1 | NR | NR |  |
| Harris 2017 | 5 | Totals ads viewed | Young teens (12-14y) | Non-signatories | Signatories | 466.1 | 560.7 | NR | NR |  |
| Harris 2017 | 5 | Totals ads viewed | Preschoolers (2-5y) | Non-signatories | Signatories | 290.3 | 675.8 | NR | NR |  |
| Harris 2017 | 5 | Totals ads viewed | Children (2-6y) | Non-signatories | Signatories | 270.3 | 838 | NR | NR | ▽ |
| Harris 2017 | 5 | Totals ads viewed | Young teens (12-14y) | Non-signatories | Signatories | 141.5 | 429.4 | NR | NR |  |
| Harris 2017 | 5 | Number of advertisement impressions (000) |  | Non-signatories | Signatories | 34768 | 473406 | NR | NR |  |
| Harris 2017 | 5 | Number of advertisement impressions (000) |  | Non-signatories | Signatories | 485507 | 2872807 | NR | NR |  |
| Harris 2017 | 5 | Number of advertisement impressions (000) |  | Non-signatories | Signatories | 454 | 183 | NR | NR |  |
| Harris 2017 | 5 | Number of advertisement impressions (000) |  | Non-signatories | Signatories | 2420 | 3204 | NR | NR |  |
| Harris 2018 | 7 | Average number of food advertisements viewed | Preschoolers (2-5y) | Non-participating companies | Participating companies | 422.3 | 732.2 | NR | NR |  |
| Harris 2018 | 7 | % of total food advertisements viewed | Preschoolers (2-5y) | Non-participating companies | Participating companies | 37 | 63 | NR | NR |  |
| Harris 2018 | 7 | Average number of food advertisements viewed | Older children (6-11y) | Non-participating companies | Participating companies | 402.8 | 910.0 | NR | NR | ▽ |
| Harris 2018 | 7 | % of total food advertisements viewed | Older children (6-11y) | Non-participating companies | Participating companies | 31 | 69 | NR | NR |  |
| Hebden 2011 | 6 | Mean frequency of non-core food advertisements per hour | Non-core food advertising | Pre-policy | Post-policy | 3.1 | 2.4 | IRR 0.74 (95% CI 0.65-0.83) | <0.001 |  |
| Hebden 2011 | 6 | Mean frequency of total fast food advertisements per hour | Fast food advertising | Pre-policy | Post-policy | 1.1 | 1.5 | IRR 1.26 (95% CI 1.06-1.51) | 0.008 |  |
| Hebden 2011 | 6 | Mean frequency of non-core fast food advertisements per hour | Non-core fast food advertising | Pre-policy | Post-policy | 1.0 | 1.0 | IRR 0.73 (95% CI 0.60-0.88) | 0.001 | △ |
| Hebden 2011 | 6 | Change in rate (%) of non-core fast food advertising | Non-core fast food advertising | Non-signatories | Signatories | -84 | -17 | IRR 5.18 (95%CI 2.10-12.79) | <0.001 |  |
| Huang 2013 | 8 | Bubble Gum GRP change (SD) | Children (2-11y) | Pre-policy | Post-policy | NR | NR | Decrease 45.924 (0.077) | <0.01 | ▲ |
| Kim 2013 | 8 | Number of advertisement insertions into advertising time slots | EDNP food advertising | Pre-policy | Post-policy | 295 | 6 | NR | NR |  |
| Kim 2013 | 8 | GRPs (estimate of audience size) | EDNP food advertising | Pre-policy | Post-policy | 183 | 0.8 | NR | NR | △ |
| King 2011 | 6 | Average number of food ads per hour (total food ads/hours recorded) |  | Pre-policy | Post-policy | 7.0 | 5.9 | IRR 0.65 (0.59-0.71) | <0.01 |  |
| King 2011 | 6 | Average number of non-core food ads per hour (total food ads/hours recorded) | Non-core food advertising | Pre-policy | Post-policy | 3.4 | 3.2 | IRR 1.05 (0.84-1.17) | NS | € |
| King 2011 | 6 | Proportion (%) of all advertisements that were for food (2007 v 2009) |  | Pre-policy | Post-policy | 26 | 16 | NR | NR |  |
| King 2011 | 6 | Change in proportion (%) of non-core food advertising | Non-core food advertising | Non-signatories | Signatories | 42 | -24 | IRR 0.53 (0.43-0.65) | <0.01 |  |
| King 2011 | 6 | Change in proportion (%) of non-core food advertising | Non-core food advertising | Non-signatories | Signatories | NR | NR | IRR 0.93 (0.69-1.26) | <0.01 |  |
| King 2011 | 6 | Average number of non-core food ads per hour (total food ads/hours recorded) | Non-core food advertising | Non-signatories | Signatories | 1.8 | 1.3 | Mean difference 0.35 | <0.01 |  |
| King 2011 | 6 | Average number of non-core food ads per hour (total food ads/hours recorded) | Non-core food advertising | Non-signatories | Signatories | 2 | 1.5 | NR | NR |  |
| King 2013 | 6 | Mean frequency of non-core food advertisements (excluding fast food) per hour per channel | Non-core food advertising (RCMI) | Pre-policy | Post-policy | 2.36 | 1.64 | NR | NR | △ |
| King 2013 | 6 | Mean frequency of non-core fast food advertisements per hour per channel | Non-core food advertising (QSRI) | Pre-policy | Post-policy | 1.35 | 1.51 | NR | NR |  |
| King 2013 | 6 | Proportion (%) of all the non-core food advertisements | Non-core food advertising | Non-signatories | Signatories | 38 | 62 | NR | NR |  |
| King 2013 | 6 | Proportion (%) of all the fast food advertisements | Non-core food advertising | Non-signatories | Signatories | 10 | 90 | NR | NR |  |
| Kunkel 2015 | 6 | Number of food ads/hour |  | Pre-policy | Post-policy | 8.5 | 6.4 | Z=-4.5 | <0.001 |  |
| Kunkel 2015 | 6 | Minutes per hour devoted to food advertisements (minutes: seconds) |  | Pre-policy | Post-policy | 03:29 | 02:21 | Z=-3.73 | <0.001 |  |
| Kunkel 2015 | 6 | Unhealthy (whoa) food advertisements as a proportion (%) of all food advertisements | Whoa food advertising | Pre-policy | Post-policy | 79.4 | 80.5 | Z=0.75 | 0.45 |  |
| Kunkel 2015 | 6 | Unhealthy (whoa) food advertisements as a proportion (%) of all food advertisements | Whoa food advertising | Pre-policy | Post-policy | 76.4 | 75.3 | NR | >0.05 | € |
| Kunkel 2015 | 6 | Number and proportion (%) of overall food advertisements |  | Non-signatories | Signatories | 107 (30.2) | 247 (69.8) | NR | NR |  |
| Kunkel 2015 | 6 | Unhealthy (whoa) food advertisements as a proportion (%) of all food advertisements | Whoa food advertising | Non-signatories | Signatories | 92.5 | 75.3 | Z=3.76 | <0.001 |  |
| Landwehr 2020 | 6 | Share of child-targeted food and beverage advertising (% in total commercials) |  | Non-signatories | Signatories | 1.6 | 2.2 | NR | NR |  |
| Landwehr 2020 | 6 | Share of child-targeted food and beverage advertising (% in children's networks) | Children's networks | Non-signatories | Signatories | 3 | 3.3 | NR | NR | ▽ |
| Lwin 2020 | 5 | Proportion of unique ads that were for unhealthy food products |  | Pre-policy | Post-policy | 55.1 | 37.65 | Z=1.96 | 0.03 | ▲ |
| Mediano 2019 | 6 | Number (%) of "high in" products as a proportion of all cereals |  | Pre-policy | Post-policy | 132 (78.6) | 86 (59) | NR | NR | △ |
| Morton 2005 | 5 | Number and proportion (%) of all advertisements that were for food |  | Self-regulation | Government | 447 (30.1) | 97 (41.1) | X2=11.4 | <0.001 | ▼ |
| Neyens 2017 | 5 | Average UK NPM nutrition score of products promoted on website (Mann-Whitney mean ranks) |  | Non-signatories | Signatories | 15 | 16.36 | U=114, z=0.41, r=0.075 | 0.703 | € |
| Ofcom 2008 | 7 | HFSS impacts (number of times an HFSS advertisement is seen) in billions (% of all food impacts that were HFSS) |  | Pre-policy | Post-policy (partial implementation) | 12.5 (83%) | 8.3 (64%) | 34% decrease | NR |  |
| Ofcom 2008 | 7 | HFSS impacts (number of times an HFSS advertisement is seen) in billions (% of all food impacts that were HFSS) | Children (4-9y) | Pre-policy | Post-policy (partial implementation) | 6.1 | 3.7 | 39% decrease | NR | △ |
| Ofcom 2008 | 7 | HFSS impacts (number of times an HFSS advertisement is seen) in billions (% of all food impacts that were HFSS) | Children (10-15y) | Pre-policy | Post-policy (partial implementation) | 6.4 | 4.6 | 28% decrease | NR |  |
| Ofcom 2010 | 7 | Number in millions (%) of HFSS advertising spots (as a % of all food) |  | Pre-policy | Post-policy | 1.7 (10) | 3.2 (9.8) | 85% increase in absolute number, little change in % | NR |  |
| Ofcom 2010 | 7 | Number in millions (%) of HFSS advertising spots (as a % of all food) | Children's airtime | Pre-policy | Post-policy | 0.29 (17) | 0 (0) | NR | NR |  |
| Ofcom 2010 | 7 | HFSS impacts (number of times an HFSS advertisement is seen) in billions (% of all food impacts) |  | Pre-policy | Post-policy | 12.1 (80.7) | 7.7 (58.9) | NR | NR |  |
| Ofcom 2010 | 7 | HFSS impacts (number of times an HFSS advertisement is seen) in billions | Children (4-9y) | Pre-policy | Post-policy | 5.9 | 2.8 | 52% decrease | NR | △ |
| Ofcom 2010 | 7 | HFSS impacts (number of times an HFSS advertisement is seen) in billions | Children (10-15y) | Pre-policy | Post-policy | 6.2 | 4.8 | 22% decrease | NR |  |
| Potvin Kent 2011a | 5 | Number of food and beverage advertisements (% of all advertisements) | Ontario **English** v Quebec **French** | Self-regulation | Government | 117 (23.5) | 119 (26.6) | X2=5.6 | <0.06 | € |
| Potvin Kent 2011a | 5 | Number of food and beverage advertisements (% of all advertisements) | Ontario English v Quebec English | Self-regulation | Government | 117 (23.5) | 151 (26.7) | X2=5.6 | <0.06 |  |
| Potvin Kent 2011a | 5 | Food and beverage advertisements per hour | Ontario English v Quebec French | Self-regulation | Government | 3.9 | 4 | NR | NR |  |
| Potvin Kent 2011a | 5 | Food and beverage advertisements per hour | Ontario English v Quebec English | Self-regulation | Government | 3.9 | 5 | NR | NR |  |
| Potvin Kent 2011b | 5 | Average duration of food and beverage advertisements (SD) |  | Non-signatories | Signatories | 18.6 (9.7) | 21.3 (9.2) | t=3.028 | 0.003 |  |
| Potvin Kent 2011b | 5 | Number of food and beverage advertisements (% of promotions in that arm) |  | Non-signatories | Signatories | 165 (93) | 216 (71) | NR | 0.009 |  |
| Potvin Kent 2011b | 5 | Number of food and beverage advertisements (% of promotions in that arm) | Children's TV | Non-signatories | Signatories | 120 (93) | 181 (69) | NR | 0.001 |  |
| Potvin Kent 2011b | 5 | Number of food and beverage advertisements (% of promotions in that arm) | Generalist TV | Non-signatories | Signatories | 45 (92) | 35 (85) | NR | 0.264 |  |
| Potvin Kent 2011b | 5 | Number of food and beverage promotions classified as less healthy (% of promotions in that arm) | Less healthy foods | Non-signatories | Signatories | 97 (55) | 243 (80) | X2=34.0 | 0.001 | ▼ |
| Potvin Kent 2012 | 5 | Number of food and beverage advertisements | Ontario English v Quebec English | Self-regulation | Government | 137 | 171 | NR | NR |  |
| Potvin Kent 2012 | 5 | Number of food and beverage advertisements | Ontario English v Quebec French | Self-regulation | Government | 137 | 119 | NR | NR |  |
| Potvin Kent 2012 | 5 | Proportion of food and beverage advertisements that were for 'less healthy' items | Ontario English v Quebec English | Self-regulation | Government | 68.3 | 68.9 | X2=22.2 | <0.001 |  |
| Potvin Kent 2012 | 5 | Proportion of food and beverage advertisements that were for 'less healthy' items | Ontario **English** v Quebec **French** | Self-regulation | Government | 68.3 | 60.6 | X2=22.2 | <0.001 | ▲ |
| Potvin Kent 2014b | 6 | Number and proportion (%) of food or beverage advertisements on children's speciality channels |  | Pre-policy | Post-policy | 3591 (80.6) | 2737 (64.3) | 24% decrease | NR |  |
| Potvin Kent 2014b | 6 | Proportion % of food and beverages deemed 'less healthy' | Less healthy foods | Pre-policy | Post-policy | 53.9 | 52.3 | X2=1.41 | 0.235 | € |
| Potvin Kent 2018a | 6 | Number and proportion (%) of food advertisements |  | Non-signatories | Signatories | 18554289 (34.4) | 35449136 (65.6) | NR | NR |  |
| Potvin Kent 2018a | 6 | Proportion of food advertisements for 'ultra processed' items | Less healthy foods | Non-signatories | Signatories | 88.2 | 94.9 | X2=7.4x10^5^, OR 2.5 99% CI 2.5, 2.5 | <0.001 |  |
| Potvin Kent 2018a | 6 | Number and proportion (%) of food advertisements for 'less healthy' items | Less healthy foods | Non-signatories | Signatories | 5720043 (59.0) | 23979000 (78.4) | X2=1.4x10^6^, OR 2.53 99% CI 2.52, 2.53 | <0.001 | ▼ |
| Potvin Kent 2018b | 7 | Number of food and beverage advertisements |  | no uniform nutrition criteria | uniform nutrition criteria | 242 | 334 | 38% increase | NR |  |
| Potvin Kent 2018b | 7 | Number of food and beverage advertisements |  | no uniform nutrition criteria | uniform nutrition criteria | 120 | 187 | 55.8% increase | NR |  |
| Potvin Kent 2018b | 7 | Number of food and beverage advertisements |  | Non-signatories | Signatories | 147 | 187 | NR | NR |  |
| Potvin Kent 2018b | 7 | Number and proportion (%) of food advertisements for 'less healthy' items |  | no uniform nutrition criteria | uniform nutrition criteria | 209 (86.7) | 258 (79.1) | X2=5.48, OR 0.58 | 0.019 |  |
| Potvin Kent 2018b | 7 | Number and proportion (%) of food advertisements for 'less healthy' items |  | no uniform nutrition criteria | uniform nutrition criteria | 112 (93.3) | 146 (78.5) | X2=12.1, OR 0.26 | <0.001 |  |
| Potvin Kent 2018b | 7 | Number and proportion (%) of food advertisements for 'less healthy' items |  | Non-signatories | Signatories (post-implementation of uniform nutrition criteria) | 112 (80.0) | 146 (78.5) | NR | NR | ▽ |
| Potvin Kent 2018b | 7 | Number and proportion (%) of food advertisements for 'less healthy' items |  | no uniform nutrition criteria | uniform nutrition criteria | 191 (81.6) | 146 (78.5) | X2=0.64, OR 0.82 | 0.424 |  |
| Powell 2010 | 7 | Number of food advertisements per day | Children (2-5y) | Pre-policy | Post-policy | 12.1 | 11.5 | NR | NR |  |
| Powell 2010 | 7 | Number of food advertisements per day | Children (6-11y) | Pre-policy | Post-policy | 13.5 | 13.1 | NR | NR |  |
| Powell 2010 | 7 | Mean number of food advertisements per day | Children (2-5y) | Pre-policy | Post-policy | 2.27 | 1.95 | NR | NR |  |
| Powell 2010 | 7 | Mean number of food advertisements per day | Children (6-11y) | Pre-policy | Post-policy | 2.62 | 2.48 | NR | NR |  |
| Powell 2010 | 7 | Mean number of food advertisements per day | Children (2-5y) | Pre-policy | Post-policy | 5.03 | 1.95 | NR | NR |  |
| Powell 2010 | 7 | Mean number of food advertisements per day | Children (6-11y) | Pre-policy | Post-policy | 10.83 | 2.48 | NR | NR | △ |
| Powell 2011 | 7 | Number of food advertisements per day | Children (2-5y) | Pre-policy | Post-policy | 12.1 | 10.9 | NR | NR |  |
| Powell 2011 | 7 | Number of food advertisements per day | Children (6-11y) | Pre-policy | Post-policy | 13.5 | 12.7 | NR | NR |  |
| Powell 2011 | 7 | Mean number of food advertisements per day | Children (2-5y) | Pre-policy | Post-policy | 8.5 | 6.7 | NR | NR |  |
| Powell 2011 | 7 | Mean number of food advertisements per day | Children (6-11y) | Pre-policy | Post-policy | 9.4 | 7.9 | NR | NR |  |
| Powell 2011 | 7 | Proportion of food ads (%) for unhealthy foods | Children (2-5y) | Pre-policy | Post-policy | 89.7 | 88.2 | NR | NR |  |
| Powell 2011 | 7 | Proportion of food ads (%) for unhealthy foods | Children (6-11y) | Pre-policy | Post-policy | 89.3 | 88.2 | NR | NR |  |
| Powell 2013 | 7 | Total food advertisements seen per year | Children (2-5y) | Non-signatories | Signatories | 1541 | 2442 | NR | NR |  |
| Powell 2013 | 7 | Total food advertisements seen per year | Children (6-11y) | Non-signatories | Signatories | 1755 | 2887 | NR | NR |  |
| Powell 2013 | 7 | Total food advertisements seen per year during children's programming (% of all programming) | Children (2-5y) | Non-signatories | Signatories | 448 (29.1) | 1389 (56.9) | NR | NR |  |
| Powell 2013 | 7 | Total food advertisements seen per year during children's programming (% of all programming) | Children (6-11y) | Non-signatories | Signatories | 426 (24.3) | 1588 (55.0) | NR | NR |  |
| Powell 2013 | 7 | Proportion of food or beverage product ads that are 'high in nutrients to limit' | Children (2-5y) | Non-signatories | Signatories | 71.4 | 87.4 | NR | NR |  |
| Powell 2013 | 7 | Proportion of food or beverage product ads that are 'high in nutrients to limit' | Children (6-11y) | Non-signatories | Signatories | 72.7 | 87.2 | NR | NR |  |
| Powell 2013 | 7 | Proportion of food or beverage product ads that are 'high in nutrients to limit' during children's programming | Children (2-5y) | Non-signatories | Signatories | 80.5 | 97.8 | NR | NR |  |
| Powell 2013 | 7 | Proportion of food or beverage product ads that are 'high in nutrients to limit' during children's programming | Children (6-11y) | Non-signatories | Signatories | 89.9 | 98.1 | NR | NR | ▽ |
| Theodore 2017 | 6 | Number and proportion (% of all food ads recorded) of food or beverage advertisements to children |  | Non-signatories | Signatories | 298 (13.9) | 1850 (86.1) | NR | NR |  |
| Theodore 2017 | 6 | Number and proportion (% of food ads within arm) of ultraprocessed foods advertised to children |  | Non-signatories | Signatories | 297 (99.7) | 1715 (92.7) | NR | NR | ▽ |
| Vergeer 2019 | 5 | Proportion (%) of companies with child-directed marketing on their website |  | Non-signatories | Signatories | 14 | 56 | X2=7.30 | 0.03 |  |
| Vergeer 2019 | 5 | Proportion (%) of products marketed to children on company websites that exceeded 15% DV |  | Non-signatories | Signatories | 20.6 | 79.4 | NR | NR | ▽ |
| Warren 2007 | 5 | Frequency of food advertisements |  | Pre-policy | Post-policy | 1880 | 2898 | NR | NR |  |
| Warren 2007 | 5 | Frequency of food advertisements | Child targeted | Pre-policy | Post-policy | 781 | 1556 | NR | NR | ▽ |
| Warren 2007 | 5 | Frequency of food advertisements | In child-rated shows | Pre-policy | Post-policy | 522 | 1086 | NR | NR |  |
| Whalen 2017 | 6 | Proportion (%) of food advertisements |  | Partial implementation | Full implementation – | 12.8 | 11.9 | NR | NR |  |
| Whalen 2017 | 6 | Proportion (%) of food advertisements | Dedicated children's channels | Partial implementation - | Full implementation | 8.2 | 3.9 | NR | NR |  |
| Whalen 2017 | 6 | Proportion (%) of food advertisements for non-core items |  | Partial implementation - weaker | Full implementation | 56.0 | 53.8 | NR | NR |  |
| Whalen 2017 | 6 | Proportion (%) of food advertisements for non-core items | Dedicated children's channels | Partial implementation - weaker | Full implementation | 59.8 | 51.2 | NR | NR | △ |
| Whalen 2017 | 6 | Proportion (%) of food advertisements | Peak children's viewing times | Partial implementation - weaker | Full implementation | 15.3 | 17 | NR | NR |  |

**Table S2: Power data**

| **Study name** | **NOS** | **Measure of effect** | **Details of subgroup (if relevant)** | **Name of arm 1** | **Name of arm 2** | **Value arm 1** | **Value arm 2** | **Effect size** | **p value** | **Effect direction** |
| --- | --- | --- | --- | --- | --- | --- | --- | --- | --- | --- |
| Brindal 2011 | 5 | Number (%) of non-core food ads using promotional character |  | Non-signatories | Signatories | 0 (0) | 6 (2.9) | NR | NR | ▽ |
| Brindal 2011 | 5 | Number (%) of non-core food ads using promotional character |  | Non-signatories | Signatories | 0 (0) | 0 (0) | NR | NR |  |
| Brindal 2011 | 5 | Number (%) of non-core food ads using promotional character |  | Non-signatories | Signatories | 37 (72.5) | 3 (1.5) | NR | NR |  |
| Brindal 2011 | 5 | Number (%) of non-core food ads using promotional character |  | Non-signatories | Signatories | 0 (0) | 12 (57.1) | NR | NR |  |
| Brindal 2011 | 5 | Number (%) of non-core food ads using premiums |  | Non-signatories | Signatories | 0 (0) | 2(1) | NR | NR |  |
| Brindal 2011 | 5 | Number (%) of non-core food ads using premiums |  | Non-signatories | Signatories | 0 (0) | 11 (6) | NR | NR |  |
| Brindal 2011 | 5 | Number (%) of non-core food ads using premiums |  | Non-signatories | Signatories | 0 (0) | 0 (0) | NR | NR |  |
| Brindal 2011 | 5 | Number (%) of non-core food ads using premiums |  | Non-signatories | Signatories | 0 (0) | 0 (0) | NR | NR |  |
| Effertz 2012 | 5 | Propensity for non-core food advertisements (vs. all other ads) to contain a promotional character | Non-core food advertising using promotional characters | Pre-policy | Post-policy | NR | NR | OR = 4.188 | <0.001 | ▼ |
| Effertz 2012 | 5 | Propensity for non-core food advertisements (vs. all other ads) to contain a premium | Non-core food advertising using premiums | Pre-policy | Post-policy | NR | NR | OR = 0.223 | <0.001 |  |
| Galloway 2014 | 5 | Number (proportion, %) of food products |  | Non-signatories | Signatories | 66 (21.5) | 241 (78.5) | NR | NR |  |
| Galloway 2014 | 5 | Overall nutritional quality comparison (proportion of whoa products) |  | Non-signatories | Signatories | 35 (53) | 194 (80.5) | X2=21.5133 | <0.0001 | ▼ |
| Harris 2015 | 7 | % of unique candy advertisements featuring any child-targeted technique | NA | Non-participating brands | Participating companies, non-approved brands | 59 | 77 | NR | <0.05 |  |
| Harris 2015 | 7 | Number (%) of candy advertisements viewed featuring child-targeted techniques | Children (2-11y) | Non-participating brands | Participating companies, non-approved brands | 107 (22) | 287 (59) | NR | NR | ▽ |
| Harris 2015 | 7 | Number (%) candy advertisements viewed featuring child-targeted techniques | Adolescents (12-17y) | Non-participating brands | Participating companies, non-approved brands | 67 (10) | 543 (77) | NR | NR |  |
| King 2011 | 6 | Number (%) of non-core food advertisements using persuasive techniques | Non-core food advertising | Non-signatories | Signatories | 69 (38) | 112 (62) | NR | NR | ▽ |
| Kunkel 2015 | 6 | Food advertisements featuring a licensed character (as a % of all food ads) |  | Pre-policy | Post-policy | 13.0 | 11.6 | Z=-0.63 | 0.53 |  |
| Kunkel 2015 | 6 | Unhealthy (whoa) food advertisements featuring a licensed character as a proportion (%) of all food advertisements | Whoa food advertising | Pre-policy | Post-policy | 91.4 | 61.0 | Z=-4.05 | <0.001 | ▲ |
| Mediano 2019 | 6 | Proportion of "high in" cereals using at least one child-directed marketing strategy |  | Pre-policy | Post-policy | 43.18 | 15.12 | NR | <0.05 | ▲ |
| Mediano 2019 | 6 | Likelihood of breakfast cereal packages featuring child-directed marketing strategies |  | Non-high in | High-in | 8.33 | 43.18 | OR 8.36 (95%CI 2.44, 28.63) | <0.01 |  |
| Mediano 2019 | 6 | Likelihood of breakfast cereal packages featuring child-directed marketing strategies |  | Non-high in | High-in | 30 | 15.12 | OR 0.416 (95%CI 0.19, 0.93) | <0.05 |  |
| Morton 2005 | 5 | Number and proportion (%) of food advertisements that featured premium offers |  | Self-regulation | Government | 76 (17) | 35 (36) | X2=17.9 | <0.0001 | ▼ |
| Morton 2005 | 5 | Rate of food advertisements that featured premium offers (ads/hour) |  | Self-regulation | Government | 1.5 | 2.8 | NR | NR |  |
| Neyens 2017 | 5 | Presence of brand benefit claims (dichotomised yes/no; Mann-Whitney mean ranks) |  | Non-signatories | Signatories | 28.26 | 17.6 | U=144, z=-2.41, r=0.34 | 0.016 |  |
| Neyens 2017 | 5 | Presence of spokescharacters (dichotomised yes/no; Mann-Whitney mean ranks) |  | Non-signatories | Signatories | 21.94 | 31.93 | U=359, z=2.39, r=0.34 | 0.017 | ▼ |
| Ofcom 2008 | 7 | Number of spots featuring licensed characters (% of all food and drink advertisements) |  | Pre-policy | Post-policy | 110000 (4.1) | 48000 (1.1) | 56% decrease overall, 69% fall in child impacts | NR | △ |
| Ofcom 2008 | 7 | Number of spots featuring celebrities (% of all food and drink advertisements) |  | Pre-policy | Post-policy | 228000 (7.8) | 476000 (11.5) | 109% increase overall, 22% increase in child impacts | NR |  |
| Ofcom 2008 | 7 | Number of spots featuring promotions (% of all food and drink advertisements) |  | Pre-policy | Post-policy | 206000 (7.6) | 368000 (8.8) | 79% increase overall, 36% fall in child impacts | NR |  |
| Ofcom 2010 | 7 | Child impacts (billions) for food and drink ads using a licensed character |  | Pre-policy | Post-policy | 0.39 | 0.06 | 84% decrease | NR | △ |
| Ofcom 2010 | 7 | Child impacts (billions) for food and drink ads using a celebrity |  | Pre-policy | Post-policy | 0.2 | 0.4 | 143% increase | NR |  |
| Ofcom 2010 | 7 | Child impacts (billions) for food and drink ads using promotions |  | Pre-policy | Post-policy | 0.5 | 0.3 | 41% decrease | NR |  |
| Potvin Kent 2011a | 5 | Number of food ads (% of all food ads) using persuasive appeal of fun | Ontario English v Quebec French | Self-regulation | Government | 44 (37.6) | 18 (15.1) | X2=22.9 | <0.001 |  |
| Potvin Kent 2011a | 5 | Number of food ads (% of all food ads) using persuasive appeal of fun | Ontario English v Quebec English | Self-regulation | Government | 44 (37.6) | 62 (41.1) | X2=22.9 | <0.001 |  |
| Potvin Kent 2011a | 5 | Number of food ads (% of all food ads) featuring media character or celebrity | Ontario English v Quebec French | Self-regulation | Government | 28 (23.9) | 14 (11.7) | X2=6.3 | <0.04 | ▲ |
| Potvin Kent 2011a | 5 | Number of food ads (% of all food ads) featuring media character or celebrity | Ontario English v Quebec English | Self-regulation | Government | 28 (23.9) | 32 (21.2) | X2=6.3 | <0.04 |  |
| Potvin Kent 2011b | 5 | Number of promotions featuring media characters (% of promotions in that arm) |  | Non-signatories | Signatories | 27 (15) | 91 (30) | NR | <0.001 |  |
| Potvin Kent 2011b | 5 | Number of promotions featuring media characters (% of of promotions in that arm) | Children's TV | Non-signatories | Signatories | 20 (16) | 78 (30) | NR | 0.002 |  |
| Potvin Kent 2011b | 5 | Number of promotions featuring media characters (% of promotions in that arm) | Generalist TV | Non-signatories | Signatories | 7 (14) | 13 (32) | NR | NR |  |
| Potvin Kent 2011b | 5 | Number of promotions featuring media characters (% of promotions in that arm) that were for less healthy foods | Less healthy foods | Non-signatories | Signatories | 13 (48) | 82 (90) | X2=23.4 | <0.001 |  |
| Potvin Kent 2011b | 5 | Number of promotions featuring media characters (% of promotions in that arm) that were for less healthy foods | Less healthy foods, Children's TV | Non-signatories | Signatories | 9 (45) | 72 (92) | X2=24.8 | <0.001 | ▼ |
| Potvin Kent 2013 | 5 | Number (%) of websites with spokescharacters |  | Non-signatories | Signatories | 6 (46.2) | 8 (57.1) | X2=0.326 | 0.568 | € |
| Potvin Kent 2013 | 5 | Number (%) of websites with child-directed content |  | Self-regulation | Government | 27 (35) | 22 (31) | X2=0.218 | <0.640 |  |
| Potvin Kent 2013 | 5 | Number (%) of websites with child-directed content |  | Non-signatories | Signatories | 13 (34) | 14 (38) | X2=0.24 | 0.877 |  |
| Potvin Kent 2014b | 6 | Number (%) of less healthy food and beverage ads using licensed characters |  | Pre-policy | Post-policy | 64 (23.0) | 214 (15.0) | 234% increase | NR | ▽ |
| Vaala 2020 | 5 | Number of different features, mean (SD) |  | Non-signatories | Signatories | 0.4 (0.91) | 2.8 (1.55) | NR | <0.05 |  |
| Vaala 2020 | 5 | Number of different features, mean (SD) | Cereals with high sugar content | Non-signatories | Signatories | 1.4 (1.33) | 3.6 (1.21) | NR | NR | ▽ |
| Vergeer 2019 | 5 | Proportion (%) of companies with child-directed marketing featuring marketing techniques on the website |  | Non-signatories | Signatories | 6.7 | 93.3 | NR | NR | ▽ |
| Warren 2007 | 5 | Proportion of food advertisements featuring the persuasive appeal of taste |  | Pre-policy | Post-policy | 32.9 | 34.5 | NR | NR |  |
| Warren 2007+MB51:P51 | 5 | Proportion of food advertisements featuring the persuasive appeal of taste | Child targeted | Pre-policy | Post-policy | 26.4 | 31.3 | NR | NR |  |
| Warren 2007 | 5 | Proportion of food advertisements featuring the persuasive appeal of taste | In child-rated shows | Pre-policy | Post-policy | 31.8 | 31.2 | NR | NR |  |
| Warren 2007 | 5 | Proportion of food advertisements featuring the production technique of animation |  | Pre-policy | Post-policy | 19.5 | 34.1 | X2=14.01, V=0.05 | <0.001 |  |
| Warren 2007 | 5 | Proportion of food advertisements featuring the production technique of animation | Child targeted | Pre-policy | Post-policy | 25.9 | 35.7 | 10% increase; X2=23.12, V=0.10 | <0.001 | ▼ |
| Warren 2007 | 5 | Proportion of food advertisements featuring the production technique of animation | In child-rated shows | Pre-policy | Post-policy | 40.4 | 46.4 | 6% increase; X2=5.12, V=0.06 | <0.05 |  |

**Table S3: Purchasing data**

| **Study name** | **NOS** | **Measure of effect** | **Details of subgroup (if relevant)** | **Name of arm 1** | **Name of arm 2** | **Value arm 1** | **Value arm 2** | **Effect size** | **p value** | **Effect direction** |
| --- | --- | --- | --- | --- | --- | --- | --- | --- | --- | --- |
| Dhar 2011 | 8 | Difference in fast food purchase incidence per week between FP and AP households in Ontario and Quebec | NA | Self-regulation | Government | -0.032 | -0.134 | -0.102 | <0.05 | ▲ |
| Dhar 2011 | 8 | Difference in expenditure on fast food between FP and AP households in Ontario and Quebec | NA | Self-regulation | Government | -0.11 | -1.31 | -1.2 | NS |  |
| Huang 2013 | 8 | Households with children bubble gum purchase change, relative purchase frequency (SD) | Children <12y | Pre-policy | Post-policy | NR | NR | Decrease 2.486 (0.684) | <0.01 | ▲ |
| Silva 2015 | 8 | Total household expenditure (per capita, per quarter) on HFSS food |  | Pre-policy | Post-policy (self) |  | -0.03 | £8.7 decrease | <0.01 |  |
| Silva 2015 | 8 | Total household expenditure (per capita, per quarter) on HFSS food |  | Pre-policy | Post-policy (Co) |  | -0.02 | £6.2 decrease | <0.01 | ▲ |
| Silva 2015 | 8 | Total household expenditure (per capita, per quarter) on HFSS drinks |  | Pre-policy | Post-policy (self) |  | -0.01 | £2.9 decrease | <0.01 |  |
| Silva 2015 | 8 | Total household expenditure (per capita, per quarter) on HFSS drinks |  | Pre-policy | Post-policy (Co) |  | -0.01 | £2.7 decrease | <0.01 |  |
| Otten 2014 | 5 | Purchase of children's meals, n (%) |  | Pre-policy | Post-policy | 88 (35.8) | 123 (47.3) | NR | NR | ▼ |
| Otten 2014 | 5 | Frequency of eating at fast food restaurant per month, mean n (SD) |  | Pre-policy | Post-policy | 4.5 (4.8) | 4.8 (5.2) | NR | NR |  |
| Otten 2014 | 5 | Mean calories per order |  | Pre-policy | Post-policy | 662 (351) | 654 (318) | NR | 0.02 |  |
| Otten 2014 | 5 | Mean calories per order |  | Pre-policy | Post-policy | 533 (170) | 530 (188) | NR | 0.82 |  |
| Lwin 2020 | 5 | Amount of unhealthy food in the household pantry, mean (SD) |  | Pre-policy | Post-policy | 721.65 (806.94) | 526.16 (736.11) | t(209)=3.43 | 0.001 | ▲ |

**Table S4: Dietary intake data**

| **Study name** | **NOS** | **Measure of effect** | **Details of subgroup (if relevant)** | **Name of arm 1** | **Name of arm 2** | **Value arm 1** | **Value arm 2** | **Effect size** | **p value** | **Effect direction** |
| --- | --- | --- | --- | --- | --- | --- | --- | --- | --- | --- |
| Lwin 2020 | 5 | Mean unhealthy food consumption score |  | Pre-policy | Post-policy | 1.97 (.72) | 1.91 (.69) | NR | 0.03 | ▲ |

**Table S5: Product change data**

| **Study name** | **NOS** | **Measure of effect** | **Details of subgroup (if relevant)** | **Name of arm 1** | **Name of arm 2** | **Value arm 1** | **Value arm 2** | **Effect size** | **p value** | **Effect direction** |
| --- | --- | --- | --- | --- | --- | --- | --- | --- | --- | --- |
| Clark 2007 | 7 | Average price of cereal per 100g | Children's brands | No policy | Policy | $0.87 | $0.89 | 0.025% | 0.08 | € |
| Vaala 2020 | 5 | Sugar per ounce in g, mean (SD) |  | Non-signatories | Signatories | 6.6 (3.44) | 8.7 (2.22) | NR | <0.05 | ▼ |

**Table S6: Unintended consequences data**

| **Study name** | **NOS** | **Measure of effect** | **Details of subgroup (if relevant)** | **Name of arm 1** | **Name of arm 2** | **Value arm 1** | **Value arm 2** | **Effect size** | **p value** | **Effect direction** |
| --- | --- | --- | --- | --- | --- | --- | --- | --- | --- | --- |
| Kim 2013 | 8 | Total advertising budget for EDNP foods in $ | EDNP food advertising | Pre-policy | Post-policy | 420,000 | 2000 | NR | NR | △ |
| Ofcom 2008 | 7 | % change in net food and drink advertising revenue | Main commercial channels | Pre-policy | Post-policy | NR | NR | -6 | NR |  |
| Ofcom 2008 | 7 | % change in net food and drink advertising revenue | Children's channels | Pre-policy | Post-policy | NR | NR | -26 | NR | △ |
| Ofcom 2008 | 7 | % change in net advertising revenue | Main commercial channels | Pre-policy | Post-policy | NR | NR | -11 | NR |  |
| Ofcom 2008 | 7 | % change in net advertising revenue | Children's channels | Pre-policy | Post-policy | NR | NR | 35 | NR |  |
| Silva 2015 | 8 | Regulation coefficients of the upper limit of regulation impact |  | Pre-policy | Post-policy (self) |  | -4.23 | NR | NR |  |
| Silva 2015 | 8 | Regulation coefficients of the upper limit of regulation impact |  | Pre-policy | Post-policy (Co) |  | -15.2 | £15.2 million decrease in TV HFSS (19.4%) | <0.01 | ▲ |

**Appendix C: Policy information**

**Table S7. Key characteristics of policies evaluated by included studies**

**Key:**

NR – not reported

Mandatory – Government-enforced policy

Vol – voluntary measure

| Policy Name | Jurisdiction (date implemented) | Policy type | Definition of child  in policy | TARGETED PRODUCTS | | EXPOSURE | | POWER |
| --- | --- | --- | --- | --- | --- | --- | --- | --- |
|  |  |  |  | **Target foods and beverages** | **Criteria/model used to define** | **Restricted communications, channels, and settings** | **How are child-directed communications, media, ads, settings defined?** | **How are marketing techniques defined and restricted?** |
| Australian Children's Television Standards (CTS) | Australia  (1984) | Mandatory | 6-13 years | All foods and drinks | NR | TV | Placement: Programs and advertisements  shown during designated children’s ‘C’ programs (those  specifically produced for children six to 13 years of age) | Regulates (not prohibits) use of promotions, popular characters and premium offers promoted to children in advertisements for food: ‘If a premium is offered, any reference  to the premium must be incidental to the main product or  service advertised.’ Premiums are defined as anything offered with or without additional cost that is intended to induce the purchase of an advertised product or service |
| Australian Food and Grocery Council’s (AFGC) Responsible Marketing to Children Initiative (RCMI) | Australia  (Jan, 2009) | Vol | <12 years | Those not representing healthier choices as per established scientific or Australian Government standards | Company-specific nutrition standards | TV, radio, print, cinema, third party internet sites | Audience: media “…where the audience is predominantly children and/or the media or communication activities are directed primarily to children” | Popular personalities and licensed characters (defined as a character from C (children’s) or P (preschool children’s) program, other program or movie and all non-copyright cartoons) may only be used to promote healthier dietary choices, premium offers (may only be used if premium is incidental to food product advertised) |
| Australian Food and Grocery Council’s (AFGC) Australian Quick Service Restaurant Industry Initiative (QSRI) | Australia  (Aug, 2009) | Vol | <14 years | Those not representing healthier choices as per established scientific or Australian Government standards | Defined set of nutrition criteria for assessing children's meals | TV, radio, newspaper, magazines, outdoor billboards and posters, emails, interactive games, cinema and internet sites | Medium that is directed primarily to Children (in relation to television this includes all C and P rated programs and other rated programs that are directed primarily to Children through their themes, visuals, and language); and/or where children represent 35 per cent or more of the audience of the Medium | NR |
| Canadian Children's Food and Beverage Advertising (CAI) Initiative (CAI) | Canada (introduced 2007, fully implemented by 2008) | Vol | <12 years | Non "healthier dietary choices" | Uniform nutrition criteria: company-specific nutrition standards | TV, radio, print, internet | Audience and placement: Company-owned websites/micro-sites primarily directed to children <12 years; video/computer games rated 'Early Childhood (EC)'; DVDs of movies rated 'G' whose primary content is primarily directed to children <12 years, and other DVDs whose content primarily directed to children <12 years; mobile media (phones, tablets, personal digital devices) where advertising on those media is primarily directed to children <12 years | Licensed characters, celebrities, movie tie-ins, use of products in interactive games, product placement. |
| Children’s Food and Beverage Advertising Initiative (CFBAI) | US  (introduced 2007, fully implemented by 2009) | Vol | <12 years | Non "better for you" products | Uniform nutrition criteria | TV, radio, print, internet/digital media | Audience and setting: children make up 35% minimum of the audience. Elementary schools, entire school, facilities, grounds and covers the entire school day. | Influencer communications, product placements, licensed characters, celebrities, movie tie-ins and word of mouth.  Word of mouth advertising refers to ‘advertising primarily directed to children under age 12 where a participant provides incentives, (financial or otherwise), product samples or other support to individuals or groups who are not employees to encourage such individuals or groups to discuss the company’s branded foods or beverages.’ |
| Chile Food Labelling and Advertising Regulation ("Super 8 Law") | Chile  (2016, updated 2018) | Mandatory | <14 years | "High-in" products | Uniform nutrition criteria: thresholds set by the Chilean Ministry of Health | TV, websites, schools, packaging | Time, placement, audience, and setting: all TV broadcast from 6:00 to 22:00. Outside of these hours, TV broadcast on devoted children’s channels; during programs targeting children; or when child audience is > 20% (except during sports, cultural, artistic, or charity events, if certain criteria are met). Also included are websites targeting children or those with child audience of > 20%; preschools primary and secondary schools | Prohibits, in any marketing for regulated products, use of the following: celebrities, characters, cartoons (including brand equity); toys; stickers; animations; children’s music; people/animals that capture children’s interest; fantastic statements about product or its effects; situations representing children’s daily life; children’s expressions or language; interactive contests, games, or applications; or ‘hooks’ unrelated to the product itself |
| EU Pledge | European Union (EU; introduced 2007, uniform nutrition criteria adopted 2014) | Vol | <12 years | Those primarily directed to children under 12 that are not meeting specific nutrition criteria | Company-specific nutrition standards | TV, radio, cinema, print, outdoor marketing, internet, mobile apps, social networking websites, influencer marketing, interactive games, schools | Audience, placement and setting: no advertising to media audiences with >35% of children <12 years (from 2012, previously 50%), except for products which meet nutrition criteria (company-specific). No communication related to products in primary schools, except where specifically requested, or agreed with, the school administration for educational purposes. Since 2012, internet advertising has been extended to include company-owned websites also, in addition to third-party advertising. | Prohibits advertising of products that do not meet common nutrition criteria to under 12 year olds, or any products at all to under 12 year olds |
| European and Spanish Public Health laws | Spain  (Jul, 2011) | Mandatory | <15 years | NR | NA | TV and other ‘food publicity’ | NR | NA |
| Mexican Self-regulation | Mexico  (2009) | Vol | <12 years | No specific targets but to be permitted advertisements must “promote healthy lifestyle habits, based on a proper diet and active lifestyle” | NR | TV, radio | Audience, placement and setting: applies to schedules and  programs predominantly aimed at children (without  further speciﬁcation).  Defined as child-directed if the product or packaging and/or the advertisement (through themes  related to fantasy, mystery, or adventure, or use of colourful characters and gifts) aims to appeal to children, and/or when an advertisement is broadcast on children’s programming or when a child audience reaches a pre-established minimum level. | Does not prohibit the use of persuasive techniques |
| Quebec Consumer Protection Act | Quebec, Canada (1980) | Mandatory | <13 years | Those designed for or of primary appeal to children | Any product consumed primarily by children | All commercial advertising directed at children | Audience: child-directed when children make up >15% of audience | Use of characters or themes designed to elicit the interest of children |
| San Francisco Healthy Food Incentives Ordinance | San Francisco, US  (Dec, 2011) | Mandatory | NR | Those not meeting nutrition criteria | Uniform nutrition criteria | Fast-food restaurants in San Francisco | Settings: applies to all fast-food restaurants | Free toys or incentives (games, trading cards, or other consumer products) |
| Singapore Code of Advertising Practice (SCAP) | Singapore  (Jan, 2015) | Vol | <12 years | Those not meeting nutrition criteria | Uniform nutrition criteria: determined by the Health Promotion Board | All media | NR | Diet and lifestyle messaging: Should not encourage unhealthy or excessive eating or undermine role of caregivers as guide for children's dietary choices |
| Special Act on Safety Management of Children's Dietary Life | Republic of Korea (Sept, 2010) | Mandatory | 4-18 years | Food products favored by children as snack or meal substitutes that do not satisfy the nutrition criteria | Uniform nutrition criteria: determined by Korean Food & Drug Administration | TV | NR | NR |
| UK content and scheduling (Ofcom) restrictions | UK  (Apr 2007 - Jan 2009) | Mandatory | <16 years | Those high in fats, sugar or salt (HFSS) | UK Food Standard's Agency Nutrient Profiling Model | TV | Placement and audience: broadcasting during children’s programs or when proportion of viewers aged 4–15 years is 20%  higher than in the general  population | Promotional offers, nutritional and health claims, licensed characters, celebrities. Techniques regulated by UK Code of Broadcast Advertising (BCAP) code; calculated to appeal to children aged 4-16 |

**Appendix D: Subgroup analyses for comparison 1**

***Subgroup analyses for exposure:***

- ***The age range of children sought for protection***

Twenty-five studies^1-25^ evaluated policies to restrict food marketing (vs. no policy) in which the definition of the age of a child in the policy was 12 years or under (and therefore the policy aimed to restrict food marketing to this age group), and 8 studies^26-33^ evaluated policies or measures (vs. no policy or measure) in which the definition of the age of a child included children over 12 years (see Table S7 for specific definitions of age of children for each policy).

**Summary box. Comparison 1: Exposure results - subgroup analyses by age of child in policy**

For studies of measures where children sought for protection by the policy were **12 years or under**, two studies clearly favored the intervention^9,14^ (10% (95% CI 1.8% to 33.1%), p<.001).

Five studies potentially favored the intervention^3,5,10,21,22^ (21.7% (95% CI 8.3% to 42.2%), p=.012).

Seven studies clearly or potentially favored the intervention (28% (95% CI 12.9% to 50.0%), p=.046).

Seven of 25 studies were judged to be high quality and, of these, one clearly favored the intervention^9^ and two potentially favored the intervention^21,22^.

For studies of policies/measures in which children sought for protection by the policy included children **over 12 years**, one study clearly favored the intervention^28^ (33.3% (95% CI 1.7% to 87.5%), p=1.000).

Five studies potentially favored the intervention^29-33^ (71.4% (95% CI 30.3% to 94.9%), p=.449).

Six studies clearly or potentially favored the intervention (75% (95% CI 35.6% to 95.5%), p=.289).

Four of 25 studies were judged to be high quality, and, of these, no studies clearly favored the intervention, and three studies potentially favored the intervention^30,32,33^.

Some studies reported relevant exposure effect measures for two age groups, however only one effect measure could be included in the above evidence synthesis^3,5-7,20-22,28,32,33^. Importantly, however, where both age groups were captured by the definition of children in the policy/measure being evaluated, results were consistent across the two effect measures. For example, Dillman Carpentier (2020)^28^ reported desirable effects of the policy (child defined as <14 years) on exposure in both preschoolers (mean age 4.8 years) and adolescents (mean age 13.6 years), Ofcom (2008)^32^ and Ofcom (2010)^33^ reported potentially desirable effects of the policy (child defined as 4-15 years) on exposure in both 4-9 and 10-15 year old children, and Powell (2010)^22^ and Powell (2011)^21^ reported potentially desirable effects of the policy (child defined as <12 years) on exposure in both 2-5 and 6-11 year old children. Notably, the two effect measures in Powell (2013)^20^ were also consistent but in the opposite direction (potentially undesirable effects on exposure for 2-5 and 6-11 year old children). This may reflect differences in study design as Powell (2013)^20^ was a cross-sectional survey comparing signatories and non-signatories to a voluntary measure (CFBAI) whereas the earlier studies used repeated cross-sectional (pre-post implementation) designs^21,22^. Harris (2018)^7^ also reported potentially undesirable effects of the policy (child defined as < 12 years) on both 2-5 and 6-11 year old children.

Notably, three studies reported effects of the policy on children both within and outside the policy definition of a child^3,5,6^. Harris (2017)^6^ reported potentially undesirable effects of the policy (child defined as <12 years) on exposure in both 2-6 years old children (age captured by the policy) and young teenagers (12-14 years; not captured by the policy), whereas Frazier (2018)^5^ reported potentially desirable effects of the policy (child defined as <12 years) on exposure in both 2-11 year old (age captured by the policy) and 12-17 year old children (not captured by the policy). Conversely, Dembek (2012)^3^ reported potentially desirable effects of the policy (child defined as <12 years) on exposure in 2-11 year old children but concurrent potentially undesirable effects on exposure in 12-17 year old children (age not captured by the policy).

- ***Marketing medium***

As reflects the advertising medium most commonly restricted by the policies evaluated in the included studies, the majority of studies in this comparison (n=29) evaluated the effect of any food marketing policies on exposure to TV food advertising^1-14,16,17,19-23,25-30,32,33^. Three studies^15,18,24^ considered effects of voluntary measures on digital food marketing exposure (all websites) and one study^31^ considered effects of a mandatory policy on food marketing exposure via product packaging.

**Summary box. Comparison 1: Exposure results - subgroup analyses by marketing medium**

For exposure to **TV advertising**, three studies clearly favored the intervention^9,14,28^ (15.0% (95% CI 4.0% to 38.8%), p=.003).

Nine studies potentially favored the intervention^3,5,10,21,22,29,30,32,33^ (34.6% (95% CI 17.9% to 55.6%), p=.168).

Twelve studies clearly or potentially favored the intervention (41.4% (95% CI 24.1% to 60.9%), p=.457).

Eleven of 29 studies were judged to be high quality and, of these, one clearly favored the intervention^9^ and five potentially favored the intervention^21,22,30,32,33^.

For exposure to **digital marketing**, no study (of three) clearly favored or potentially favored the intervention, all were moderate quality.

For exposure to **product packaging**, the only study identified was moderate quality and potentially favored the intervention^31^.

- ***The approach to classifying foods to which the restrictions apply***

Three studies^26,32,33^ evaluated the UK mandatory policy (vs. no policy) that uses a nutrient profile model to categorize foods as HFSS (not permitted to be marketed to children) or non-HFSS (permitted to be marketed to children); 14 studies evaluated voluntary measures that used company specific nutrition thresholds^1,2,4,9-13,16,17,20-22,25^, and 14 studies evaluated policies that used uniform category-specific nutrition criteria^3,5-8,14,15,18,19,24,28-31^. A further 2 studies did not report how foods were classified by the policy^23,27^.

**Summary box. Comparison 1: Exposure results - subgroup analyses by approach used to classify foods to which the restrictions apply**

For studies of policies using a **nutrient profile model**, no studies clearly favored the intervention.

Two studies potentially favored the intervention^32,33^ (66.6% (95% CI 12.5% to 98.2%), p=1.00). All three studies were judged to be high quality.

For studies of policies using **company specific nutritional criteria**, one study clearly favored the intervention^9^ (9.1% (95% CI 0.4% to 42.9%), p=.015).

Three studies potentially favored the intervention^10,21,22^ (23.1% (95% CI 6.2% to 54.0%), p=.096).

Four studies clearly or potentially favored the intervention (28.6% (95% CI 9.6% to 58.0%), p=.181).

Four of 14 studies were judged to be high quality and, of these, one clearly favored the intervention^9^ and two potentially favored the intervention^21,22^.

For studies of policies using **uniform** **category-specific nutritional criteria**, two studies clearly favored the intervention^14,28^ (22.2% (95% CI 3.9% to 59.8%), p=.182).

Five studies potentially favored the intervention^3,5,29-31^ (41.7% (95% CI 16.5% to 71.4%), p=.773).

Seven studies clearly or potentially favored the intervention (50.0% (95% CI 26.8% to 73.2%), p=1.00).

Four of 14 studies were judged to be high quality and, of these, none clearly favored the intervention and one potentially favored the intervention^30^.

***Subgroup analyses for power:***

- ***The age range of children sought for protection***

Twelve studies^2,4,8,11,12,15-17,24,25,34,35^ evaluated effects of any food marketing policy vs no policy in which the definition of the age of a child in the policy was 12 years or under (and therefore the policy aimed to restrict food marketing to this age group), and 4 studies^31-33,36^ evaluated policies in which the definition of the age of a child included children over 12 years (see Table S7 for specific definitions of age of children for each policy).

**Summary box. Comparison 1: Power results - subgroup analyses by age of child in policy**

For studies of policies where children sought for protection by the policy were **12 years or under**, one study clearly favored the intervention^12^ (8.3% (95% CI 0.4% to 40.2%), p=.009).

No studies potentially favored the intervention.

One study clearly or potentially favored the intervention (8.3% (95% CI 0.4% to 40.2%), p=.009).

One of 12 studies was judged to be high quality, it did not clearly favor or potentially favor the intervention.

For studies of policies in which children sought for protection by the policy included those **over 12 years**, one study clearly favored the intervention^31^ (50.0% (95% CI 9.0% to 90.5%), p=1.00).

Two studies potentially favored the intervention^32,33^ (66.7% (95% CI 12.5% to 98.2%), p=1.00).

Three studies clearly or potentially favored the intervention (75.0% (95% CI 21.9% to 98.6%), p=.617).

Two studies were judged to be high quality and both studies potentially favored the intervention^32,33^.

- ***Marketing medium***

Ten studies evaluated the effects of any food marketing policy on the power of TV advertising^2,4,8,11,12,16,17,25,32,33^, three studies^15,24,36^ considered effects of voluntary measures on digital food marketing power (all websites) and three studies^31,34,35^ considered effects of food marketing policies or measures on marketing power via product packaging.

**Summary box. Comparison 1: Power results - subgroup analyses by marketing medium**

For power of **TV advertising**, one study clearly favored the intervention^12^ (12.5% (95% CI 0.6% to 53.3%), p=.077).

Two studies potentially favored the intervention^32,33^ (22.2% (95% CI 3.9% to 59.8%), p=.182).

Three studies clearly or potentially favored the intervention (30.0% (95% CI 8.1% to 64.6%), p=.343).

Three of 10 studies were judged to be high quality and, of these, none clearly favored the intervention and two potentially favored the intervention^32,33^.

For power of **digital marketing**, no study (of three) clearly favored or potentially favored the intervention. No studies were high quality, all were moderate.

For power of **product packaging**, one of three studies clearly favored the intervention^31^ (33.3% (95% CI 1.7% to 87.5%), p=1.00).

No studies were high quality, all were moderate.

- ***Marketing technique type***

Ten studies^2,4,12,15-17,32-34,36^ evaluated effects of any food marketing policy on the prevalence of promotional characters in food marketing, while five studies^8,11,24,31,35^ considered effects on the use of a range of child-appealing persuasive strategies and techniques (including promotional characters but also premium offers/gifts/prizes, games, toy/school references, images of animals/children, “fun/cool/hip” messages/colours/shapes, animation, and third party cross-promotions). One study^25^ evaluated the effect of a voluntary measure on use of animation as a production technique.

**Summary box. Comparison 1: Power results - subgroup analyses by marketing technique type**

For use of **promotional characters**, one study clearly favored the intervention^12^ (12.5% (95% CI 0.6% to 53.0%), p=.077).

Two studies potentially favored the intervention^32,33^ (22.2% (95% CI 3.9% to 59.8%), p=.182).

Three studies clearly or potentially favored the intervention (30.0% (95% CI 8.1% to 64.6%), p=.343).

Two of 10 studies were judged to be high quality and, of these, both potentially favored the intervention^32,33^.

For **child-appealing persuasive strategies**, one study clearly favored the intervention^31^ (20.0% (95% CI 1.1% to 70.1%), p=.371).

No study potentially favored the intervention.

One study clearly or potentially favored the intervention (20.0% (95% CI 1.1% to 70.1%), p=.371).

One of five studies was judged to be high quality and this study did not clearly favor or potentially favor the intervention.

For the **production technique of animation**, the single study identified (deemed to be moderate quality) did not clearly favor or potentially favor the intervention.

**Appendix E: Comparison 2: Mandatory policy vs no policy**

Ten studies reported effects of mandatory policies (versus no policy) on relevant outcomes of interest for this review.

Studies in this comparison all reported the effect of implementation of a mandatory policy compared with pre-policy^26-28,30-33,37-39^. Studies used a mix of observational designs namely: cross-sectional survey, repeated cross-sectional survey, repeated cross-sectional content analysis, and repeated cross-sectional content analysis and survey (Table 2).

Within this comparison of mandatory food marketing policies versus no policy, seven studies reported on exposure to food marketing, three studies reported on power of food marketing, two studies reported on food purchasing, one study reported on product change and three studies reported on unintended consequences.

More details about these studies are available in Table 1 (Key characteristics of n=44 included studies) and in Appendix B Tables S1-S6 (selected data extraction by outcome).

This section and the harvest plot below (Figure S1) provide an overview of effects on key outcomes.

**Critical outcomes**

1. **Exposure to food marketing**

Seven studies within this comparison reported on exposure, with effects ranging from clearly favoring the intervention in one study^28^ to potentially favoring the control in one study^27^. Four studies were judged to be high quality and three studies were moderate quality.

One repeated cross-sectional content analysis and survey study reported a clear effect favoring the intervention^28^. Preschoolers’ mean weekly minutes of exposure to child-directed ‘high in’ food marketing on television was significantly lower following implementation of the Chilean Government policy compared with before (1.3 ± 1.4 vs. 2.0 ± 2.4, p<0.001).

Four studies potentially favored the intervention^30-33^. One repeated cross-sectional content analysis narratively reported that GRPs for EDNP food advertisements during regulated hours was greater pre-policy (183) than post (0.8)^30^. Ofcom provided two repeated cross-sectional surveys. Ofcom (2008)^32^ narratively reported that HFSS food impacts for children aged 4-9 years were 39% lower post-policy compared with pre (3.7 billion vs. 6.1 billion) and Ofcom (2010)^33^ narratively reported that the same effect measure showed a 52% decrease in the later evaluation (from 5.9 billion to 2.8 billion). Pre-policy data were calculated slightly differently between the 2008 and 2010 reports which, it must be assumed, explains the difference in reported values for HFSS impacts in children aged 4-9 years (5.9 and 6.1). Notably, the post-policy arm of Ofcom (2008)^32^ reflects a point of partial implementation of the restrictions (where HFSS TV advertisements were banned in and around programming ‘of particular appeal’ to children aged 4-15 years (based on the proportion of child viewers in the audience) and dedicated children’s channels were required to have scaled back their HFSS advertising to 50% of their 2005 levels), whereas the post-policy arm of Ofcom (2010)^33^ reflects a point of full implementation, where there was a ban of HFSS advertising in and around programming appealing to children 4-15 years and there was also a full ban on HFSS advertising on dedicated children’s channels. One repeated cross-sectional content analysis narratively reported that the number and proportion of ‘high in’ cereal products in supermarkets was lower (86, 59%) following implementation of the Chile Government policy compared with pre-implementation (132, 78.6%)^31^.

One repeated cross-sectional survey reported no effect of a mandatory food marketing policy compared with no policy. The PMV for HFSS TV food advertising among child viewers aged 4-15 years did not change significantly pre- to post-UK Government policy (5174 vs. 7476; OR (99%CI) = 1.05 (0.99-1.12), p>0.05)^26^.

One repeated cross-sectional content analysis reported an unclear effect potentially favoring the control. The volume and rate (ads/hour/channel) of non-core TV food advertising on thematic channels for children (2-12 years) during peak viewing times for children was greater post-policy (203 or 6/hour/channel) compared with pre-policy (180 or 5/hour/channel)^27^.

**Summary box. Comparison 2: Exposure results.**

One study clearly favored the intervention^28^ (33.4% (95% CI 1.8% to 87.5%), p=1.00).

Four studies potentially favored the intervention^30-33^ (66.7% (95% CI 24.1% to 94.0%), p=.683).

Five studies clearly or potentially favored the intervention (71.4% (95% CI 30.3% to 94.9%), p=.449).

Four of seven studies were judged to be high quality, and, of these, none clearly favored the intervention and three potentially favored the intervention^30,32,33^.

***Subgroup analyses for exposure:***

- ***Definition of age of child in policy***

This subgroup analysis was not possible for this comparison because all mandatory policies evaluated define children as being > 12 years (see Table S7).

- ***Marketing medium***

Six studies^26-28,30,32,33^ evaluated the effect of mandatory food marketing policies on exposure to TV food advertising and one study considered effects of a mandatory policy on food marketing exposure via product packaging^31^.

**Summary box. Comparison 2: Exposure results - subgroup analyses by marketing medium**

For exposure to **TV advertising**, one study clearly favored the intervention^28^ (33.4% (95% CI 1.8% to 87.7%), p=1.00).

Three studies potentially favored the intervention^30,32,33^ (60.0% (95% CI 17.0% to 92.7%), p=1.00).

Four studies clearly or potentially favored the intervention (66.7% (95% CI 24.1% to 94.0%), p=.683).

Four of six studies were judged to be high quality, and, of these, none clearly favored the intervention and three potentially favored the intervention^30,32,33^.

For exposure to **product packaging**, the only study identified was moderate quality and potentially favored the intervention^31^.

- ***The approach to classifying foods to which the restrictions apply***

Three studies^26,32,33^ evaluated the UK Government policy (vs. no policy) that uses a nutrient profile model to categorize foods as HFSS (not permitted to be marketed to children) or non-HFSS (permitted to be marketed to children); three studies evaluated policies or measures that used uniform category-specific nutrition criteria^28,30,31^. A further study did not report on how foods were classified by the policy^27^.

**Summary box. Comparison 2: Exposure results - by approach used to classify foods to which the restrictions apply**

For studies of policies using a **nutrient profile model**, no studies clearly favored the intervention.

Two studies potentially favored the intervention^32,33^ (66.6% (95% CI 12.5% to 98.2%), p=1.00).

Two studies clearly or potentially favored the intervention (66.6% (95% CI 12.5% to 98.2%), p=1.00).

All three studies were judged to be high quality.

For studies of policies using **uniform** **category-specific nutritional criteria**, one study clearly favored the intervention^28^ (100.0% (95% CI 5.5% to 100%), p=1.00).

Two studies potentially favored the intervention^30,31^ (100% (95% CI 19.9% to 100%), p=.479).

Three studies clearly or potentially favored the intervention (100% (95% CI 31.0% to 100%), p=1.00).

One of three studies was judged to be high quality and it potentially favored the intervention^30^.

1. **Power of food marketing**

Three studies within this comparison reported on power, with effects either clearly favoring the intervention (one study^31^) or potentially favoring the intervention (two studies^32,33^). Two studies were judged to be high quality and one study was moderate quality.

One repeated cross-sectional content analysis reported that the proportion of ‘high in’ cereals using at least one child-directed marketing strategy was significantly lower post-policy compared with pre-policy (15.12% v 43.18%, p<0.05)^31^.

Two repeated cross-sectional survey studies narratively reported effects potentially favoring the intervention. One reported that the number of TV food advertising spots featuring licensed characters reduced by 56% pre-post policy (from 110,000 to 48,000 and from 4.1% of all food advertisements to 1.1%)^32^ and the other study reported that child impacts for TV food advertisements using a licensed character reduced 84% pre-post policy (from 0.39 billion to 0.06 billion)^33^.

**Summary box. Comparison 2: Power results.**

One study clearly favored the intervention^31^ (100% (95% CI 5.5% to 100%), p=1.00).

Two studies potentially favored the intervention^32,33^ (100% (95% CI 19.9% to 100%), p=.479).

Three studies clearly or potentially favored the intervention (100% (95% CI 31.0% to 100%), p=1.00).

Two of three studies were judged to be high quality, and, of these, none clearly favored the intervention and two potentially favored the intervention^32,33^.

***Subgroup analyses for power:***

- ***Definition of age of child in policy***

This subgroup analysis was not possible for this comparison because all mandatory policies evaluated define children as being > 12 years (see Table S7).

- ***Marketing medium***

Two studies evaluated the effect of mandatory food marketing policies on the power of TV advertising^32,33^ and one study^31^ considered effects of a mandatory food marketing policy on marketing power via product packaging.

**Summary box. Comparison 2: Power results - subgroup analyses by marketing medium**

For power of **TV advertising**, no studies clearly favored the intervention.

Two studies potentially favored the intervention^32,33^ (100.0% (95% CI 19.7% to 100%), p=.480).

Two studies clearly or potentially favored the intervention (100.0% (95% CI 19.7% to 100%), p=.480).

Two of two studies were judged to be high quality and, of these, none clearly favored the intervention and two potentially favored the intervention^32,33^.

For power of **product packaging**, the only study (moderate quality) clearly favored the intervention^31^.

- ***Marketing technique type***

Two studies^32,33^ evaluated the effect of food marketing policies or measures on the prevalence of promotional characters in food marketing, one study considered effects on the use of a range of child-appealing persuasive strategies and techniques (including promotional characters but also child-oriented gifts, games, toy/school references, child-words such as “for kids” and cross-promotions).

**Summary box. Comparison 2: Power results - subgroup analyses by marketing technique type**

For use of **promotional characters**, no studies clearly favored the intervention.

Two studies potentially favored the intervention^32,33^ (100.0% (95% CI 19.7% to 100%), p=.480).

Two studies clearly or potentially favored the intervention (100.0% (95% CI 19.7% to 100%), p=.480).

Two of two studies were judged to be high quality and, of these, both potentially favored the intervention^32,33^.

For **child-appealing persuasive strategies**, the only study (moderate quality) clearly favored the intervention^31^.

1. **Food preferences**

None of the included studies reported this outcome.

1. **Food choice**

None of the included studies reported this outcome.

1. **Food purchasing**

Two studies within this comparison reported on purchasing outcomes; one high quality study clearly favored the intervention^39^ and one moderate quality study clearly favored the control^38^.

One repeated cross-sectional survey reported effects clearly favoring the intervention^39^. The study found that in households with children, expenditure reduced by £6.2 per capita per quarter for HFSS foods and by £2.7 for HFSS drinks following implementation of the UK regulations, compared with pre-implementation.

One repeated cross-sectional survey reported effects clearly favoring the control^38^. The study, evaluating the San Francisco Healthy Foods Incentives Ordinance, reported that the number and proportion of children’s meals purchased increased significantly (123 (47.3%) vs. 88 (35.8%), p=0.01) following implementation of the policy compared to pre-implementation.

**Summary box. Comparison 2: Purchasing results.**

One study clearly favored the intervention^39^ (50.0% (95% CI 9.5% to 90.5%), p=1.00).

No studies potentially favored the intervention.

One study clearly or potentially favored the intervention (50.0% (95% CI 9.5% to 90.5%), p=1.00).

One of two studies was judged to be high quality, and this study clearly favored the intervention^39^.

1. **Dietary intake**

None of the included studies reported this outcome.

**Important outcomes**

1. **Product requests (“pester power”)**

None of the included studies reported this outcome.

1. **Dental caries/erosion**

None of the included studies reported this outcome.

1. **Body weight/body mass index/obesity**

None of the included studies reported this outcome.

1. **Diet-related non-communicable diseases (NCDs)**

None of the included studies reported this outcome.

1. **Product change**

One high quality study within this comparison reported on a product change outcome and found no effect^37^.

The cross-sectional survey reported that there was no significant difference in the average price of children’s brand breakfast cereals per 100g between Canadian provinces with no regulation (analysis conducted pre-CAI implementation) and Quebec, subject to the Quebec Consumer Protection Act ($0.87 vs. $0.89, p=0.08)^37^.

**Summary box. Comparison 2: Product change results.**

No studies clearly favored or potentially favored the intervention.

One study was deemed to be high quality.

1. **Unintended consequences**

Identical to comparison 1, three studies within this comparison reported on unintended consequences, one clearly favored the intervention^39^ and two potentially favored the intervention^30,32^. All three studies were deemed to be high quality.

One repeated cross-sectional survey reported that there was a 15.2 million decrease in TV HFSS advertising expenditure following implementation of the UK regulations, compared with pre-implementation^39^. This was a reduction of 19.4% and was statistically significant (p<0.01).

One repeated cross-sectional content analysis of the Republic of Korea policy narratively reported that the total advertising budget for energy-dense nutrient poor food promotion during regulated hours fell from $420,000 pre-policy to $2,000 post-policy^30^ and one repeated cross-sectional survey narratively reported that there was a 26% reduction in net food and drink advertising revenue on children’s channels pre- to post-UK Government policy implementation^32^.

**Summary box. Comparison 2: Unintended consequences results.**

One study clearly favored the intervention^39^ (100% (95% CI 5.4% to 100%), p=1.00).

Two studies potentially favored the intervention^30,32^ (100% (95% CI 19.8% to 100%), p=.480).

Three studies clearly or potentially favored the intervention^30,32^ (100% (95% CI 31.0% to 100%), p=.248).

All three studies were judged to be high quality.

**Figure S1. Harvest plot for Comparison 2**

**
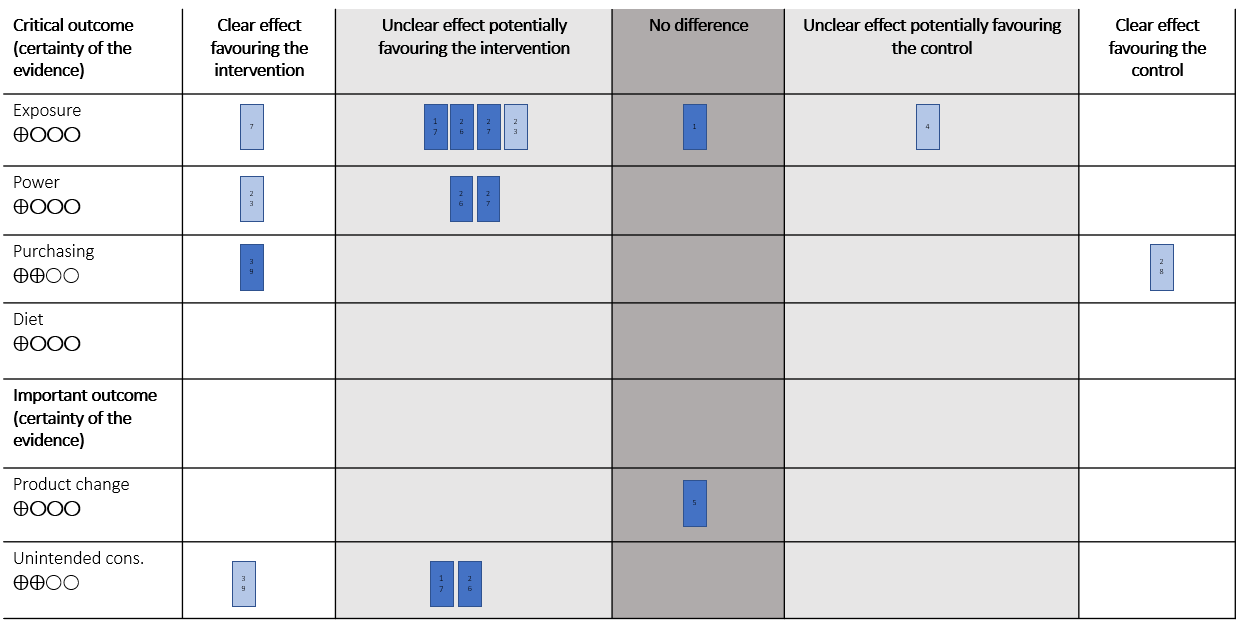
**Notes:

- Unintended cons. – Unintended consequences
- Each bar represents one study
- The number in each bar corresponds to the # number in Table 1
- Dark blue shading indicates a high quality study
- **Certainty of the evidence:** ⨁◯◯◯ very low, ⨁⨁◯◯ low, ⨁⨁⨁◯ moderate, ⨁⨁⨁⨁ high

**Appendix F: Comparison 3: Voluntary measures v no voluntary measure**

Twenty-nine studies reported effects of voluntary measures (versus no measure) on relevant outcomes of interest for this review.

Studies in this comparison mostly reported on the effect of implementation of a voluntary measure compared to before implementation of the measure (n=10 studies^1,3-5,9,14,17,21,22,25^) or effects between companies who were signatories versus non-signatories to voluntary measures (n=15 studies^2,6-8,13,15,16,18-20,23,24,34-36^). Four studies reported effect measures comparing both pre-post voluntary measures and between signatories and non-signatories^10-12,29^. Studies used a mix of observational designs namely: cross-sectional survey, repeated cross-sectional survey, cross-sectional content analysis, repeated cross-sectional content analysis designs or a combination of cross-sectional content analysis and survey or repeated cross-sectional content analysis and survey (see Table 2).

Within this comparison of voluntary measures versus no measure, 26 studies reported on exposure to food marketing, 13 studies reported on power of food marketing, 2 studies reported on food purchasing, 1 study reported on dietary intake, and 1 study reported on product change.

More details about these studies are available in Table 1 (Key characteristics of n=44 included studies) and in Appendix B Tables S1-S6 (selected data extraction by outcome).

This section and the harvest plot below (Figure S2) provide an overview of effects on key outcomes.

**Critical outcomes**

1. **Exposure to food marketing**

Twenty-six studies within this comparison arm reported on exposure, with effects ranging from clearly favoring the intervention in two studies^9,14^ to clearly favoring the control in three studies^4,16,18^. Studies were mostly moderate in quality (n = 18 studies) with seven high quality and one low quality^5^.

Studies reporting a clear effect favoring the intervention all reported desirable effects on exposure (i.e., reductions) as a result of the measure but through the use of different study designs and effect measures. Huang (2013)’s repeated cross-sectional survey study^9^ reported significantly reduced GRPs for confectionery (bubble gum) TV advertising post implementation of the CFBAI compared with pre-implementation. Lwin (2020)^14^ also used a repeated cross-sectional content analysis and survey design and reported significantly reduced proportions of unique advertisements that were for unhealthy foods (based on pre-defined product categories) on the TV channels with highest viewership (including one dedicated to child and youth audiences) following implementation of the Singapore food marketing policy compared with pre-implementation.

Six studies reported an unclear effect potentially favoring the intervention; they also reported potentially desirable effects of measures on exposure (narratively reported, no statistical testing) using different effect measures. Dembek (2012)^3^ and Frazier (2018)^5^, both repeated cross-sectional survey designs, found reductions in the average number of food TV advertisements viewed by children over a specified period (e.g., one year) pre- and post-implementation of the CFBAI voluntary measure. Hebden (2011)^29^ reported that a repeated cross-sectional content analysis and survey design found a reduction in the mean frequency of non-core fast food advertisements per year on the main free to air commercial TV channels pre- and post-implementation of the QSRI voluntary measure. Other repeated cross-sectional content analyses reported that the rate of non-core food advertisements (not including fast food) per hour per TV channel on the main free to air commercial TV channels reduced post-RCMI implementation compared with pre-implementation^10^ and the number and proportion of ‘high in’ cereals in major supermarkets reduced after implementation of the Chilean regulations^31^. The mean number of TV food advertisements viewed by children per day reduced post CFBAI implementation^22^ and proportion of TV food advertisements viewed by children that were for unhealthy foods reduced, again post vs. pre-CFBAI^21^, both studies used repeated cross-sectional survey designs.

Five studies reported no effect of food marketing measures, with effect measures annual national GRPs for carbonated soft drink advertising also in a repeated cross-sectional survey design^1^, average number of non-core food TV advertisements per hour on the main free to air commercial TV channels in a repeated cross-sectional content analysis^11^, unhealthy foods ads a proportion of all food advertisements around children’s TV programs on the most popular channels in repeated cross-sectional content analysis and repeated cross-sectional survey designs respectively^12,17^, and average nutrition scores of websites of brands commonly marketed to children in a cross-sectional content analysis^15^.

Ten studies reported an unclear effect potentially favoring the control, so potentially undesirable effects on exposure, again with variation in study design and effect measures. Brindal (2011)^2^ reported that a repeated cross-sectional survey found that AFGC RCMI signatory companies were responsible for a greater proportion of non-core food advertisements as a percentage of all food advertisements on the main free to air TV channels compared with non-signatories (78.3% v 23.5%). In three studies all using cross-sectional survey designs comparing CFBAI participating companies versus non-participating companies, it was reported that participating companies were responsible for a greater increase (percentage change over time) in number of confectionery advertisements viewed by children^8^ and greater volumes of food-related advertisements viewed on children’s TV^6,7^. One cross-sectional content analysis found a greater share of child-targeted food advertising (on children’s networks) by EU Pledge signatory companies compared with non-signatories^13^, a second cross-sectional content analysis found a greater number and proportion of ultra-processed foods advertised to children on highest rated TV channels by signatory companies of the Mexican self-regulatory measure compared with non-signatories^23^ and a third such content analysis found a greater proportion of products marketed to children on CAI signatory company websites that exceed 15% Daily Value for saturated fats, sodium and/or total sugars compared with non-signatory companies^24^. A repeated cross-sectional content analysis found a greater frequency of TV food advertisements during likely child viewing hours post-CFBAI implementation compared to pre-implementation^25^. One cross-sectional survey found a greater number and proportion of food advertisements for less healthy items around TV programs with a child audience share ≥35% being from CAI signatory versus non-signatory companies^19^ and another cross-sectional survey found a greater proportion of food or beverage product ads that are ‘high in nutrients to limit’ during children’s programming by CFBAI signatories versus non-signatories^20^.

Three studies clearly favored the control, reporting undesirable effects (increased exposure to food marketing) including a repeated cross-sectional content analysis that found significantly increased proportions of non-core food advertisements on TV channels popular with children and adolescents following implementation of the EU Pledge^4^. Two Canadian studies evaluated the marketing by CAI signatory companies versus non-signatories. One, using cross-sectional content analysis and survey, found a significantly greater number and proportion of less healthy food advertisements by signatory companies during children’s preferred television^16^ and a cross-sectional content analysis found that the likelihood of food marketing being ‘less healthy’ was significantly greater on the websites of signatory companies compared with non-signatories^18^.

**Summary box. Comparison 3: Exposure results.**

Two studies clearly favored the intervention^9,14^ (10.0% (95% CI 1.8% to 33.1%), p<.001).

Six studies potentially favored the intervention^3,5,10,21,22,29^ (25% (95% CI 10.6% to 47.1%), p=.025).

Eight studies clearly or potentially favored the intervention (30.8% (95% CI 15.1% to 51.9%), p=.078).

Seven of 26 studies were judged to be high quality, and, of these, one clearly favored the intervention^9^ and two potentially favored the intervention^21,22^.

***Subgroup analyses for exposure:***

- ***Definition of age of child in policy***

Twenty-five studies^1-25^ evaluated food marketing measures (vs. no policy or measure) in which the definition of the age of a child in the policy was 12 years or under (and therefore the policy aimed to restrict food marketing to this age group), and 1 study^29^ evaluated a voluntary measure (vs. no measure) in which the definition of the age of a child included children over 12 years (see Table S7 for specific definitions of age of children for each policy).

**Summary box. Comparison 3: Exposure results - subgroup analyses by age of child in policy**

For studies of measures where children were defined as **12 years or under**, two studies clearly favored the intervention^9,14^ (10% (95% CI 1.8% to 33.1%), p<.001).

Five studies potentially favored the intervention^3,5,10,21,22^ (21.7% (95% CI 8.3% to 44.2%), p=.012).

Seven studies clearly or potentially favored the intervention (28.0% (95% CI 12.9% to 50.0%), p=.046).

Seven of 25 studies were judged to be high quality and, of these, one clearly favored the intervention^9^ and two potentially favored the intervention^21,22^.

For studies of measures in which the definition of the age of a child included children **over 12 years**, no study clearly favored the intervention.

One study potentially favored the intervention^29^ (100.0% (95% CI 5.5% to 100%), p=1.00).

One study clearly or potentially favored the intervention (100.0% (95% CI 5.5% to 100%), p=1.00).

No study was judged to be high quality.

As per comparison 1, some studies reported relevant exposure effect measures for two age groups and only one effect measure could be included in the above evidence synthesis^3,5-7,20-22^. Importantly, however, where both age groups were captured by the definition of children in the measure being evaluated, results were consistent across the two effect measures. For example, Powell (2010)^22^ and Powell (2011)^21^ reported potentially desirable effects of the measure (child defined as <12 years) on exposure in both 2-5 and 6-11 year old children. Notably, the two effect measures in Powell (2013)^20^ were also consistent but in the opposite direction (potentially undesirable effects on exposure for both 2-5 and 6-11 year old children). This may reflect differences in study design as Powell (2013)^20^ was a cross-sectional survey comparing signatories and non-signatories to a voluntary measure (CFBAI) whereas the earlier studies used repeated cross-sectional (pre-post implementation) designs^21,22^. Harris (2018)^7^ also reported potentially undesirable effects of the measure (child defined as < 12 years) on both 2-5 and 6-11 year old children.

Notably, three studies reported effects of the measure on children both within and outside the policy definition of a child^3,5,6^. Harris (2017)^6^ reported potentially undesirable effects of the measure (child defined as <12 years) on exposure in both 2-6 years old children (age captured by the measure) and young teenagers (12-14 years; not captured by the measure), whereas Frazier (2018)^5^ reported potentially desirable effects of the measure (child defined as <12 years) on exposure in both 2-11 year old (age captured by the measure) and 12-17 year old children (not captured by the measure). Conversely, Dembek (2012)^3^ reported potentially desirable effects of the measure (child defined as <12 years) on exposure in 2-11 year old children but concurrent potentially undesirable effects on exposure in 12-17 year old children (age not captured by the measure).

- ***Marketing medium***

As reflects the advertising medium most commonly restricted by the policies evaluated in the included studies, the majority of studies in this comparison (n=23) evaluated the effect of food marketing policies or measures on exposure to TV food advertising^1-14,16,17,19-23,25,29^. Three studies^15,18,24^ considered effects of voluntary measures on digital food marketing exposure (all websites).

**Summary box. Comparison 3: Exposure results - subgroup analyses by marketing medium**

For exposure to **TV advertising**, two studies clearly favored the intervention^9,14^ (11.8% (95% CI 2.1% to 37.7%), p=.003).

Six studies potentially favored the intervention^3,5,10,21,22,29^ (28.6% (95% CI 12.2% to 52.3%), p=.081).

Eight studies clearly or potentially favored the intervention (34.8% (95% CI 17.2% to 57.2%), p=.211).

Seven of 23 studies were judged to be high quality and, of these, one clearly favored the intervention^9^ and two potentially favored the intervention^21,22^.

For exposure to **digital marketing**, no study (of three) clearly favored or potentially favored the intervention, all were moderate quality.

- ***The approach to classifying foods to which the restrictions apply***

Fourteen studies evaluated voluntary measures that used company specific nutrition thresholds^1,2,4,9-13,16,17,20-22,25^, and 11 studies evaluated measures that used uniform category-specific nutrition criteria^3,5-8,14,15,18,19,24,29^. One study did not report on how foods were classified by the policy^23^.

**Summary box. Comparison 3: Exposure results – subgroup analyses by approach used to classify foods to which the restrictions apply**

For studies of measures using **company specific nutritional criteria**, one study clearly favored the intervention^9^ (9.1% (95% CI 0.4% to 42.9%), p=.015).

Three studies potentially favored the intervention^10,21,22^ (23.1% (95% CI 6.1% to 54.0%), p=.096).

Four studies clearly or potentially favored the intervention (28.6% (95% CI 9.6% to 58.0%), p=.181).

Four of 14 studies were judged to be high quality and, of these, one clearly favored the intervention^9^ and two potentially favored the intervention^21,22^.

For studies of policies or measures using **uniform** **category-specific nutritional criteria**, one study clearly favored the intervention^14^ (12.5% (95% CI 0.7% to 53.3%), p=.077).

Three studies potentially favored the intervention^3,5,29^ (30.0% (95% CI 8.1% to 64.6%), p=.342).

Four studies clearly or potentially favored the intervention (36.4% (95% CI 12.4% to 68.4%), p=.546).

Three of 11 studies were judged to be high quality and, of these, none clearly favored or potentially favored the intervention.

1. **Power of food marketing**

Thirteen studies within this comparison arm reported on power outcomes, with effects ranging from clearly favoring the intervention in one study^12^ to clearly favoring the control in five studies^4,15,16,25,34^. Studies were mostly moderate in quality (n = 12 studies) with one high quality^8^.

One study reported a clear effect favoring the intervention, with a desirable effect on power of food marketing (i.e., a reduction) as a result of the measure. The study used a repeated cross-sectional content analysis design. Kunkel (2015)^12^ reported that a significantly lower proportion of TV advertisements for unhealthy foods (‘whoa’ foods, as a proportion of all food advertisements) featured a licensed character following implementation of the measure (CFBAI, see Table S7) compared with before implementation (61.0% vs. 91.4%).

One study found no effect; Potvin Kent (2013)’s cross-sectional content analysis^36^ found no significant difference (p=0.568) in their sample between the number and proportion of food brand websites with spokes characters between CAI signatory (8, 57.1%) and non-signatory companies (6, 46.2%).

Six studies reported an unclear effect potentially favoring the control, i.e., potentially undesirable effects on power as a result of measures, again with variation in study designs and effect measures. Brindal (2011)^2^ reported on a repeated cross-sectional survey that found that AFGC RCMI signatory companies were responsible for a greater number (and proportion) of non-core TV food advertising using promotional characters (6, 2.9%) than non-signatory companies (0, 0%) and King (2011)’s repeated cross-sectional content analysis^11^ also found RCMI signatories to have broadcast a greater number and proportion of TV food advertisements using persuasive techniques (112, 62%) compared with non-signatories (69, 38%). One cross-sectional survey found that CFBAI participating companies were responsible for a greater number (and proportion) of candy advertisements featuring child-targeted techniques viewed by children (2-11 years) compared with non-participating companies (287 (59%) vs. 107 (22%)^8^ and another cross-sectional survey reported that the number of different child-oriented features on high sugar cereal packaging was greater for CFBAI participating companies (mean 3.6) compared with non-participating companies (mean 1.4)^35^. In a repeated cross-sectional survey study, Potvin Kent (2014b)^17^ reported a 234% increase in the number of less healthy FNAB TV advertisements following implementation of the CAI compared with pre-implementation, and Vergeer (2019)’s cross-sectional content analysis^24^ found that 93.3% of CAI signatory companies had child-directed marketing featuring marketing techniques on their website compared to just 6.7% of non-signatories.

Five studies clearly favored the control, reporting undesirable effects of the policy/measure (i.e., increased power of food marketing). Effertz (2012)^4^ reported on a repeated cross-sectional content analysis that found a significant increase (OR 4.188, p<0.001) in the propensity for non-core food TV advertisements to contain a promotional character following implementation of the EU Pledge compared with pre-implementation, and Neyens (2017)^15^ similarly reported a significantly greater presence of spokes characters on the websites of EU Pledge signatory companies compared with non-signatories using a cross-sectional content analysis design (mean ranks 31.93 vs. 21.94, p=0.017). Galloway (2014)^34^ reported on a repeated cross-sectional content analysis that found the nutritional quality of food products (proportion of ‘whoa’ products) with media characters was significantly greater for CFBAI signatory companies compared with non-signatories (80.5% vs. 35%, p<0.0001). Potvin Kent (2011b)’s cross-sectional content analysis and survey^16^ found that the number (and proportion) of TV food advertisements featuring media characters that were for less healthy foods was significantly higher for CAI signatory (72, 92%) than non-signatories (9, 45%) and Warren (2007)^25^ found that the proportion of TV food advertisements featuring the production technique of animation was 10% higher following CFBAI implementation compared with pre-implementation (35.7% vs. 25.9%, p<0.001) using a repeated cross-sectional content analysis design.

**Summary box. Comparison 3: Power results.**

One study clearly favored the intervention^12^ (7.7% (95% CI 0.4% to 37.9%), p=.005).

No studies potentially favored the intervention.

One study clearly or potentially favored the intervention (7.7% (95% CI 0.4% to 37.9%), p=.005).

One of 13 studies was judged to be high quality, and it did not clearly favor or potentially favor the intervention^8^.

***Subgroup analyses for power:***

- ***Definition of age of child in policy***

This subgroup analysis was not possible for this comparison because all voluntary measures evaluated here define children as < 12 years (see Table S7).

- ***Marketing medium***

Eight studies evaluated the effect of voluntary measures on the power of TV food advertising^2,4,8,11,12,16,17,25^, three studies^15,24,36^ considered effects of voluntary measures on digital food marketing power (all websites) and two studies^34,35^ considered effects of voluntary measures on marketing power via product packaging.

**Summary box. Comparison 3: Power results - subgroup analyses by marketing medium**

For power of **TV advertising**, one study clearly favored the intervention^12^ (12.5% (95% CI 0.7% to 53.3%), p=.077).

No studies potentially favored the intervention.

One study clearly or potentially favored the intervention (12.5% (95% CI 0.7% to 53.3%), p=.077).

One of 8 studies was judged to be high quality and this did not clearly favor or potentially favor the intervention.

For power of **digital marketing**, no study (of three) clearly favored or potentially favored the intervention. No studies were high quality, all were moderate.

For power of **product packaging**, no study (of two) clearly favored or potentially favored the intervention. No studies were high quality, both were moderate.

- ***Marketing technique type***

Eight studies^2,4,12,15-17,34,36^ evaluated the effect of measures on the prevalence of promotional characters in food marketing, while four studies^8,11,24,35^ considered effects on the use of a range of child-appealing persuasive strategies and techniques (including promotional characters but also premium offers/gifts/prizes, games, toy/school references, images of animals/children, “fun/cool/hip” messages/colours/shapes, animation, and third party cross-promotions). One study^25^ evaluated the effect of a voluntary measure on use of animation as a production technique.

**Summary box. Comparison 3: Power results - subgroup analyses by marketing technique type**

For use of **promotional characters**, one study clearly favored the intervention^12^ (12.5% (95% CI 0.7% to 53.3%), p=.077).

No studies potentially favored the intervention.

One study clearly or potentially favored the intervention (12.5% (95% CI 0.7% to 53.3%), p=.077).

No studies were judged to be high quality.

For **child-appealing persuasive strategies**, no studies clearly favored or potentially favored the intervention.

One of four studies was judged to be high quality and this study did not clearly favor or potentially favor the intervention.

For the **production technique of animation**, the single study identified (deemed to be moderate quality) did not clearly favor or potentially favor the intervention.

1. **Food preferences**

None of the included studies reported this outcome.

1. **Food choice**

None of the included studies reported this outcome.

1. **Food purchasing**

Two studies within this comparison reported on purchasing outcomes; both clearly favored the intervention^9,14^. One study was high quality, and one study was of moderate quality.

Evidence from one repeated cross-sectional survey^9^ found that that relative purchase frequency for a confectionery item (bubble gum) decreased significantly (-2.486% ± 0.684, p<0.01) in households with children (<12y) pre-post implementation of the CFBAI (see Table S7). Another repeated cross-sectional survey found that in households with children, expenditure reduced by £6.2 per capita per quarter for HFSS foods and by £2.7 for HFSS drinks following implementation of the UK regulations, compared with pre-implementation^39^. One repeated cross-sectional content analysis and survey found, via a home food inventory checklist, that post-implementation of the SCAP (relative to pre-), the overall amount of unhealthy food in household pantries reduced from 721.65 (± 806.94) to 526.16 (± 736.11; p=0.001)^14^.

**Summary box. Comparison 3: Purchasing results.**

Two studies clearly favored the intervention^9,14^ (100.0% (95% CI 19.8% to 100%), p=.480).

No studies potentially favored the intervention.

Two studies clearly or potentially favored the intervention (100.0% (95% CI 19.8% to 100%), p=.480).

One of two studies was judged to be high quality, and this clearly favored the intervention^9^.

1. **Dietary intake**

One moderate quality study within this comparison reported on dietary intake outcomes; it clearly favored the intervention^14^.

The repeated cross-sectional content analysis and survey reported that the self-reported consumption score of children (9-16 years) for potato chips was significantly lower post-implementation of the SCAP compared with pre-policy (1.91 ± 0.69 vs. 1.97 ± 0.72, p=0.03) an effect reported to be driven by the 13-16 year old’s in the sample as no significant difference was found for those 12 years and below.

**Summary box. Comparison 3: Dietary intake results.**

One study clearly favored the intervention^14^ (100.0% (95% CI 5.5% to 100%), p=1.00).

One study clearly or potentially favored the intervention (100.0% (95% CI 5.5% to 100%), p=1.00).

No study potentially favored the intervention or was deemed to be high quality.

**Important outcomes**

1. **Product requests (“pester power”)**

None of the included studies reported this outcome.

1. **Dental caries/erosion**

None of the included studies reported this outcome.

1. **Body weight/body mass index/obesity**

None of the included studies reported this outcome.

1. **Diet-related non-communicable diseases (NCDs)**

None of the included studies reported this outcome.

1. **Product change**

One moderate quality study within this comparison reported on a product change outcome; it clearly favored the control^35^.

One cross-sectional survey^35^ evaluated the sugar content of breakfast cereals between signatory and non-signatory companies of the CFBAI, finding that mean sugar content was significantly higher for signatories (8.7g ± 2.22 vs. 6.6 ± 3.44, p<0.05).

**Summary box. Comparison 3: Product change results.**

No studies clearly favored or potentially favored the intervention.

No study was deemed to be high quality.

1. **Unintended consequences**

None of the included studies reported this outcome.

**Figure S2. Harvest plot for Comparison 3**

**
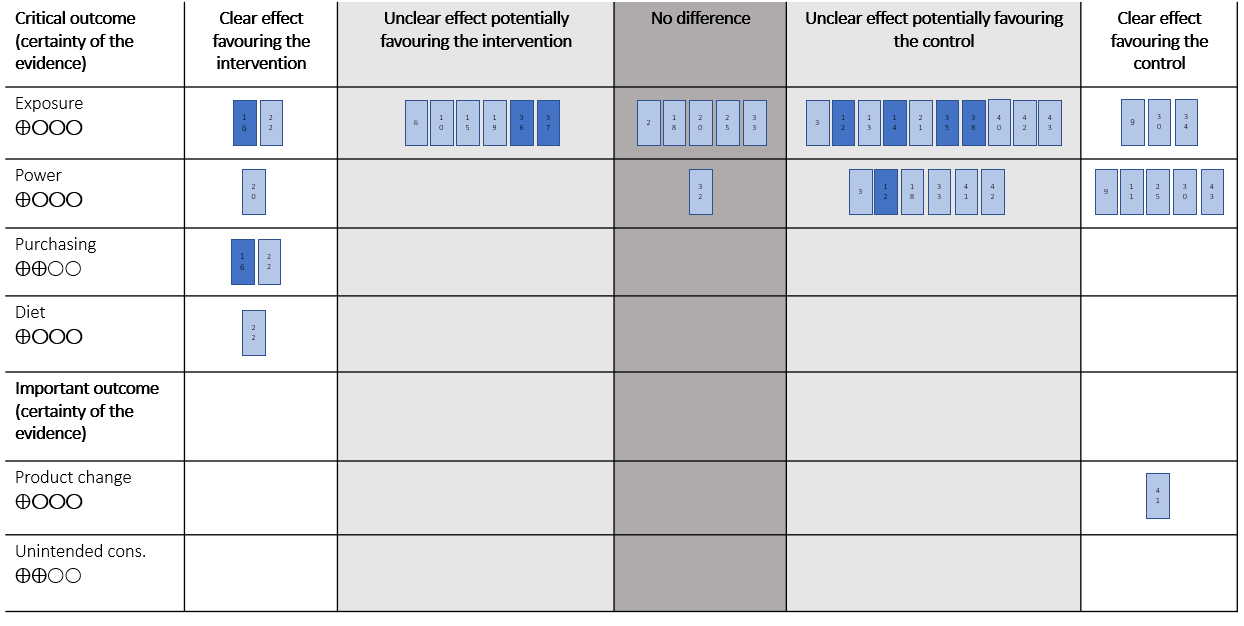
**

Notes:

- Unintended cons. – Unintended consequences
- Each bar represents one study
- The number in each bar corresponds to the # number in Table 1
- Dark blue shading indicates a high quality study
- **Certainty of the evidence:** ⨁◯◯◯ very low, ⨁⨁◯◯ low, ⨁⨁⨁◯ moderate, ⨁⨁⨁⨁ high

**Appendix G: Comparison 4: Mandatory policy vs voluntary measures**

Four studies reported effects of mandatory policies versus voluntary measures on relevant outcomes of interest for this review.

Studies in this comparison evaluated the effect of the Australian Broadcasting Authority's Children's Television Standards (mandatory policy) compared with the voluntary Commercial Television Industry Code of Practice^40^ or the effect of the Quebec Consumer Protection Act (mandatory) compared with the CAI (voluntary)^41-43^.

Study designs used were cross-sectional content analysis^40^, cross-sectional content analysis and survey^42^, repeated cross-sectional content analysis and survey^41^ and a natural experiment^43^ (see Table 2).

Within this comparison of mandatory policies versus voluntary measures, three studies reported on exposure to food marketing, two studies reported on power of food marketing, and one study reported on food purchasing.

More details about these studies are available in Table 1 (Key characteristics of n=44 included studies) and in Appendix B Tables S1-S6 (selected data extraction by outcome).

This section and the harvest plot below (Figure S3) provide an overview of effects on key outcomes.

**Critical outcomes**

1. **Exposure to food marketing**

Three studies within this comparison reported on exposure, with effects ranging from clearly favoring the intervention in one study^42^ to clearly favoring the control in one study^40^. Studies were all moderate quality.

One cross-sectional content analysis and survey study reported a clear effect favoring the intervention^42^. The proportion of TV food advertisements that were for ‘less healthy’ items was significantly lower during the preferred viewing of Quebec French children (regulated by the Quebec Consumer Protection Act) compared with that of Ontario English children (regulated by the CAI; 60.6% vs. 68.3%, p<0.001).

Another similar evaluation was conducted by Potvin Kent (2011a)^41^ using a repeated cross-sectional content analysis and survey and reported no effect. There was no significant difference between the number of TV food advertisements (as a percentage of all advertisements) between the preferred viewing of Quebec French children (regulated by the Quebec Consumer Protection Act) compared with that of Ontario English children (regulated by the CAI; 119 (26.6%) vs. 117 (23.5%), p=0.06).

A cross-sectional content analysis comparing the Australian Broadcasting Authority's Children's Television Standards (Government-led regulation) with the voluntary Commercial Television Industry Code of Practice reported a clear effect favoring the control^40^. The prevalence of TV food advertisements (as a proportion of all advertisements) on children’s channels (regulated by the mandatory policy) was significantly greater (41.1%) than on the general channels (regulated by the voluntary measure; 30.1%, p<0.001).

**Summary box. Comparison 4: Exposure results.**

One study clearly favored the intervention^42^ (33.3% (95% CI 1.8% to 87.5%), p=1.00).

No study potentially favored the intervention.

One study clearly or potentially favored the intervention (33.3% (95% CI 1.8% to 87.5%), p=1.00).

No studies were judged to be high quality.

1. **Power of food marketing**

Two studies within this comparison arm reported on power, with effects ranging from clearly favoring the intervention in one study^42^ to clearly favoring the control in one study^40^. Studies were both moderate quality.

One repeated cross-sectional content analysis and survey reported a clear effect favoring the intervention^41^. The number and proportion of TV food advertisements featuring a media character or celebrity was significantly lower during the preferred viewing of Quebec French children (regulated by the Quebec Consumer Protection Act) compared with that of Ontario English children (regulated by the CAI; 14 (11.7%) vs. 28 (23.9%), p<0.04).

One cross-sectional content analysis comparing the Australian Broadcasting Authority's Children's Television Standards (mandatory regulation) with the voluntary Commercial Television Industry Code of Practice reported a clear effect favoring the control^40^. The proportion of TV food advertisements featuring premium offers was significantly greater on children’s channels (regulated by the Government policy) compared with the general channels (regulated by the voluntary measure; 36% v 17%, p<0.0001).

**Summary box. Comparison 4: Power results.**

One study clearly favored the intervention (50.0% (95% CI 9.5% to 90.5%), p=1.00).

No study (of two) potentially favored the intervention.

One study clearly or potentially favored the intervention (50.0% (95% CI 9.5% to 90.5%), p=1.00).

No studies were judged to be high quality.

1. **Food preferences**

None of the included studies reported this outcome.

1. **Food choice**

None of the included studies reported this outcome.

1. **Food purchasing**

One study within this comparison reported on purchasing, reporting a clear effect favoring the intervention^43^.

One natural experiment evaluated change in fast food purchase propensity over time between Francophone (FP) and Anglophone (AP) households with children in Quebec compared with those in Ontario^43^. The authors report that the rationale underpinning the study design is that FP households primarily consume media from Quebec-based French-language media sources (regulated by the Quebec Consumer Protection Act) whereas AP households tend to consume English-language media from outside the province (regulated by the CAI). In Quebec, FP households had a significantly lower propensity to consume fast food (13.4%, p<0.01) than AP households but there were no differences between FP and AP households in Ontario. The difference in difference estimate implies that the Quebec ban led to a decrease in purchase propensity by 10.2% (p<0.05).

**Summary box. Comparison 4: Purchasing results.**

One study clearly favored the intervention (100.0% (95% CI 5.5% to 100%), p=1.00).

No study potentially favored the intervention.

One study clearly or potentially favored the intervention (100.0% (95% CI 5.5% to 100%), p=1.00).

The study was high quality.

1. **Dietary intake**

None of the included studies reported this outcome.

**Important outcomes**

1. **Product requests (“pester power”)**

None of the included studies reported this outcome.

1. **Dental caries/erosion**

None of the included studies reported this outcome.

1. **Body weight/body mass index/obesity**

None of the included studies reported this outcome.

1. **Diet-related non-communicable diseases (NCDs)**

None of the included studies reported this outcome.

1. **Product change**

None of the included studies reported this outcome.

1. **Unintended consequences**

None of the included studies reported this outcome.

**Figure S3. Harvest plot for Comparison 4**

**
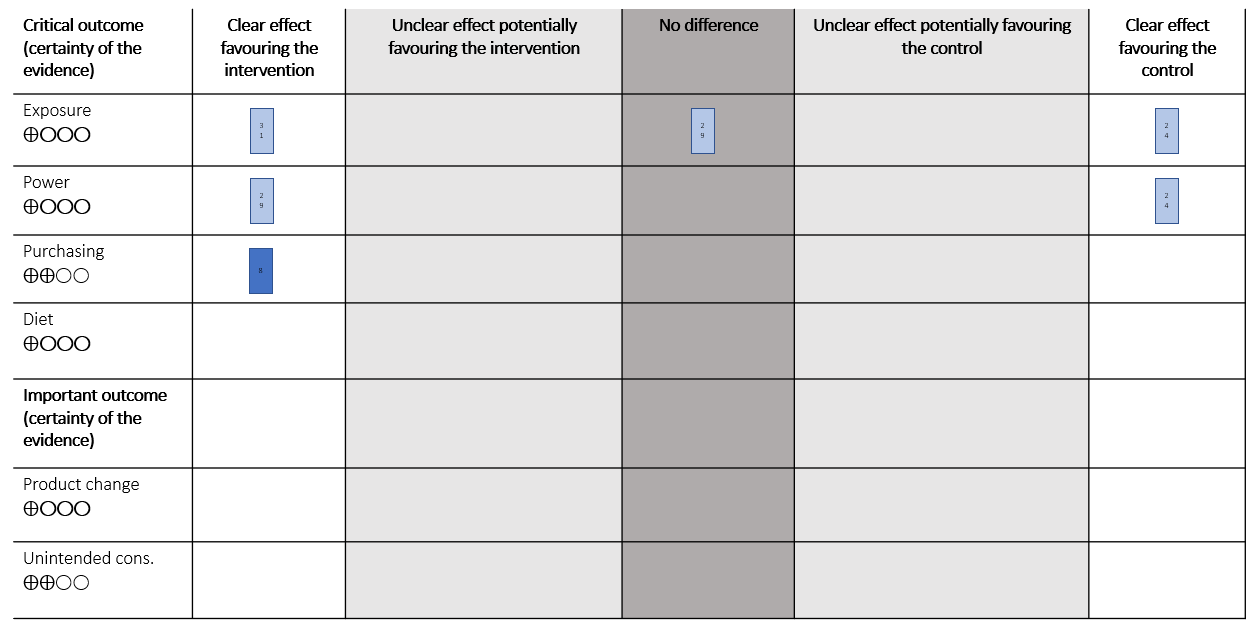
**

Notes:

- Unintended cons. – Unintended consequences
- Each bar represents one study
- The number in each bar corresponds to the # number in Table 1
- Dark blue shading indicates a high quality study
- **Certainty of the evidence:** ⨁◯◯◯ very low, ⨁⨁◯◯ low, ⨁⨁⨁◯ moderate, ⨁⨁⨁⨁ high

**Appendix H: Comparison 5: Mandatory policy (full implementation) vs mandatory policy (partial implementation)**

One study reported effects of a mandatory policy (full implementation) vs a mandatory policy (partial implementation) on a relevant outcome of interest for this review^44^.

The study reported on the effect of full implementation of the UK restrictions compared with partial implementation on exposure to food marketing using a repeated cross-sectional content analysis design (Whalen 2017). At the point of partial implementation of the restrictions, HFSS TV advertisements were banned in and around programming ‘of particular appeal’ to children aged 4-15 years (based on the proportion of child viewers in the audience) and dedicated children’s channels were required to have scaled back their HFSS advertising to 50% of their 2005 levels. Following full implementation, the ban of HFSS advertising in and around programming appealing to children 4-15 years was retained, and there was also a full ban on HFSS advertising on dedicated channels.

More details about this study are available in Table 1 (Key characteristics of n=44 included studies) and in Appendix B Tables S1-S6 (selected data extraction by outcome).

This section and the harvest plot below (Figure S4) provide an overview of effects on key outcomes.

**Critical outcomes**

1. **Exposure to food marketing**

One repeated cross-sectional content analysis of moderate quality reported on exposure, and potentially favored the intervention. The study narratively reported a reduction in TV advertisements for non-core (unhealthy) foods (as a proportion of all food advertisements) with mandatory policy (full implementation of restrictions) compared with a partial implementation of restrictions; 51.2% vs. 59.8%^44^.

**Summary box. Comparison 5: Exposure results.**

No study clearly favored the intervention.

One study potentially favored the intervention^44^ (100.0% (95% CI 5.5% to 100%), p=1.00).

One study clearly or potentially favored the intervention (100.0% (95% CI 5.5% to 100%), p=1.00).

The study was judged to be moderate quality.

1. **Power of food marketing**

None of the included studies reported this outcome.

1. **Food preferences**

None of the included studies reported this outcome.

1. **Food choice**

None of the included studies reported this outcome.

1. **Food purchasing**

None of the included studies reported this outcome.

1. **Dietary intake**

None of the included studies reported this outcome.

**Important outcomes**

1. **Product requests (“pester power”)**

None of the included studies reported this outcome.

1. **Dental caries/erosion**

None of the included studies reported this outcome.

1. **Body weight/body mass index/obesity**

None of the included studies reported this outcome.

1. **Diet-related non-communicable diseases (NCDs)**

None of the included studies reported this outcome.

1. **Product change**

None of the included studies reported this outcome.

1. **Unintended consequences**

None of the included studies reported this outcome.

**Figure S4. Harvest plot for Comparison 5**

**
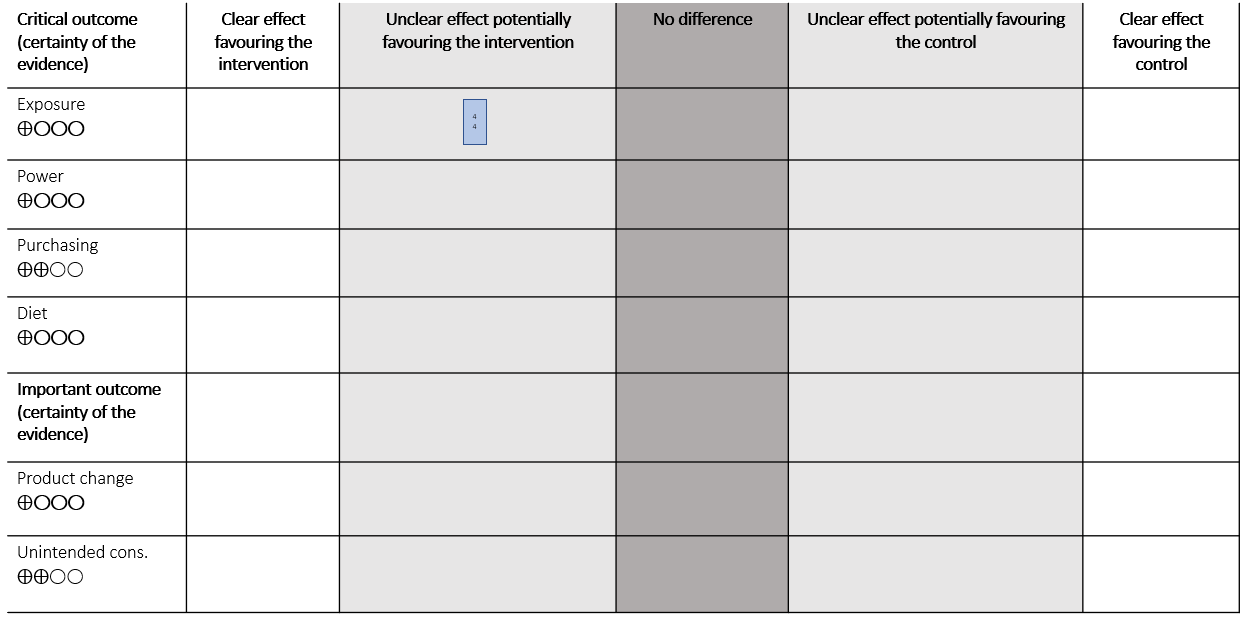
**

Notes:

- Unintended cons. – Unintended consequences
- Each bar represents one study
- The number in each bar corresponds to the # number in Table 1
- Dark blue shading indicates a high quality study
- **Certainty of the evidence:** ⨁◯◯◯ very low, ⨁⨁◯◯ low, ⨁⨁⨁◯ moderate, ⨁⨁⨁⨁ high

Included study

References

1. Berning J, McCullough M. Advertising Soft Drinks to Children: Are Voluntary Restrictions Effective? *Agribusiness* 2013; **29**(4): 469-85.

2. Brindal E, Corsini N, Hendrie G. Television food advertising to children in South Australia. South Australia: CSIRO. 2011.

3. Dembek C, Harris JL, Schwartz MB. Trends in television food advertising to young people: 2011 update. Yale Rudd Center for Food Policy and Obesity. 2012.

4. Effertz T, Wilcke A. Do television food commercials target children in Germany? *Public Health Nutrition* 2012; **15**(8): 1466-73.

5. Frazier W, Harris JL. Trends in Television Food Advertising to Young People: 2017 Update. UConn Rudd Center for Food Policy and Obesity. 2018.

6. Harris JL, Frazier W, Romo-Palafox M, et al. FACTS 2017 Food industry self-regulation after 10 years: Progress and opportunities to improve food advertising to children. UConn Rudd Center for Food Policy and Obesity. 2017.

7. Harris JL, Kalnova SS. Food and beverage TV advertising to young children: Measuring exposure and potential impact. *Appetite* 2018; **123**: 49-55.

8. Harris JL, LoDolce M, Dembek C, Schwartz MB. Sweet promises: Candy advertising to children and implications for industry self-regulation. *Appetite* 2015; **95**: 585-92.

9. Huang R, Yang M. Buy What is Advertised on Television? Evidence from Bans on Child-Directed Food Advertising. *Journal of Public Policy & Marketing* 2013; **32**(2): 207-22.

10. King L, Hebden L, Grunseit A, Kelly B, Chapman K. Building the case for independent monitoring of food advertising on Australian television. *Public Health Nutrition* 2013; **16**(12): 2249-54.

11. King L, Hebden L, Grunseit A, Kelly B, Chapman K, Venugopal K. Industry self regulation of television food advertising: responsible or responsive? *International Journal of Pediatric Obesity* 2011; **6**((2-2)): e390-8.

12. Kunkel DL, Castonguay JS, Filer CR. Evaluating Industry Self-Regulation of Food Marketing to Children. *Am J Prev Med* 2015; **49**(2): 181-7.

13. Landwehr SC, Hartmann M. Industry self-regulation of food advertisement to children: Compliance versus effectiveness of the EU Pledge. *Food Policy* 2020; **91**: 101833.

14. Lwin MO, Yee AZH, Lau J, et al. A macro-level assessment of introducing children food advertising restrictions on children’s unhealthy food cognitions and behaviors. *International Journal of Advertising* 2020: 1-22.

15. Neyens E, Smits T. Empty pledges: a content analysis of Belgian and Dutch child-targeting food websites. *International Journal of Health Promotion and Education* 2017; **55**(1): 42-52.

16. Potvin Kent M, Dubois L, Wanless A. Self-regulation by industry of food marketing is having little impact during children's preferred television. *International Journal of Pediatric Obesity* 2011b; **6**(5-6): 401-8.

17. Potvin Kent M, Martin CL, Kent EA. Changes in the volume, power and nutritional quality of foods marketed to children on television in Canada. *Obesity (Silver Spring)* 2014b; **22**(9): 2053-60.

18. Potvin Kent M, Pauzé E. The effectiveness of self-regulation in limiting the advertising of unhealthy foods and beverages on children's preferred websites in Canada. *Public Health Nutr* 2018a; **21**(9): 1608-17.

19. Potvin Kent M, Smith JR, Pauzé E, L'Abbé M. The effectiveness of the food and beverage industry's self-established uniform nutrition criteria at improving the healthfulness of food advertising viewed by Canadian children on television. *Int J Behav Nutr Phys Act* 2018b; **15**(1): 57.

20. Powell LM, Schermbeck RM, Chaloupka FJ. Nutritional content of food and beverage products in television advertisements seen on children's programming. *Child Obes* 2013; **9**(6): 524-31.

21. Powell LM, Schermbeck RM, Szczypka G, Chaloupka FJ, Braunschweig CL. Trends in the nutritional content of television food advertisements seen by children in the United States: analyses by age, food categories, and companies. *Arch Pediatr Adolesc Med* 2011; **165**(12): 1078-86.

22. Powell LM, Szczypka G, Chaloupka FJ. Trends in exposure to television food advertisements among children and adolescents in the United States. *Arch Pediatr Adolesc Med* 2010; **164**(9): 794-802.

23. Théodore FL, Tolentino-Mayo L, Hernández-Zenil E, et al. Pitfalls of the self-regulation of advertisements directed at children on Mexican television. *Pediatric Obesity* 2017; **12**(4): 312-9.

24. Vergeer L, Vanderlee L, Potvin Kent M, Mulligan C, L'Abbé MR. The effectiveness of voluntary policies and commitments in restricting unhealthy food marketing to Canadian children on food company websites. *Applied Physiology, Nutrition & Metabolism* 2019; **44**(1): 74-82.

25. Warren R, Wicks JL, Wicks RH, Fosu I, Donghung C. Food and beverage advertising to children on U.S. television: did national food advertisers respond? . *Journalism & Mass Communication Quarterly* 2007; **84**.

26. Adams J, Tyrrell R, Adamson AJ, White M. Effect of Restrictions on Television Food Advertising to Children on Exposure to Advertisements for ‘Less Healthy’ Foods: Repeat Cross-Sectional Study. *PLoS One* 2012; **7**(2): e31578.

27. Campos D, Hernández-Torres J, Agil A, et al. Analysis of food advertising to children on Spanish television: probing exposure to television marketing. *Arch Med Sci* 2016; **12**(4): 799-807.

28. Dillman Carpentier FR, Correa T, Reyes M, Taillie LS. Evaluating the impact of Chile's marketing regulation of unhealthy foods and beverages: pre-school and adolescent children's changes in exposure to food advertising on television. *Public Health Nutrition* 2020; **23**(4): 747-55.

29. Hebden LA, King L, Grunseit A, Kelly B, Chapman K. Advertising of fast food to children on Australian television: the impact of industry self-regulation. *Medical Journal of Australia* 2011; **195**(1): 20-4.

30. Kim S, Lee Y, Yoon J, Chung S, Lee S, Kim H. Restriction of television food advertising in South Korea: impact on advertising of food companies. *Health Promotion International* 2013; **28**(1): 17-25.

31. Mediano Stoltze F, Reyes M, Smith TL, Correa T, Corvalán C, Carpentier FRD. Prevalence of Child-Directed Marketing on Breakfast Cereal Packages before and after Chile's Food Marketing Law: A Pre- and Post-Quantitative Content Analysis. *International Journal of Environmental Research and Public Health* 2019; **16**(22).

32. UK Office of Communications. Changes in the nature and balance of television food advertising to children: a review of HFSS advertising restrictions. Ofcom: London. 2008.

33. UK Office of Communications. HFSS advertising restrictions: Final Review. Ofcom: London. 2010.

34. Galloway DP, Calvert SL. Media characters as spokespeople in U.S. grocery stores: Promoting poor nutritional messages to children. *Journal of Obesity & Weight Loss Therapy* 2014; **4**: 212.

35. Vaala SE, Ritter MB. Child-Oriented Marketing on Cereal Packaging: Associations With Sugar Content and Manufacturer Pledge. *Journal of Nutrition Education and Behavior* 2020; **52**(3): 215-23.

36. Potvin Kent M, Dubois L, Kent EA, Wanless AJ. Internet marketing directed at children on food and restaurant websites in two policy environments. *Obesity (Silver Spring)* 2013; **21**(4): 800-7.

37. Clark CR. Advertising Restrictions and Competition in the Children's Breakfast Cereal Industry. *The Journal of Law & Economics* 2007; **50**(4): 757-80.

38. Otten JJ, Saelens BE, Kapphahn KI, et al. Impact of San Francisco's toy ordinance on restaurants and children's food purchases, 2011-2012. *Prev Chronic Dis* 2014; **11**: E122-E.

39. Silva A, Higgins LM, Hussein M. An Evaluation of the Effect of Child-Directed Television Food Advertising Regulation in the United Kingdom. *Canadian Journal of Agricultural Economics* 2015; **63**(4): 583-600.

40. Morton H, Stanton R, Zuppa J, Mehta K. Food advertising and broadcasting legislation--a case of system failure? *Nutrition & Dietetics* 2005; **62**: 26-.

41. Potvin Kent M, Dubois L, Wanless A. Food marketing on children's television in two different policy environments. *International Journal of Pediatric Obesity* 2011a; **6**(2-2): e433-41.

42. Potvin Kent M, Dubois L, Wanless A. A nutritional comparison of foods and beverages marketed to children in two advertising policy environments. *Obesity (Silver Spring)* 2012; **20**(9): 1829-37.

43. Dhar T, Baylis K. Fast-Food Consumption and the Ban on Advertising Targeting Children: The Quebec Experience. *Journal of Marketing Research* 2011; **48**(5): 799-813.

44. Whalen R, Harrold J, Child S, Halford J, Boyland E. Children's exposure to food advertising: the impact of statutory restrictions. *Health Promot Int* 2019; **34**(2): 227-35.
